# Water-Soluble Girard T‑Based Multidentate Ligands for Highly Selective Uranyl(VI) Binding

**DOI:** 10.1021/acs.inorgchem.6c03289

**Published:** 2026-09-07

**Authors:** Angelos Amoiridis, Matteo Marafante, Odysseas D. Keramidas, Epameinondas Leontidis, Silvia Berto, Nuno A. G. Bandeira, Haralampos N. Miras, Anastasios Keramidas

**Affiliations:** † Department of Chemistry, 54557 University of Cyprus, 2109 Nicosia, Cyprus; ‡ Department of Health Sciences, School of Life & Health Sciences, University of Nicosia, 2417 Nicosia, Cyprus; § Dipartimento di Chimica, 9314 Università di Torino, Via P. Giuria 7, 10125 Torino, Italy; ∥ Biosystems and Integrative Sciences Institute (BioISI)*-* Departamento de Química e Bioquímica, Faculdade de Ciências Universidade de Lisboa, 8.5.53 - C8 Campo Grande, 1749-016 Lisboa, Portugal; ⊥ School of Chemistry, 3526 The University of Glasgow, Glasgow G12 8QQ, U.K.

## Abstract

The development of chelating agents capable of selectively binding UO_2_^2+^ in aqueous media remains a major challenge in uranium recovery and remediation, largely due to competitive complexation by vanadium and iron ions. Here, we introduce two water-soluble Girard-T-based acylhydrazone ligands, a planar pentadentate 2,2′-(((1*E*,1′*E*)-pyridine-2,6-diylbis­(ethan-1-yl-1-ylidene))­bis­(hydrazin-1-yl-2-ylidene))­bis­(*N*,*N*,*N*-trimethyl-2-oxoethan-1-aminium) (H_2_dapGT^2+^) and tetradentate 2,2′-(((1*E*,2*E*)-ethane-1,2-diylidene)­bis­(hydrazin-1-yl-2-ylidene))­bis­(*N*,*N*,*N*-trimethyl-2-oxoethan-1-aminium) (H_2_glyxGT^2+^), designed to maximize equatorial coordination of the uranyl ion. The reaction of U^VI^O_2_^2+^ and Fe^III^ with H_2_dapGT^2+^ and H_2_glyxGT^2+^ led to the synthesis of [U^VI^O_2_(dapGT)­(H_2_O)]­[ClO_4_]_2_ (**1**), [U^VI^O_2_(dapGT)­MeOH]­[SbF_6_]_2_ (**1′**), [{U^VI^O_2_(dapGT)}_2_(*μ*-V^V^_4_O_12_)]·8H_2_O (**2**·8H_2_O), [U^VI^O_2_(glyxGT)­(CH_3_COO)]­ClO_4_ (**3**), [Fe^III^(HdapGT^2+^)­Cl_2_]­[ClO_4_]_2_ (**4**) and [{Fe^III^(dapGT^2+^)}_2_(*μ-*O)­(CH_3_OH)­(H_2_O)]­[ClO_4_]_4_ (**5**). Single-crystal X-ray diffraction reveals that dapGT^2+^ occupies five from the six available sites of the uranyl equatorial plane to form highly stable hexagonal bipyramidal complexes, while the corresponding Fe^III^ complexes adopt pentagonal bipyramidal geometries. Combined spectroscopic, thermodynamic, and theoretical studies show that dapGT^2+^ exhibits exceptional stability and pronounced selectivity for U^VI^O_2_^2+^ over V^V^O^2+^, outperforming amidoxime-based chelators under competitive conditions. In contrast, the more flexible glyxGT^2+^ ligand forms weaker but still selective uranyl complexes. These findings establish ligand denticity and equatorial plane multidentate ligation as key design principles for achieving uranyl selectivity in water and provide a viable framework for next-generation actinide chelators relevant to uranium extraction, waste management, and actinide sequestration.

## Graphical abstract

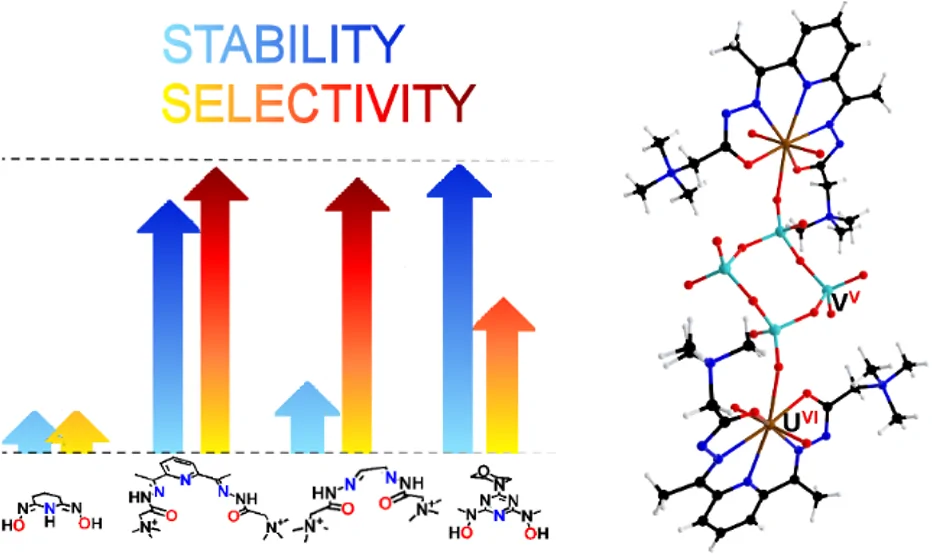

## Introduction

The development of chelating agents capable of selectively binding hard metal ions has attracted sustained interest due to applications in environmental remediation, radioactive waste management, and metal recovery from aqueous media. In particular, the selective sequestration of UO_2_^2+^ remains a central challenge in uranium extraction from seawater and in biological detoxification strategies, owing to its low concentration and strong competition from other hard metal ions.^1–27^ While numerous ligand classes have been explored, achieving both high stability and selectivity for UO_2_^2+^ under aqueous conditions remains elusive.^7,8,18,21–24,26,28–46^

The high hydrolytic stability of amidoximate–(U^VI^O_2_^2+^) under neutral and alkaline conditions, compared to U^VI^O_2_^2+^ carboxylate/phenolate complexes, and the low synthetic cost have heightened research interest in their potential application in mining U^VI^O_2_^2+^ from seawater. However, U^VI^O_2_^2+^ amidoximate complexes exhibit insufficient selectivity for uranyl binding in the presence of other hard metal ions, notably V^V^ and Fe^III^ which are the main competing metal ions present in seawater.^8,37,47–53^ Moreover, amidoxime degradation in the presence of Mo^VI^ further compromises their performance in marine environments.^54,55^ These limitations highlight the need for alternative ligand designs that prioritize selectivity rather than binding strength alone.

From a coordination chemistry perspective, selective U^VI^O_2_^2+^ binding requires ligands that complement both the hard Lewis acidity, exhibit appropriate electron-donating strength, and accommodate the geometric preferences of the metal ion.^18,26,39^ The equatorial plane of U^VI^O_2_^2+^ is the sole site available for coordination. Consequently, planar ligands with multiple hard donor atoms such as nitrogen and oxygen fulfill the ligation requirements for the selective binding of U^VI^O_2_^2+^ (Figure 1). In contrast, competing metal ions such as V^V^O^2+^ possess fewer available coordination sites and distinct geometric preferences, suggesting that equatorial plane multidentate coordination may disfavor their binding. These considerations have motivated the exploration of multidentate, planar ligand frameworks as a strategy for selective uranyl chelation.

**Figure 1:**
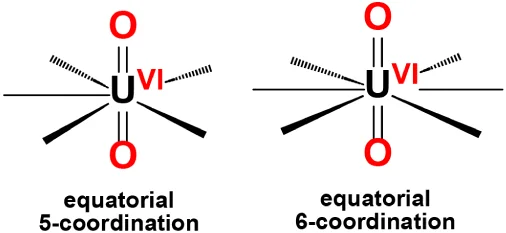
Coordination modes of the U^VI^O_2_^2+^ structural unit.

Girard-T-based acylhydrazone ligands, 2,6-dipicolinic acid, (2*Z*,6*Z*)-piperidine-2,6-dione dioxime, and *N*,*N*′-disubstituted bis­(hydroxyamino)-1,3,5-triazine (BHT) (Figure 2) meet these criteria. Specifically, BHT ligands, owing to the negative formal charge of the triazine nitrogen donor atoms, form hydrolytically very stable complexes with hard metal ions, including Fe^III^, Ti^IV^, V^V^, U^VI^, and Mo^VI^.^3,10–14,39,56–59^ Tridentate ligands typically exhibit limited binding selectivity in distinguishing between U^VI^O_2_^2+^ and V^V^O_2_^+^. However, H_2_bihyat (Figure 2A, BHT-type ligand) demonstrated notable selectivity for bonding with U^VI^O_2_^2+^. The selectivity of BHT ligands depends on the electron donor strength of the BHT moiety. Enhancing the electron donor strength of the BHT moiety increases ion binding selectivity towards V^V^O_2_^+^ compare to U^VI^O_2_^2+^.^39^ Given the greater number of available U^VI^O_2_^2+^ coordination sites compared to V^V^O_2_^+^, selectivity can be enhanced by employing flat ligands that contain more than three hard donor atoms.^22,23,26,37,60,61^ Crystallographic characterization and speciation studies of uranyl complexes with pentadentate ligands have become more common, though they are still relatively scarce.^27,62–82^ Among these, the most extensively studied are uranyl complexes coordinated with amine-phenol pentadentate ligands, as illustrated in Figure 2B. Additionally, stability studies involving tetradentate ligands with uranyl remain infrequent.^83–87^

**Figure 2:**
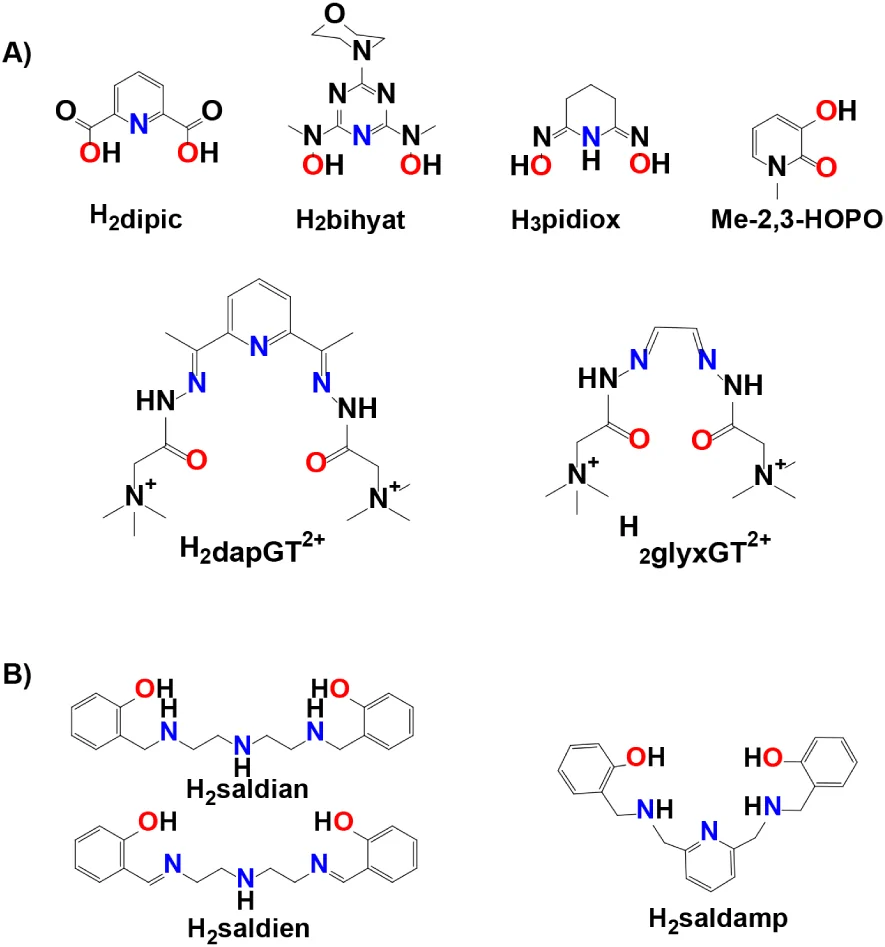
Molecular drawings of the ligands: A) 2,6-dipicolinic acid (H_2_dipic), 2,6-bis­[hydroxy­(methyl)­amino]-4-morpholino-1,3,5-triazine (H_2_bihyat), (2*Z*,6*Z*)-piperidine-2,6-dione (H_3_pidiox), Me-2,3-HOPO, 2,2′-(((1*E*,1′*E*)-pyridine-2,6-diylbis­(ethan-1-yl-1-ylidene))­bis­(hydrazin-1-yl-2-ylidene))­bis­(*N,N,N*-trimethyl-2-oxoethan-1-aminium) H_2_dapGT^2+^, 2,2′-(((1*E*,2*E*)-ethane-1,2-diylidene)­bis­(hydrazin-1-yl-2-ylidene))­bis­(*N,N,N*-trimethyl-2-oxoethan-1-aminium) H_2_glyxGT^2+^. B) Additional amino-phenol pentadentate ligands reported in the literature.^74,81,82^

Herein, we report the synthesis and comprehensive characterization of two water-soluble Girard-T-based acylhydrazone ligands: the full delocalized planar pentadentate 2,2′-(((1*E*,1*′E*)-pyridine-2,6-diylbis­(ethan-1-yl-1-ylidene))­bis­(hydrazin-1-yl-2-ylidene))­bis­(*N*,*N*,*N*-trimethyl-2-oxoethan-1-aminium) (H_2_dapGT^2+^) and a more flexible tetradentate analogue 2,2′-(((1*E*,2*E*)-ethane-1,2-diylidene)­bis­(hydrazin-1-yl-2-ylidene))­bis­(*N*,*N*,*N*-trimethyl-2-oxoethan-1-aminium) (H_2_glyxGT^2+^) (Figure 2). The selection of Girard-T-based acylhydrazone ligands is predicated on two primary considerations: (A) their ability to enhance the solubility of their complexes in aqueous solutions, and (B) The presence of carbohydrazide groups, which independently exhibit weak interactions with metal ion centers, can serve as strong donors in chelate molecules. Consequently, the ligands are unable to form stable complexes with metal ions unless they coordinate in a multidentate mode. In contrast to U^VI^O_2_^2+^, V^V^O_2_^+^ cannot form multicoordinated complexes, increasing the ability of the ligand to preferentially bind U^VI^O_2_^2+^ over V^V^O_2_^+^.

Their coordination chemistry with U^VI^O_2_^2+^, Fe^III^, and V^V^O_2_^+^, species, [U^VI^O_2_(dapGT)­(H_2_O)]­[ClO_4_]_2_ (**1**), [U^VI^O_2_(dapGT)­MeOH]­[SbF_6_]_2_ (**1′**), [{U^VI^O_2_(dapGT)}_2_(*μ*-V^V^_4_O_12_)]·8H_2_O (**2**·8H_2_O), [U^VI^O_2_(glyxGT)­(CH_3_COO)]­ClO_4_ (**3**), [Fe^III^(HdapGT^2+^)­Cl_2_]­[ClO_4_]_2_ (**4**) and [{Fe^III^(dapGT^2+^)}_2_(*μ-*O)­(CH_3_OH)­(H_2_O)]­[ClO_4_]_4_ (**5**), and were further investigated through a combination of single-crystal X-ray diffraction, spectroscopic techniques and potentiometric and measurements, and density functional theory calculations. We demonstrate that the pentadentate dapGT ligand occupies five of the six coordination sites in the uranyl equatorial plane, forming highly stable complexes with notable selectivity for U^VI^O_2_^2+^ over competing vanadium ions. This performance significantly surpasses that of carboxylate and amidoxime-based chelators under competitive conditions.

In contrast, the tetradentate glyxGT ligand forms weaker but still selective uranyl complexes. Furthermore, owing to their water solubility and low toxicity,^88^ these compounds may be utilized in biological applications, such as the removal of U^VI^O_2_^2+^ and Fe^III^ from the human body.^22,23,26,89–93^ Together, these results establish equatorial-plane coordination by planar multidentate ligands as a robust design principle for achieving selective uranyl binding in aqueous media.

## Experimental Section

All chemicals were of reagent grade purity and were provided by Sigma-Aldrich unless otherwise stated. Methanol was dried over magnesium and distilled prior to use. *Standard precautions for handling uranium compounds and perchlorate salts were followed.*

### Synthesis of H_2_dapGTCl_2_

H_2_dapGTCl_2_ was synthesized using a method distinct from that described in the literature.^46,93^ A solution of Girard T reagent (GT) (1.70 g, 9.80 mmol) in methanol (15 mL) was introduced into a methanolic (15 mL) solution of 2,6-diacetylpyridine (0.800 g, 4.90 mmol). The resulting mixture was heated to 40 °C until a white solid was precipitated. Subsequently, the mixture was cooled to 25 °C. The solid was then isolated by vacuum filtration, washed with 10 mL diethyl ether, and dried, yielding 1.51 g (71%) of the desired product. Chemical Formula: C_19_H_33_Cl_2_N_7_O_2_. MW: 462.42 g/mol. Elemental Analysis, Calcd: C, 49.35; H, 7.19; N, 21.20, Found: C, 49.21; H, 7.25; N, 21.38.

### Synthesis of H_2_glyxGTCl_2_

This ligand was synthesized according to the procedure reported for H_2_dapGTCl_2_. In this synthesis, glyoxal (0.280 g, 4.90 mmol) was utilized in place of 2,6-diacetylpyridine, yielding 1.12 g (64%) of the desired compound. Chemical Formula: C_12_H_26_Cl_2_N_6_O_2_. Molecular Weight: 357.28 g/mol. Elemental Analysis, Calcd: C, 40.34; H, 7.34; N, 23.52, Found: C, 40.47; H, 7.51; N, 23.40.

**Synthesis of [U** ^**VI**^ **O**_**2**_ **(dapGT)­(H**_**2**_ **O)]­[ClO**_**4**_ **]**_**2**_ **(1)** U^VI^O_2_(CH_3_COO)_2_·2H_2_O (0.195 g, 0.460 mmol) was dissolved in methanol (10 mL), and solid H_2_dapGTCl_2_ (0.213 g, 0.460 mmol) was added to a single portion, resulting in a bright yellow solution. Subsequently, solid NaClO_4_ (0.116 g, 0.950 mmol) was added, leading to the immediate formation of a yellow precipitate. The mixture was stirred at 25 °C for 30 min, filtered, washed with cold methanol and diethyl ether, and dried under vacuum, yielding 0.36 g (89%). X-ray diffraction-quality single crystals of compound **1** were obtained through the slow evaporation of an aqueous solution of the yellow solid (8.5 mg in 2 mL of H_2_O). ^1^H NMR (D_2_O, 500 MHz, 25 °C) δ (ppm): 8.42 (m, 3*H*); 4.43 (s, 4*H*); 3.88 (s, 18*H*); 2.87 (s, 6*H*). ^13^C­{^1^H} NMR (D_2_O, 126 MHz, 25 °C) δ (ppm): 171.29, 168.85, 157.23, 142.78, 127.14, 67.11, 54.54, 15.61. Chemical Formula: C_19_H_33_Cl_2_N_7_O_13_U. MW: 876.44 g/mol. Elemental Analysis, Calcd: C, 26.04; H, 3.80; N, 11.19, Found: C, 26.21; H, 3.96; N, 11.11.

**Synthesis of [U** ^**VI**^ **O**_**2**_ **(dapGT)­MeOH]­[SbF**_**6**_ **]**_**2**_ **(1′)** U^VI^O_2_(CH_3_COO)_2_·2H_2_O (0.0970 g, 0.230 mmol) was dissolved in methanol (10 mL), and solid H_2_dapGTCl_2_ (0.100 g, 0.230 mmol) was added in a single portion. The resulting solution exhibited a bright yellow color. Subsequently, NaSbF_6_ (0.100 g, 0.40 mmol) was introduced to the solution, resulting in the formation of a yellow precipitate. The mixture was stirred at 25 °C, followed by filtration, washed with cold methanol and diethyl ether, and dried under reduced pressure yielding 0.16 g (60%). X-ray diffraction (XRD)-quality single crystals of compound **1′** were obtained through the slow diffusion of diethyl ether vapor into the filtered yellow solution over a period of three days at 25 °C. ^1^H NMR (D_2_O, 500 MHz, 25 °C) δ (ppm): 8.42 (m, 3*H*); 4.43 (s, 4*H*); 3.88 (s, 18*H*); 2.87 (s, 6*H*). ^13^C­{^1^H} NMR (D_2_O, 126 MHz, 25 °C) δ (ppm): 171.29, 168.85, 157.23, 142.78, 127.14, 67.11, 54.54, 15.61. Chemical Formula: C_20_H_35_F_12_N_7_O_5_Sb_2_U. MW: 1163.07 g/mol. Elemental Analysis, Calcd: C, 20.65; H, 3.03; N, 8.43, Found: C, 20.73; H, 3.12; N, 8.39.

**Synthesis of [{U** ^**VI**^ **O**_**2**_ **(dapGT)}**_**2**_ **(** ***μ*** **-V** ^**V**^_**4**_ **O**_**12**_ **)]·8H**_**2**_ **O (2·8H**_**2**_ **O)** An aqueous solution (2 mL) of compound **1** (0.010 g, 0.011 mmol) was combined with an aqueous solution (0.5 mL) of NaV^V^O_3_ (0.032 g, 0.026 mmol). Upon mixing, the pH of the yellow solution was measured at 7.0. Over the course of 1 h, a yellow crystalline solid precipitated. Recrystallization followed by dissolving the precipitate in small amount of water and heating at 100 °C, followed by slow cooling over 24 h, yielding 0.0082 g (77%) single crystals of **2**·8H_2_O suitable for X-ray analysis. Chemical Formula: C_38_H_77_N_14_O_28_U_2_V_4_. MW: 1857.93 g/mol. Elemental Analysis, Calcd: C, 24.57; H, 4.18; N, 10.55, Found: C, 24.69; H, 4.24; N, 10.44.

**Synthesis of [U** ^**VI**^ **O**_**2**_ **(glyxGT)­(CH**_**3**_ **COO)]­[ClO**_**4**_ **] (3)** In a stirring methanolic (10 mL) solution of H_2_glyxGT·Cl_2_ (0.050 g, 0.14 mmol), solid U^VI^O_2_(CH_3_COO)_2_·2H_2_O (0.059 g, 0.14 mmol), and NaClO_4_ (0.034 g, 0.28 mmol) were introduced. The resulting yellow solution was stirred for 24 h forming a yellow solid, which was subsequently isolated via filtration, washed with a minimal amount of methanol, and dried under reduced pressure yielding 0.068 g (68%). A small portion of the solid was dissolved in DMF, and the solution was subjected to slow diffusion with diethyl ether vapor. After several days, yellow crystals suitable for X-ray crystallography were observed. ^1^H NMR (D_2_O, 500 MHz, 25 °C) δ (ppm): 8.76 (s, 2*H*); 4.41 (s, 4*H*); 3.39 (s, 18*H*). ^13^C­{^1^H} NMR (D_2_O, 126 MHz, 25 °C) δ (ppm): 174.46, 154.91, 66.52, 54.00, 52.00. Chemical Formula: C_14_H_27_ClN_6_O_10_U. MW: 712.88 g/mol. Elemental Analysis, Calcd: C, 23.59; H, 3.82; N, 11.79, Found: C, 23.70; H, 3.88; N, 11.68.

**Synthesis of [Fe** ^**III**^ **(HdapGT)­Cl**_**2**_ **]­[ClO**_**4**_]_**2**_ **(4)** To a suspension of H_2_dapGTCl_2_ (0.200 g, 0.460 mmol) in methanol (10 mL), an equivalent amount of Fe^III^(ClO_4_)_3_·H_2_O (0.171 g, 0.460 mmol) was added, and the mixture was refluxed for 2 h. The resulting dark-red solution was filtered, yielding the initial crop of compound **4** as a green solid. Subsequently, diethyl ether was introduced into the dark red filtrate through slow diffusion, resulting in the formation of X-ray diffraction-quality dark-green crystals of compound **4**, yielding 0.125 g (38%). Chemical Formula: C_19_H_33_Cl_4_FeN_7_O_10_. MW: 717.16 g/mol. Elemental Analysis, Calcd: C, 31.82; H, 4.64; N, 13.67, Found: C, 31.80; H, 4.61; N, 13.68.

**Synthesis of [{Fe** ^**III**^ **(dapGT** **)}**_**2**_ **(** ***μ-*** **O)­(CH**_**3**_ **OH)­(H**_**2**_ **O)]­[ClO**_**4**_]_**4**_ **·4CH**_**3**_ **OH·H**_**2**_ **O (5·4CH**_**3**_ **OH·H**_**2**_ **O)** H_2_dapGTCl_2_ (0.200 g, 0.460 mmol) and triethylamine (0.480 mL, 3.46 mmol) were dissolved in methanol (10 mL). Subsequently, Fe^III^(ClO_4_)_3_·H_2_O (0.171 g, 0.460 mmol) was introduced to the colorless solution, resulting in an immediate transformation to a bright orange hue. The orange solution was refluxed for 2 h. Slow diffusion of diethyl ether into the orange solution afforded large, rod-shaped bright orange crystals of **5**·4CH_3_OH·H_2_O, suitable for single-crystal X-ray diffraction analysis, yielding 0.249 g (72%). Chemical Formula: C_43_H_90_Cl_4_Fe_2_N_14_O_28_. MW: 1504.75 g/mol. Elemental Analysis, Calcd: C, 34.32; H, 6.03; N, 13.03, Found: C, 34.39; H, 5.98; N, 13.29.

## Results and Discussion

**Synthesis of organic molecules H**_**2**_ **dapGTCl**_**2**_ **and H**_**2**_ **glyxGTCl**_**2**_ **and coordination compounds 1–5.** The acylhydrazone ligands based on Girard-T reagent, H_2_dapGTCl_2_ and H_2_glyxGTCl_2_, were synthesized through the condensation of aldehydes or ketones with Girard-T reagent in anhydrous methanol, as depicted in Scheme 1. The synthesis of H_2_dapGTCl_2_ has been previously reported in the literature; however, in this work the isolated product was obtained as the tetrahydrate, crystallizing as H_2_dapGTCl_2_·4H_2_O.^46,93^ The uranyl complexes were synthesized through the direct reaction of equimolar amount of U^VI^O_2_(CH_3_COO)_2_·2H_2_O and organic ligands in methanol, as depicted in Scheme 2. Although the ligands were sparingly soluble in methanol, their coordination to the uranyl ion resulted in their complete dissolution. The ionic nature of the resulting complexes allows chloride (Cl^–^) counterions to be exchanged for either perchlorate (ClO_4_^–^) or hexafluoroantimonate (Sb^V^F_6_^–^), leading to precipitation of the corresponding salts.

**Scheme 1:**
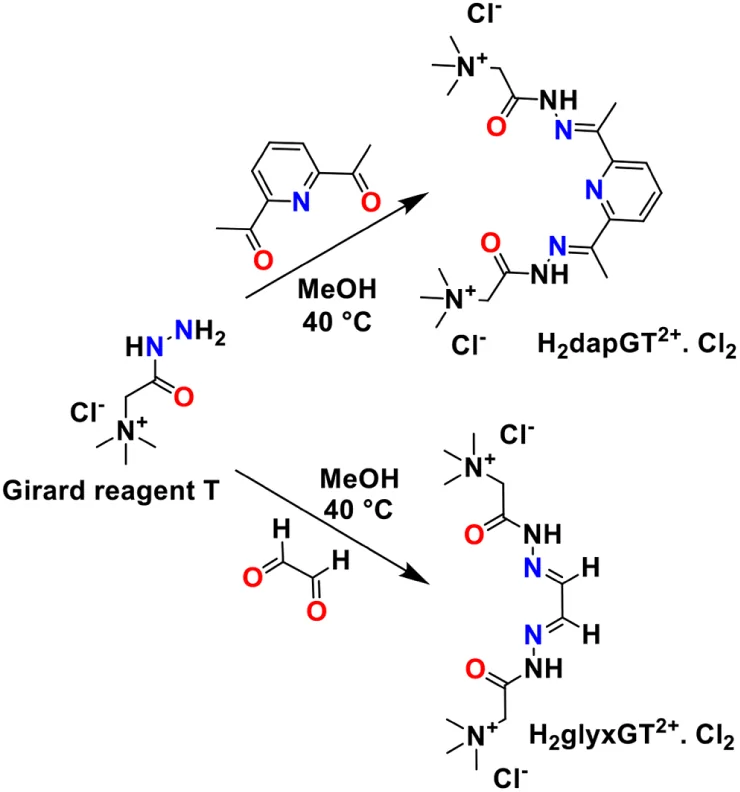
Synthesis of Ligands H_2_dapGT^2+^ and H_2_glyxGT^2+^

**Scheme 2:**
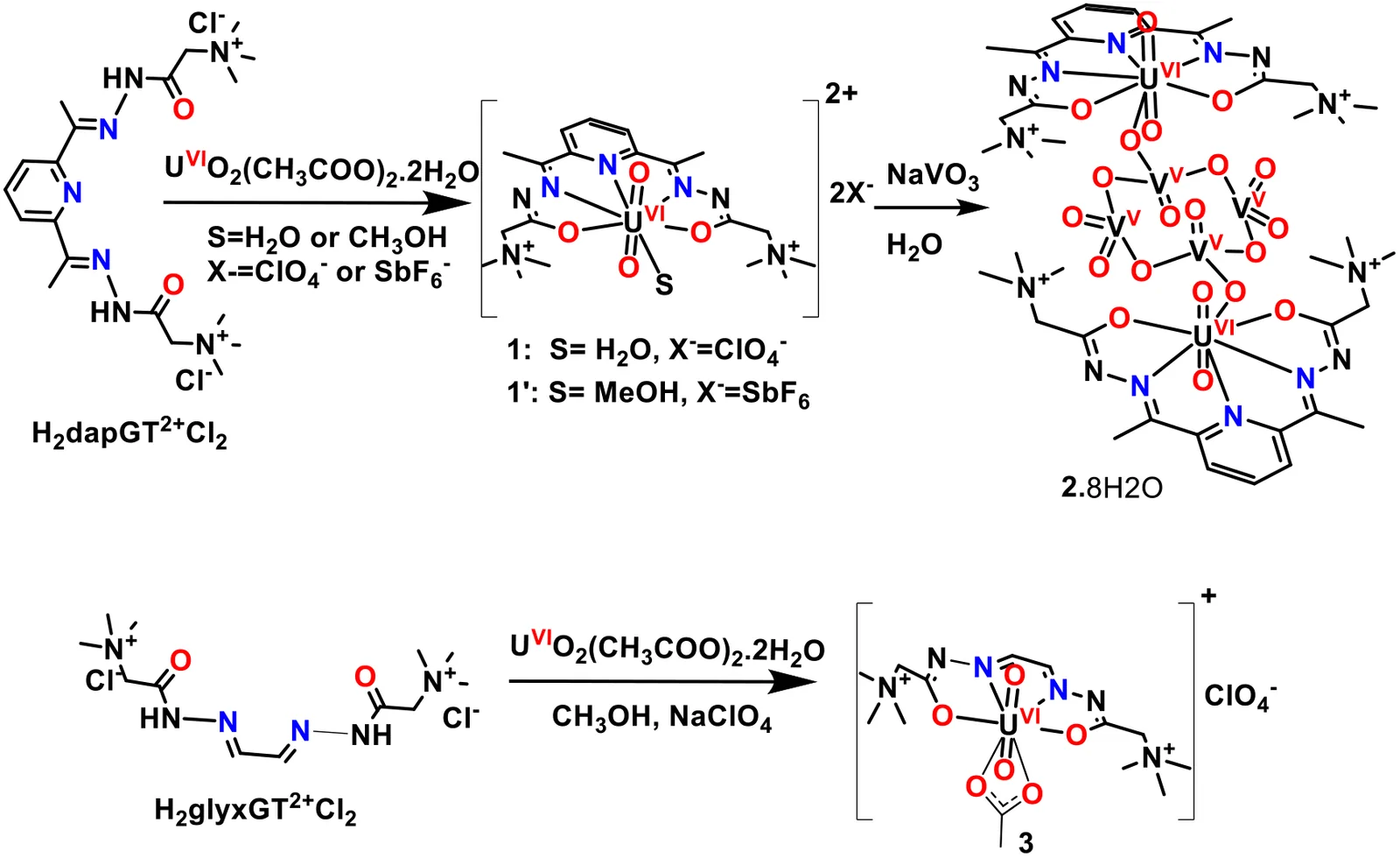
Synthesis of Compounds **1**, **1′**, **2**·8H_2_O and **3**

The iron-containing complex **4** was synthesized by a metathesis reaction of equimolar amounts of Fe^III^(ClO_4_)_3_ and dapGT in methanol (Scheme 3). The addition of triethylamine to the solution resulted in the formation of dinuclear Fe^III^–O–Fe^III^ dapGT complex **5**, generated through hydroxide-mediated bridging of two Fe^III^ centers.

**Scheme 3:**
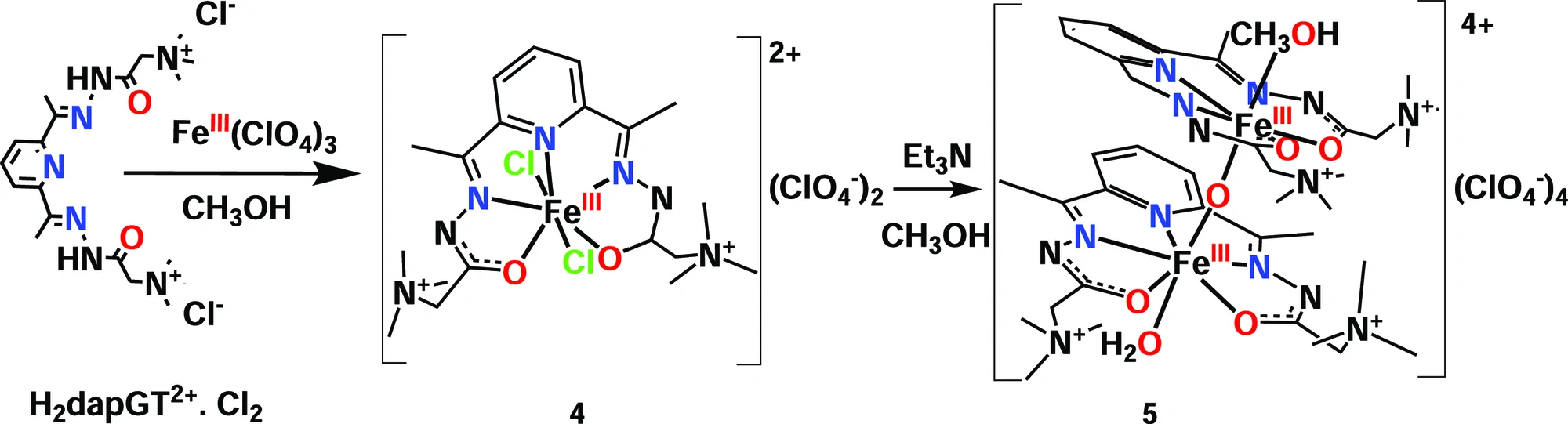
Synthesis of Complexes **4** and **5**·**** 4CH_3_OH**·**H_2_O

Heterometallic complex **2** was obtained by mixing **1** with two equivalents of NaV^V^O_3_ in water. The cyclic tetranuclear vanadate moiety (V_4_O_12_^4–^, V_4_) acts as a bridge between two [U^VI^O_2_(H_2_dapGT)]^2+^ ions and as counter anions. This structure appears to be unstable in solution, because the ^1^H NMR spectrum of **2** is identical to that of **1**, while the ^51^V NMR spectrum shows signals corresponding to free vanadate oligomers. For both ligands, all attempts to isolate and characterize new V^V^ complexes were unsuccessful.

To the best of our knowledge, no crystallographically characterized complexes featuring planar pentadentate ligands coordinated to oxo– or dioxo–V^V^ centers have been previously reported in the literature.^94,95^ NMR and ESI-MS studies in D_2_O indicate the formation of a range of vanadium species that are unstable in solution.

### Characterization of Complexes

#### X-ray Crystallography

A comprehensive summary of the crystallographic data, final refinement details, interatomic distances and bond angles for complexes **1**–**3** are presented in Tables S1–S4. Structural parameters for the U^VI^ and V^V^ coordination spheres are listed in Table S2. ORTEP plots illustrating the crystal structures of complexes **1**, **1′**, **2**, and **3** are depicted in Figures 3 and S1.

**Figure 3:**
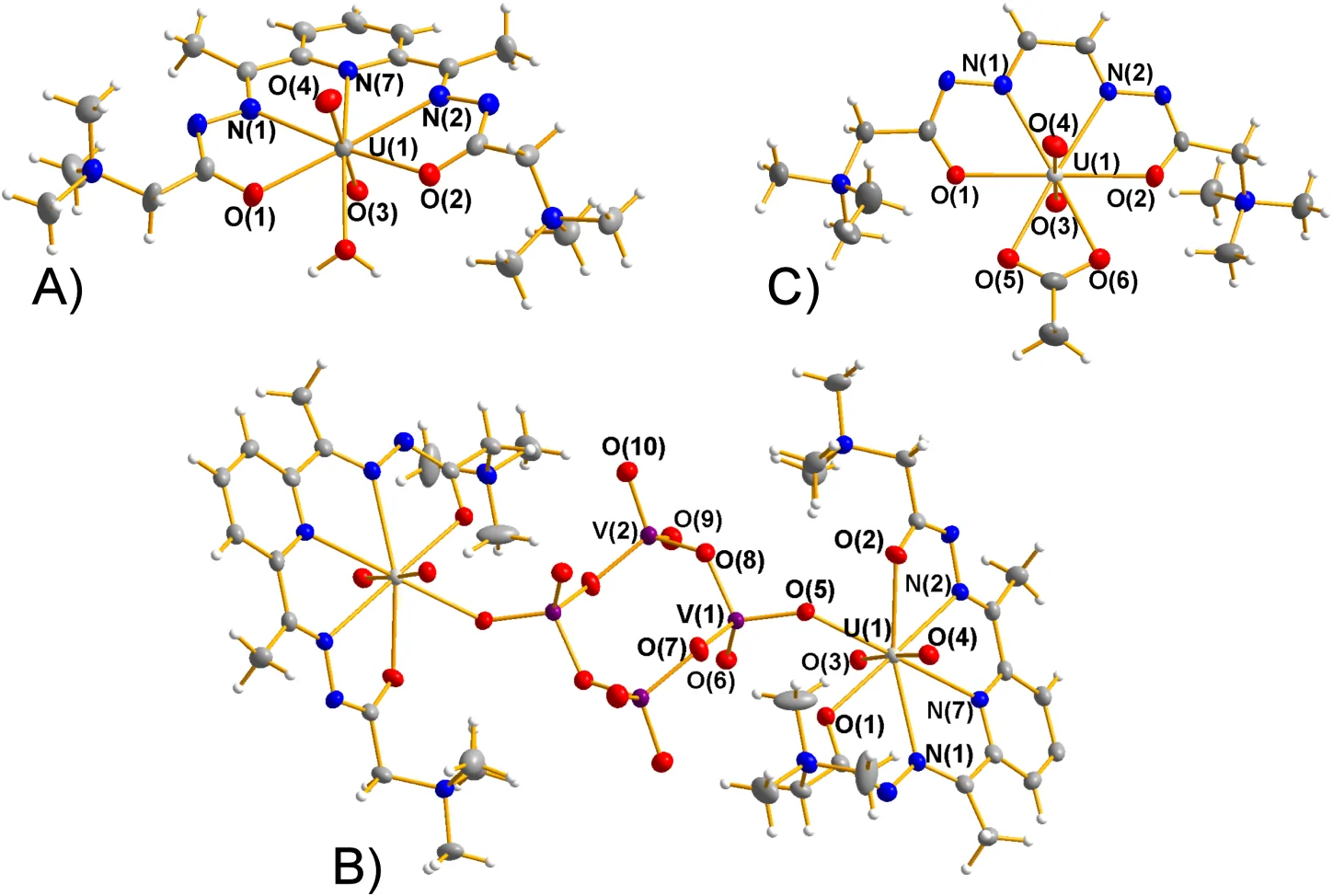
ORTEP plots of (A) **1** (B) **2** and (C) **3** with 50% thermal ellipsoids. Counter anions have been omitted for clarity.

Interestingly, all four uranium complexes adopted a hexagonal bipyramidal coordination geometry. The equatorial plane of U^VI^ in complexes **1**, **1′**, and **2** is defined by the five donor atoms of dapGT, comprising three nitrogen atoms and two oxygen atoms, along with the oxygen donor atom from either H_2_O, CH_3_OH, or V^V^_4_O_12_^4–^, respectively. The two axial positions were occupied by two oxido (=O) atoms [O(3) and O(4)]. The distances of the U^VI^ ion from the plane defined by the five dapGT donor atoms were 0.1192(3), 0.0131(2), and 0.1461(2) Å for complexes **1**, **1′**, and **2**, respectively. The V^V^ ion in the cyclic tetranuclear V^V^_4_O_12_^4–^ anion in complex **2** was characterized by a tetrahedral environment defined by two oxido groups (=O) and two *μ*-O atoms. The V^V^_4_O_12_^4–^ anion bridges two [UO_2_(dapGT)]^2+^ moieties through two oxido groups, (V^V^=O)–U^VI^. The coordination of U^VI^ results in an elongation of approximately 0.06 Å in the V^V^=O bond.

The equatorial plane of U^VI^ in **3** is defined by the four donor atoms, specifically two nitrogen atoms and two oxygen atoms of glyxGT, along with the two oxygen atoms of a chelate-coordinated *κ* ^2^-(CH_3_COO^–^). The axial positions were occupied by two oxido groups, O(3) and O(4). The distance of the U^IV^ from the plane defined by N(1), N(2), O(1), and O(2) was 0.1037(3) Å.

U­(1)–O­(1) and U(1)–O(2) represent the shortest bond distances within the equatorial plane of complexes **1**, **1′**, **2**, and **3**, with measurements ranging from 2.37 to 2.42 Å. These distances are comparable to the U^V^–O_carboxylate_ bond lengths reported in the literature.^96,97^ The U–O­(1,2) bond lengths are more than 0.1 Å shorter than those of U–N, U–O_solvent_, U–O_V4O12_, and U–O_CH3COO_, indicating their greater bond strength. Based on the observed short distances between *d*_U(1)–O(1)_ and *d*_U(1)–O(2)_, it appears unlikely that O(1) and O(2) functioned as carbonyl oxygen atoms within the complexes. Instead, they are more likely to be identified as enolic.^98^ The dapGT and glyxGT ligands can assume three resonance forms, I, II, and III, as depicted in Scheme 4. The average bond lengths of C(5)–N(3) and C(8)–N(5), as well as C(5)–O(1) and C(8)–O(2), are 1.30 Å and 1.28 Å, respectively, indicating a bond character of one and a half for both. This implies that the electronic structure of the coordinated ligands corresponds to an intermediate resonance state between forms I and III.

**Scheme 4:**
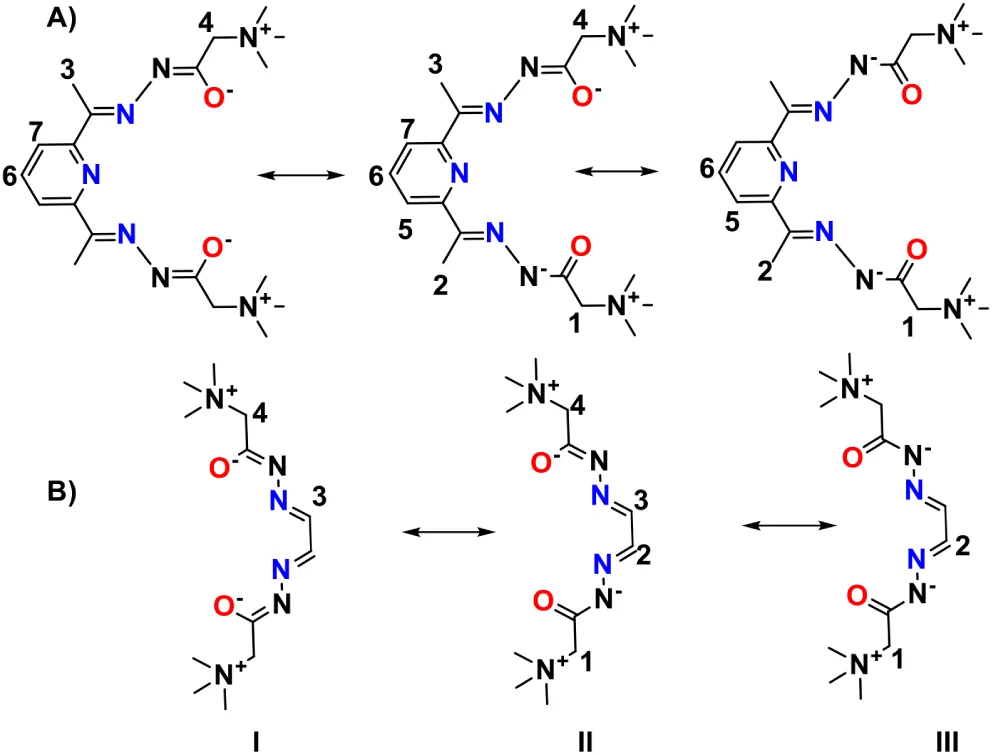
Resonance Forms and Atom Numbering (for ^1^H and ^13^C­{^1^H} NMR) of (A) dapGT and (B) glyxGT

Table S3 provides a detailed summary of the crystallographic data and final refinement parameters of complexes **4** and **5**. Table S4 lists the interatomic distances and bond angles that are significant for the Fe^III^ coordination spheres. In Figure 4 the ORTEP diagrams of the crystal structures of complexes **4** and **5** is shown.

**Figure 4:**
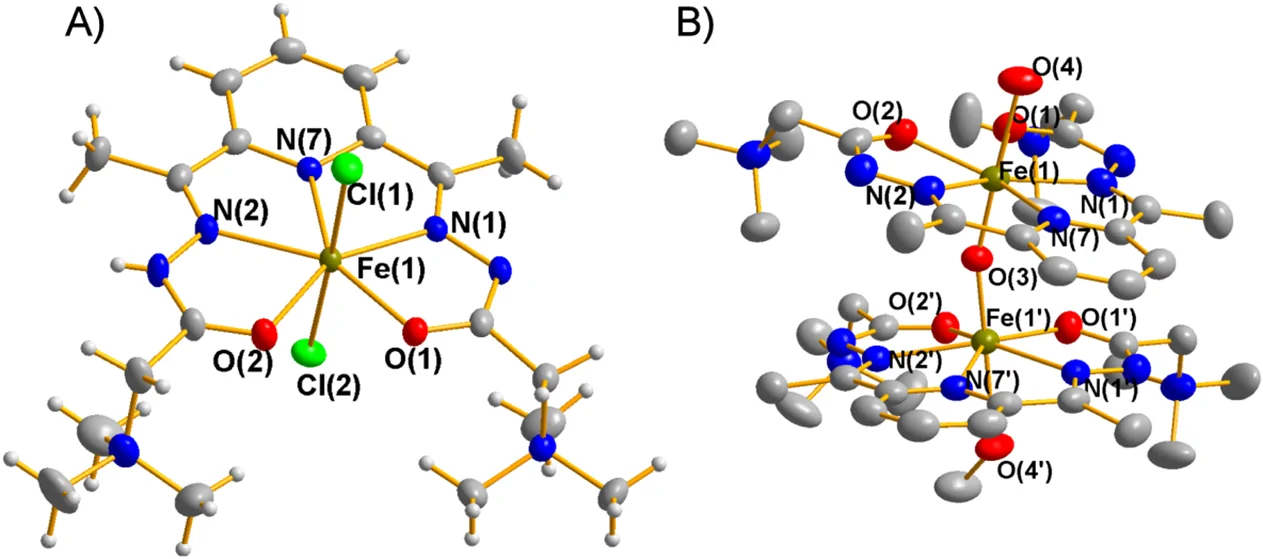
ORTEP plot of (A) **4** and (B) **5** with 50% thermal ellipsoids. Counter anions have been omitted for clarity.

The crystal structures of complexes **4** and **5** exhibited a pentagonal bipyramidal geometry. In these structures, three nitrogen and two oxygen donor atoms from HdapGT^+^ and dapGT defined the equatorial plane for complexes **4** and **5**, respectively, with mean distances of *d*_mean_ [Fe(1)–O] = 2.140(3) Å and *d*_mean_ [Fe(1)–N] = 2.189(3) Å. In complex **4**, two chlorine atoms occupied the axial positions, with a mean distance of d_mean_ [Fe(1)–Cl] = 2.347(5) Å and an angle of Cl(1)–Fe(1)–Cl(2) = 178.01(4)°. In complex **5**, the axial positions of the two Fe nuclei were occupied by μ-O and the oxygen atoms of methanol and water for Fe(1) and Fe(1′), respectively. The short Fe–O(3) distance [1.791(3) Å] and large Fe(1)–O(3)–Fe(1′) angle [162.6(1)°] suggest a double bond character for the Fe–(*μ*-O) bonds. The distance of Fe^III^ from the plane defined by N(1), N(2), O(1), and O(2) was 0.0525(6) Å for complex **4** and 0.2541(5) and 0.2244(5) Å for complex **5**.

The ketone-enol character of the acylhydrazone ligands in the complexes was confirmed by IR spectroscopy (Figure S2).

### NMR Characterization of the Ligands and Complexes

Although both organic ligands, H_2_dapGT^2+^ and H_2_glyxGT^2+^, were confirmed to be 99.9% pure by ESI-MS (Figure S3) and elemental analysis, their ^1^H NMR spectra in D_2_O displayed a greater number of peaks than expected. The synthesis of H_2_dapGT^2+^ has been previously documented.^46,99–101^ However, H_2_dapGT^2+^ has not been characterized using ^1^H NMR spectroscopy.

The complexities observed in the ^1^H NMR spectra of the ligands can be attributed to the presence of tautomers, which result from enolization of the carbonyl oxygen atom. The ^1^H NMR signals corresponding to the three tautomers (Figures S4 and S5) were anticipated for both ligands, a finding corroborated by 2D {^1^H}*gr*COSY and {^1^H, ^13^C}*gr*HSQC NMR spectroscopy (Figures S6–S8). An increase in pD shifts the equilibrium between the three tautomers toward the enolic species (Figure S9). The 2D {^1^H}*gr*EXSY NMR spectra revealed exchange between tautomers a/b and c/b (Figure S10). The a/c and b/b cross-peaks were not observed, as the simultaneous tautomerization of two moieties within the same molecule is statistically improbable.

The ^1^H NMR spectra of D_2_O solutions of compounds **1** and **3** are shown in Figure S11. Unlike the spectra of the free ligands, the spectra of the complexes displayed peaks solely from the I resonance form (Scheme 4), which was consistent with the solid-state structure of the complexes. Coordination of both dapGT and glyxGT to U^IV^O_2_^2+^ resulted in a downfield shift of the peaks.

As expected, the spectrum of compound **1** displayed signals for the peaks of the aromatic protons (8.451 ppm), which exhibit second-order splitting. Three additional peaks at 4.429, 3.881, and 2.870 ppm were attributed to −CH_2_– (4), the methyl groups of −N^+^(CH_3_)_3_ (8), and −CH_3_ (3) of 2,6-diacetylpyridine, respectively. The ^1^H NMR spectrum of the D_2_O solution of compound **3** exhibited three signals at 8.761, 4.408, and 3.393 ppm, assigned to the glyoxal (3), −CH_2_– (4), and −N^+^(CH_3_)_3_ (8) methyl protons, respectively (Table 1).

**Table 1:** ^1^H (^13^C­{^1^H}) Chemical Shifts (ppm) of the D_2_O Solutions at pD = 7.0 of H_2_dapGT^2+^, H_2_glyxGT^2+^, **1**, and **3** (^13^C NMR Shifts from 2D {^1^H, ^13^C} grHMQC)

compd	1	2	3	4	5	6	7	8
H_2_dapGT^2+^	4.692 (63.69)	2.349 (12.20)	2.423 (13.91)	4.285 (64.55)	(**a**) 7.905 (121.70)	(**a**) 7.775 (137.94)	(**b**) 7.927 (122.26)	3.313 (55.21)
					(**b**) 7.901 (122.40)	(**b**) 7.796 (138.19)	(**c**) 7.936 (122.86)	
						(**c**) 7.840 (138.42)		
**1**			2.870 (15.54)	4.429 (67.04)		8.417 (142.72)	8.417 (127.08)	3.881 (54.47)
H_2_glyxGT^2+^	(**a**) 4.512 (62.83)	(**a**) 7.524 (144.38)	(**b**) 7.730 (148.52)	(**b**) 4.113 (63.59)				3.224 (54.21)
	(**b**) 4.525 (62.83)	(**b**) 7.559 (143.63)	(**c**) 7.759 (147.82)	(**c**) 4.125 (63.55)				
**3**			8.761 (154.91)	4.408 (66.52)				3.393 (54.52)

### Hydrolytic Stability and Speciation Studies of the Complexes **1**–**5**

#### ^1^H NMR Investigation of the Hydrolytic Stability of Complexes **1** and **3**

The ^1^H NMR spectra of **1** and **3** at various pDs are shown in Figures S12 and S13, respectively. The spectra indicate that **1** remains hydrolytically stable at a pH range of 3–10 for concentrations 1.0 and 4.0 mM. Complex **3** maintains stability at pDs 5–8, releases free ligands at acidic pDs below pD = 5.0, and undergoes partial hydrolysis to form species [U^VI^(O)_2_(*κ* ^3^-glyxGT)­(D_2_O)­(OD)]^+^ (**3a**) or [U^VI^(O)_2_(*κ* ^3^-glyxGT)­(OD)_2_] at pDs 8–10 (Figure 5). The asymmetry of complex **3a** resulted in a doubling of the peaks compared to the symmetric **3**. The 2D {^1^H} grCOSY spectrum confirms that the peaks belong to the same molecule, and the 2D {^1^H} grEXSY spectra reveals a very fast exchange between the free and ligated U^VI^O_2_^2+^ GT arms of **3a** (Figure S13).

**Figure 5:**
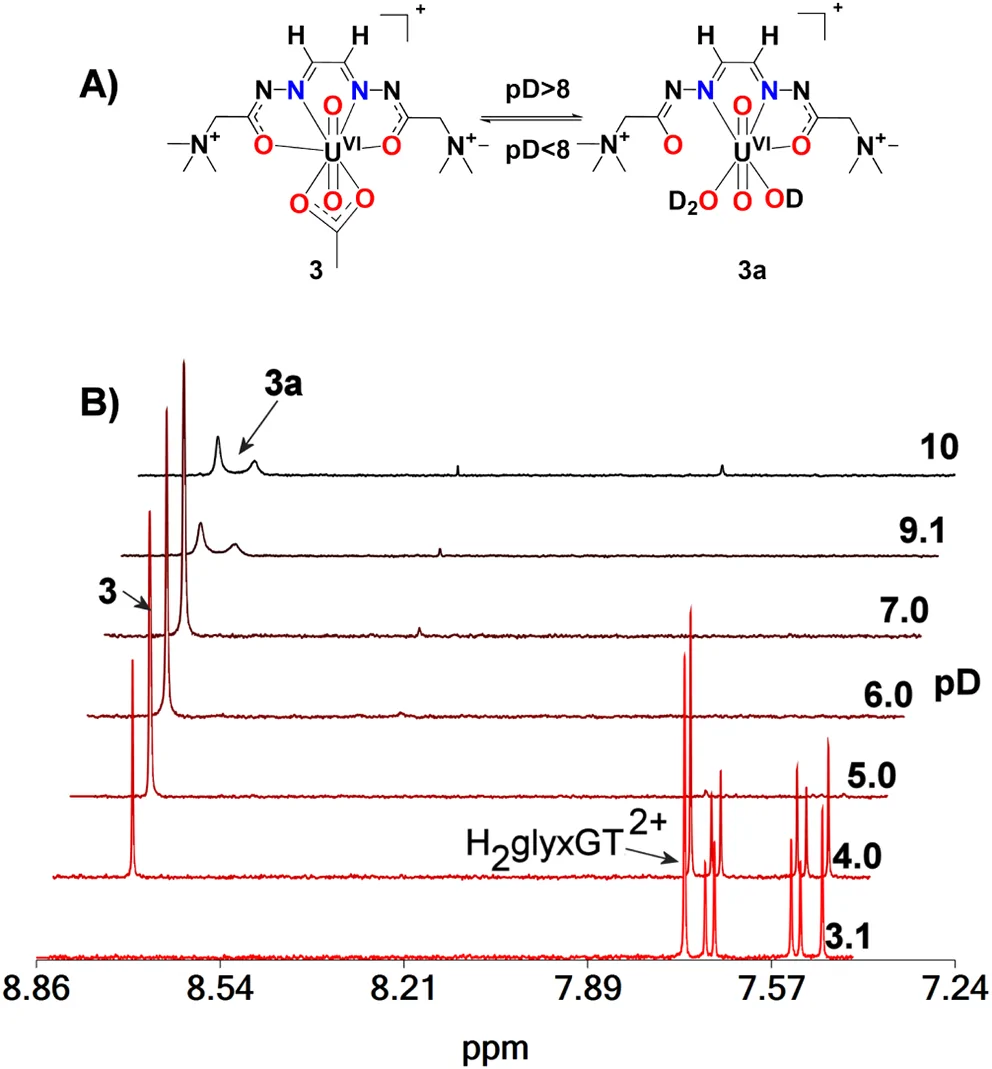
(A) Hydrolysis of **3** at pDs > 8 and formation of **3a**, (B) ^1^H NMR spectra (glyoxal protons) of aqueous solution of complex **3** (4.0 mM) at different pDs.

#### ^1^H and ^51^V NMR Investigation of the Speciation of H_2_dapGT^2+^/H_2_glyxGT^2+^ −V^V^O_2_^+^ in D_2_O at Various pDs

The ^51^V NMR spectra of aqueous solutions of equivalent quantities of H_2_dapGT^2+^/H_2_glyxGT^2+^ and vanadates showed broad peaks at acidic pDs assigned to complexes of V^V^O_2_^+^ with the ligands. At pDs higher than 7.0, the spectra showed the formation of inorganic vanadate oligomers originating from the hydrolysis of the (V^V^O_2_^+^)–H_2_dapGT^2+^/H_2_glyxGT^2+^ complexes. The ^1^H NMR gave multiple overlapping signals, suggesting the formation of many species in solution that were impossible to characterize (Figure S14). The vanadium ion probably cannot utilize all the donor atoms offered by the H_2_dapGT^2+^ and H_2_glyxGT^2+^ ligands, leading to asymmetric or higher-nuclearity molecules, making characterization with ^1^H NMR extremely challenging. ESI-MS was further examine the speciation of vanadium with H_2_dapGT^2+^ and H_2_glyxGT^2+^ ligands in aqueous solution. Experiments were conducted using ESI-MS analysis at three pH levels (3, 7, and 10). The ESI-MS results indicated the formation of mononuclear and multinuclear species interacting with the ligands, which accounts for the multiple species observed by NMR (Figures S15 and S16).

### UV-Vis Investigation of the Hydrolytic Stability of Complexes **1**, **3**, and **4**

The UV-vis spectra of the aqueous solutions of H_2_dapGT^2+^, H_2_glyxGT^2+^, **1** and **3** at pH 7.0 are depicted in Figure S17. The stabilities of **1** and **3** in H_2_O at various pH values were investigated using UV-vis spectroscopy (Figure 6). Although the concentrations in the UV-vis experiments were more than 60 times lower than those in the respective ^1^H NMR experiments, the relationship between hydrolytic stability *vs* pH remained consistent. The UV-vis spectra of the aqueous solution of **1** remained the same in the pH range 3–10. An increase in pH resulted only in a slight enhancement of the intensity of the peaks at λ_max_ = 290 and 375 nm, presumably due to the replacement of the equatorial H_2_O ligated on U^VI^O_2_^2+^ with OH^–^ at higher pH values.

**Figure 6:**
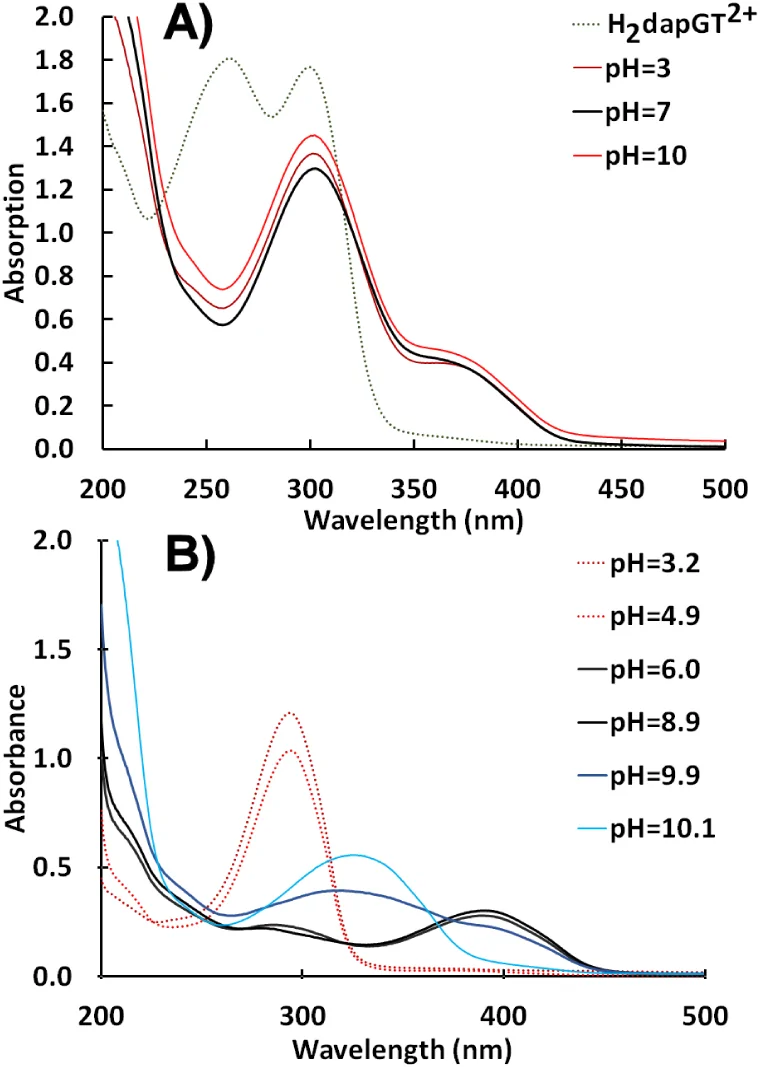
UV-vis spectra of the aqueous solution of (A) H_2_dapGT^2+^ (0.010 mM, pH = 7.0) and complex **1** (0.070 mM), (B) complex **3** (0.034 mM), at various pH values.

The UV-vis spectra of the aqueous solutions of **3** at pH below 5 showed a peak at λ_max_ = 295 nm was assigned to the free ligand. Upon increasing the pH, a new peak appeared at λ_max_ = 394 nm, which was assigned to the formation of **3**. At pH values above 8, the peak at λ_max_ = 394 nm was replaced by the peak at λ_max_ = 326 nm, which was assigned to the formation of [U^VI^(O)_2_(*κ* ^3^-glyxGT)­(H_2_O)­(OH)]^+^ or [U^VI^(O)_2_(*κ* ^3^-glyxGT)­(OH)_2_].

In order to ascertain the nature of the band appearing at high pH in the UV-vis spectrum and understand the electronic structure and nature of the transitions (Figure S18) in complex **3**, density functional calculations were carried out. Based on the analysis of **3**, the simulated band in the visible region is situated at 414 nm (Figure S19), corresponding to the spin-orbit coupled state 65 (Figure S20). Its nature is mostly due to ligand-to-metal charge transfer (LMCT) stemming from singlet 11 combined with singlet 18, triplet 22, and a collection of other states contributing under 4% (Table S5). The two signed difference densities for SOC state 65 are shown in Figure S20. The experimental spectrum records a band at 394 nm, which shows excellent agreement with the TDDFT-simulated spectrum. To identify the species giving rise to the absorption band around 326 nm, a number of possible candidates were evaluated (Figures S21–S25). ESI-MS and NMR data indicate that the ligand is under-coordinated in solution, adopting *κ* ^3^. This does have an acceptable deviation from the experimental value (356 nm vs 326 nm), as shown in Figure S26.

Upon increasing the pH of an aqueous solution of **4**, the UV-vis spectra revealed a shift in the intraligand peak from λ_max_ = 282 to 301 nm. Concurrently, the LMCT transfer shoulder exhibited a slight red shift of approximately 10 nm (Figure S27), attributable to the formation of the [Fe^III^(dapGT)­(OH)]^2+^ species supported and from the potentiometric studies, *vide infra*. Consequently, dimerization of [Fe^III^(dapGT)­(OH)]^2+^ results in the formation of **5**, which was characterized in solid state.

The speciation of complexes **1**–**5** in aqueous solutions was also examined using ESI-MS spectroscopy, which yielded results consistent with those obtained from UV-vis and NMR spectroscopies (Figures S28 and S29).

### Potentiometric Speciation and Stability Constants of U^VI^O_2_^2+^ and Fe^III^ Complexes with H_2_dapGT^2+^ and H_2_glyxGT^2+^ Ligands

H^+^-ISE (Hydrogen-Ion Selective Electrode) potentiometric titrations were carried out on ligand and metal/ligand mixture to define both the protonation constants of H_2_dapGT^2+^ and H_2_glyxGT^2+^ and complexes stability constants. The protonation data of the ligands H_2_dapGT^2+^ and H_2_glyxGT^2+^, considering three and two protonation sites, respectively, are summarized in Table 2. Figures S30 and S31 show speciation diagrams of the ligand solutions. For H_2_dapGT^2+^ 895 titration points were between pH 1.7 and 12.0, whereas for H_2_glyxGT^2+^, given the mild acidity of its protogenic groups, it was not necessary to work with a wider pH range. In this case, 237 titration points between pH 3.0 and 11.0 were considered.

**Table 2:** H_2_dapGT^2+^ and H_2_glyxGT^2+^ Protonation Constants (Reported as Global log β and Stepwise log *K* Values)^a^

*q*, *s*	Protic site	Species	log β	log *K*
***q*dapGT + *s*H^+^ ⇄ (dapGT)_*q*_(H^+^)_*s*_**				
1, 1	NCO^–^	HdapGT^+^	10.19 ± 0.02	10.19 ± 0.02
1, 2	NCO^–^	H_2_dapGT^2+^	19.02 ± 0.02	8.83 ± 0.03
1, 3	N_pyr_	H_3_dapGT^3+^	21.31 ± 0.03	2.29 ± 0.04
***q*glyxGT +*s*H^+^ ⇄ (glyxGT)_*q*_(H^+^)_*s*_**				
1, 1	NCO^–^	HglyxGT^+^	9.00 ± 0.02	9.00 ± 0.02
1, 2	NCO^–^	H_2_glyxGT^2+^	16.78 ± 0.02	7.78 ± 0.03

a Reported value are experimentally determined at *I* = 0.1 mol·L^–1^ and *T* = 25 °C.

Speciation of U^VI^O_2_^2+^ and Fe^III^ in the presence of H_2_dapGT or H_2_glyxGT was assessed in 0.1 mol·L^–1^ NaCl_(aq)_ solution at 25 °C. The H^+^-ISE potentiometric titrations were performed on metal ion and H_2_dapGT·Cl_2_ or H_2_glyxGT·Cl_2_ mixtures (see Experimental Details in the Supporting Information). The collected data were treated by BSTAC software,^102^ to optimize the complex stability constants, taking into account H_2_dapGT and H_2_glyxGT protonation, U^VI^O_2_^2+^ and Fe^III^ hydrolysis, and U^VI^O_2_^2+^/ Cl^–^ and Fe^III^/Cl^–^ interactions (Tables 2 and S7). Acetic acid (Hac) protonation and interactions with U^VI^O_2_^2+^ ions were also considered (Table S7). Moreover, when computing the speciation diagram, the solid hydrolytic species [UO_2_(OH)_2_]_(s)_ were considered in the speciation model (Table S8). The speciation diagrams were computed using PyES.^103^ The species and the corresponding stability constants formed by U^VI^O_2_^2+^ and Fe^III^ with dapGT and glyxGT are listed in Tables 3 and S9.

**Table 3:** U^VI^O_2_^2+^/dapGT, U^VI^O_2_^2+^/glyxGT and Fe^III^/dapGT Complex Formation Constants; Reported Values Are Experimentally Determined at *I* = 0.1 mol·L^–1^ and *T* = 25 °C

*p*, *q*, *r*, *s*	species	log β ± sd^a^
***p*U^VI^O_2_^2+^ + *q*dapGT + *s*H^+^ ⇄ (U^VI^O_2_^2+^)_*p*_(dapGT)_*q*_(H^+^)_*s*_**		
1, 1, 0	[U^VI^O_2_(dapGT)]^2+^	17.57 ± 0.06
1, 1, –1	[U^VI^O_2_(dapGT)(OH)]^+^	7.1 ± 0.1
***p*U^VI^O_2_^2+^ + *q*glyxGT + *r*ac^–^ + *s*H^+^ ⇄ (U^VI^O_2_^2+^)_*p*_(glyxGT)_*q*_(ac^–^)_*r*_(H^+^)_*s*_**		
1, 1, 0, 0	[U^VI^O_2_(glyxGT)]^2+^	11.16 ± 0.08
1, 1, 1, 0	[U^VI^O_2_(glyxGT)(ac)]^+^	14.96 ± 0.02
1, 1, –1, 0	[U^VI^O_2_(glyxGT)(OH)]^+^	5.61 ± 0.03
***p*Fe^III^ + *q*dapGT + *s*H^+^ ⇄ (Fe^III^)_*p*_(dapGT)_*q*_(H^+^)_*s*_**		
1, 1, 0	[Fe^III^(dapGT)]^3+^	19.57 ± 0.05
1, 1, –1	[Fe^III^(dapGT)(OH)]^2+^	14.20 ± 0.05
1, 1, −2	[Fe^III^(dapGT)(OH)_2_]^+^	4.76 ± 0.05

a Standard deviation.

#### (U^VI^O_2_^2+^)–dapGT

Potentiometric curves of (U^VI^O_2_^2+^)–dapGT mixtures indicate the formation of a mono-chelated uranyl complex [U^VI^O_2_(dapGT)]^2+^ at acidic pH. The species is also stable at neutral and mildly alkaline pH. This observation is in agreement with the findings on hydrolytic stability of species 1, reported in the previous paragraphs. In both 1:1 and 1:2 (U^VI^O_2_^2+^)–H_2_dapGT^2+^ aqueous mixtures, [U^VI^O_2_(dapGT)]^2+^ represent the most relevant species in solution in the investigated pH range (as depicted in Figure 7). This finding was in accordance with the UV spectra shown in Figure 6.

**Figure 7:**
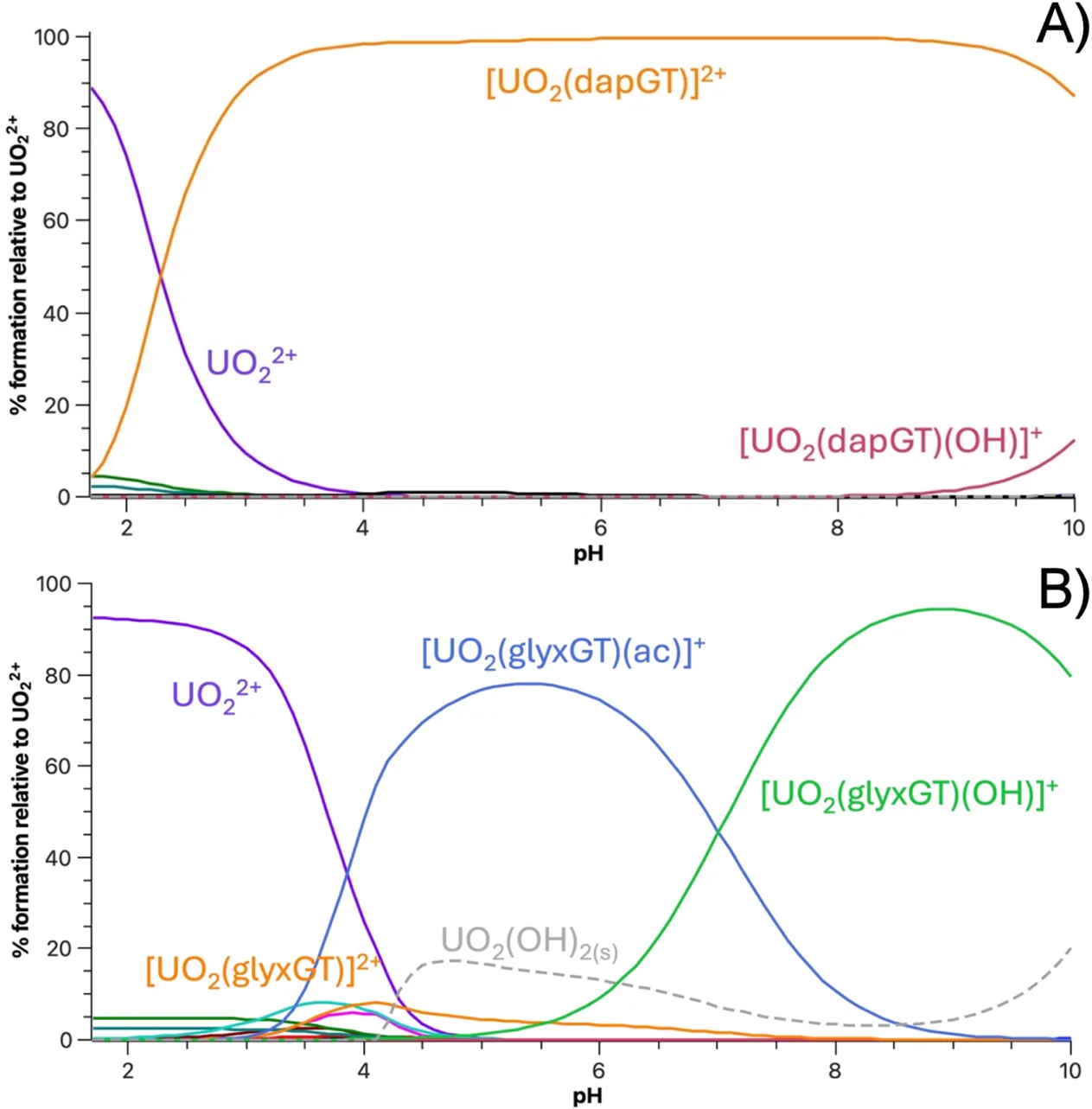
Species distribution for (A) U^VI^O_2_^2+^/dapGT aqueous system ( $c_{UO_{2}^{2+}}=c_{dapGT}$ = 3 mmol·L^*–*1^) and B) U^VI^O_2_^2+^/glyxGT aqueous system ( $c_{UO_{2}^{2+}}=c_{glyxGT}$ = 3 mmol·L^–1^). I = 0.1 mol·L^–1^ in NaCl_(aq)_, T = 25 °C.

#### (U^VI^O_2_^2+^)–glyxGT

In (U^VI^O_2_^2+^)–glyxGT mixtures, mono-chelated uranyl species were formed at low percentages at acidic pH. Moreover, the formation of a mixed acetate-glyxGT uranyl species [U^VI^O_2_(glyxGT)­(ac)]^+^ (complex **3**) was suggested by fitting potentiometric data. This species predominates in solution under mildly acidic conditions. Between 7 < pH < 8, hydrolytic complex [U^VI^O_2_(glyxGT)­(OH)]^+^ is formed. The formation of solid [UO_2_(OH)_2_]_(s)_ was experimentally observed for a 1:1 (U^VI^O_2_^2+^)–H_2_glyxGT^2+^ aqueous mixture, while no solid species were observed for a higher ligand to metal ratio (U^VI^O_2_^2+^)–H_2_glyxGT^2+^ aqueous mixture. Species distribution was computed for the latter two cases, and speciation diagrams are shown in Figure 7.

#### (Fe^III^)–dapGT

Potentiometric titrations show that the dapGT ligand undergoes complete deprotonation even under strongly acidic conditions in the presence of Fe^III^, suggesting the formation of a stable [Fe^III^(dapGT)]^3+^ complex. Furthermore, at pH > 7, the subsequent release of two H^+^ ions, suggests the formation of hydrolytic complex species, namely [Fe^III^(dapGT)­(OH)]^2+^ and [Fe^III^(dapGT)­(OH)_2_]^+^. No precipitation was observed during the experiment. The speciation diagram of the 1:1 (Fe^III^)–H_2_glyxGT^2+^ mixture is shown in Figure S32.

#### (Fe^III^)–glyxGT

During the potentiometric titration of (Fe^III^)–H_2_glyxGT^2+^ solutions precipitation occurred in the range 2 ≤ pH ≤ 3. Increasing the pH the precipitate did not result in re-dissolution. Notably, deprotonation of H_2_glyxGT^2+^ was observed in the same pH range as in the absence of metal cations. Analysis of the titration curves suggests that precipitation as [Fe­(OH)_3_]_(s)_ occurs at an acidic pH, whereas H_2_glyxGT^2+^ behaves as a simple weak acid in solution. These observations suggest that no (Fe^III^)–glyxGT metal complexes are formed in solution under the investigated conditions.

### Assessing the Stability and Selectivity of (U^VI^O_2_^2+^)–dapGT/glyxGT via NMR Studies with Competing Metal Ions and Ligands

Stability Assessment of Uranyl–dapGT/glyxGT Complexes in the Presence of H_2_dipic, H_3_pidiox, H_2_bihyat, and CO_3_^2–^ Using ^1^H and ^13^C­{^1^H} NMR Spectroscopy. The stability of the (U^VI^O_2_^2+^)– dapGT/glyxGT complexes was assessed in the presence of H_2_dipic, H_3_pidiox, H_2_bihyat, or CO_3_^2–^. H_2_dipic was selected because of its high potency to bind uranyl ions. The ligand H_2_bihyat exhibits exceptional binding strength for both metal ions, surpassing even amidoximes and aminocarboxylate ligands.^13,104^ The CO_3_^2–^ ion serves as a significant ligand for uranium and constitutes the primary marine uranyl species, [U^VI^O_2_(CO_3_)_3_]^4–^. The introduction of H_2_dipic, H_2_bihyat, or CO_3_^2–^ into the D_2_O solutions of **3** led to the liberation of free H_2_glyxGT^2+^ in solution, thereby corroborating the hydrolytic instability of **3** as observed in^1^H NMR versus pD experiments (Figure 5) and the potentiometric studies (Figure 7, Table 3). Comparison of log*β* values with other tetradentate ligands indicates that H_2_glyxGT_2_^2+^ exhibits greater ligand binding strength for uranyl than other flexible carboxylate ligands such as H_2_EDDA.^85^ However, it demonstrates lower stability compared to preorganized carboxylate ligands, specifically 2,2-bipyridine-6,6-dicarboxylate (BiPDA) and 1,10-phenanthroline-2,9-dicarboxylate­(PhenDA).^86^

The ^1^H NMR spectra of a solution of **1** (4.0 mM in D_2_O) in neither the presence or absence of H_2_dipic or H_3_pidiox, did not show any difference at pDs 7.0, and 9.0, even with very high excess (up to [H_3_pidiox]/[**1**] = 8, [H_2_dipic]/[**1**] **=**16) (Figure S33). The high hydrolytic stability of the aqueous solution of **1** (4.0 mM) was further demonstrated by the addition of an excess of ^13^CO_3_^2–^ (up to [Na_2_^13^CO_3_]/[**1**] = 50) at pDs 7.0 and 9.0. The ^13^C­{^1^H} NMR spectra of **1** and ^13^CO_3_^2–^ at pD 7.0 exhibited only the peaks corresponding to ^13^CO_3_^2–^ and H^13^CO_3_^–^ at 160 and 124 ppm,^105,106^ respectively, indicating that **1** remained intact in solution. At high pD (9.0) in the presence of high concentrations of ^13^CO_3_^2–^ ([^13^CO_3_^2–^] = 50 × [dapGT]), a peak appeared at 168 ppm, attributed to U^VI^O_2_^2+^–^13^CO_3_^2–^ salts, revealing partial hydrolysis of **1** (Figure S34). In contrast to H_2_dipic, H_3_pidiox, and CO_3_^2–^, the introduction of H_2_bihyat into the aqueous solutions of **1** at pDs 7.0 and 9.0, led to the formation of U^VI^O_2_^2+^–bihyat^2–^ species, with the concurrent release of H_2_dapGT^2+^ into the solution (Figure S35). This observation confirms the high binding affinity of BHT-type ligands for the uranyl moiety.^13,39,59^ The comparative binding affinity of bihyat^2–^ versus dapGT was illustrated through the equilibrium constants derived from ^1^H NMR calculations (Figure 8). The equilibrium constant is expressed as in eq 2 and refers to equilibrium reported in eq 1 The value of *K*_pD7_ = 0.023 ± 0.002 is notably smaller than *K*_pD9_ = 5.5 ± 0.4, indicating that an increase in pD shifts the equilibrium toward the formation of [U^VI^(O)_2_(bihyat)­(H_2_O)] rather than [U^VI^(O)_2_(dapGT)­(H_2_O)].

1 $$\left[U^{\mathrm{VI}}O_{2}\left(\mathrm{dapGT}\right)\left(H_{2}O\right)]+H_{2}\mathrm{bihyat}⇄[U^{\mathrm{VI}}O_{2}\left(\mathrm{bihyat}\right)\left(H_{2}O\right)\right]+H_{2}\mathrm{dapGT}$$

2 $$K=\frac{\left[U^{\mathrm{VI}}O_{2}\left(\mathrm{dapGT}\right)\left(H_{2}O\right)][H_{2}\mathrm{bihyat}\right]}{\left[U^{\mathrm{VI}}O_{2}\left(\mathrm{bihyat}\right)\left(H_{2}O\right)][H_{2}\mathrm{dapGT}\right]}$$

**Figure 8:**
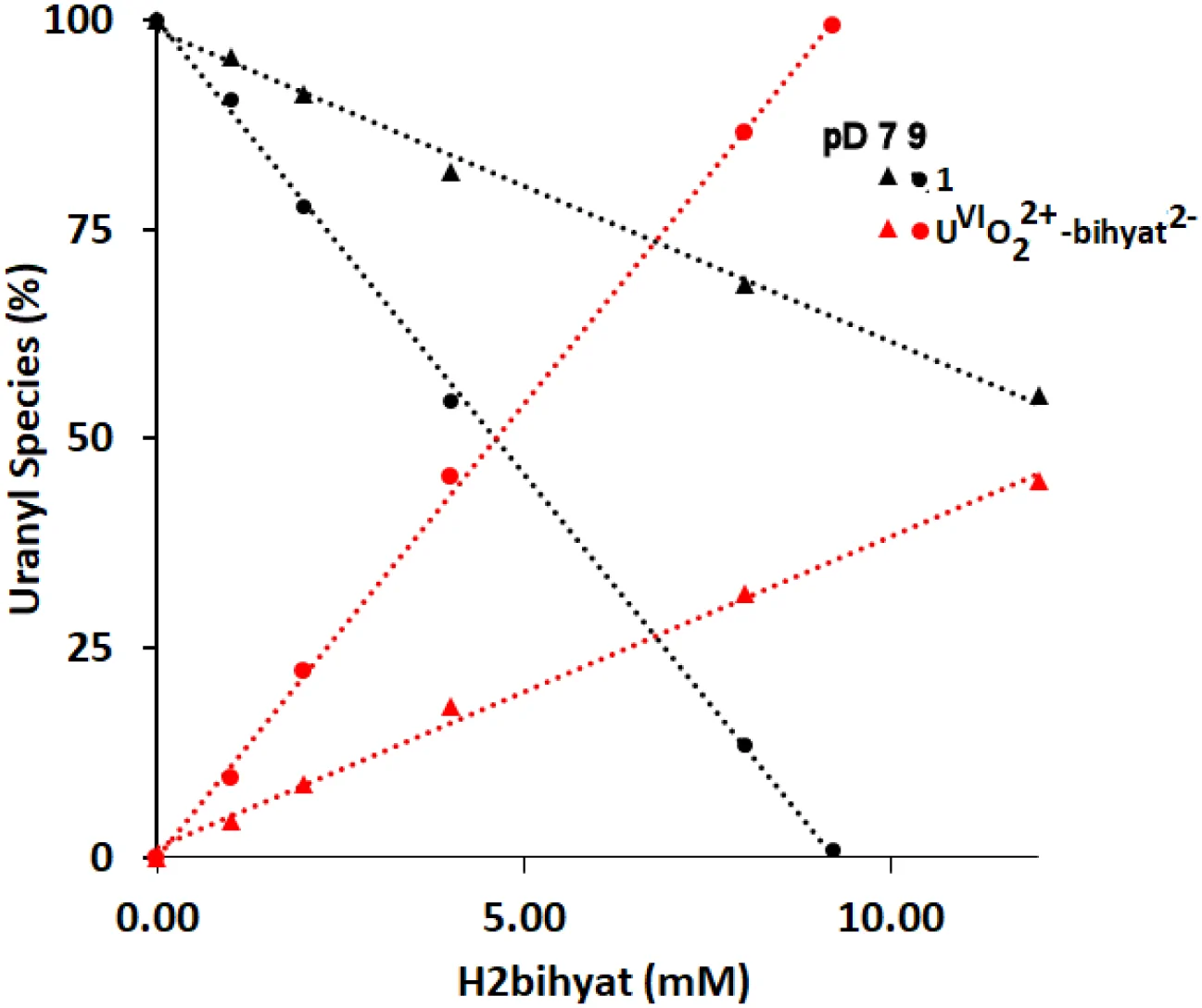
Speciation of **1** (4.0 mM) and U^VI^O_2_^2+^–bihyat^2–^ versus added H_2_bihyat concentration at pDs 7.0 and 9.0.

### Selective Binding and Stability of (U^VI^O_2_^2+^)–dapGT/glyxGT Complexes with Vanadate Ions

The introduction of an aqueous solution of VO_4_^3–^ (5.0 mM) to a solution containing U^VI^O_2_^2+^ (2.5 mM) and H_2_dapGT^2+^ (2.5 mM), or alternatively, the addition of U^VI^O_2_^2+^ (2.5 mM) to a solution of V^V^O_4_^3–^ (5.0 mM) and H_2_dapGT^2+^ (2.5 mM) at pH levels of 7.0 and 9.0, resulted in the precipitation of compound **2**. The ^1^H NMR spectra of the solution exhibited peaks solely from complex **1**, whereas the ^51^V NMR spectra revealed peaks corresponding to the vanadate oligomers (Figure S36). Therefore, in the presence of U^VI^O_2_^2+^, V^V^O_4_^3–^, dapGT selectively binds only to U^VI^O_2_^2+^.

While multidentate ligands such as BiPDA and PhenDA can establish strong complexes akin to dapGT, they exhibit significantly lower selectivity for binding to U^VI^O_2_^2+^.^37^ The enhanced selectivity of dapGT in binding U^VI^O_2_^2+^ compared to BiPDA and PhenDA is attributed to the strong coordination interaction of aminocarboxylate ligands with V^V^O_2_^+^. Carboxylates are more versatile ligating groups, capable of forming complexes with various coordination modes, compared with the carbohydrazide group. Although carboxylates can form stable complexes independently, the carbohydrazide moiety requires the formation of chelate rings including other donor atoms in order to stabilize its complexes. Thus, if dapGT does not ligate the metal in a pentadentate mode, it cannot form stable complexes with metal ions. U^VI^O_2_^2+^, in contrast to V^V^O_2_^+^, is able to form a multicoordinated complex with the dapGT ligand resulting in high binding selectivity. While BiPDA and PhenDA are also multidentate ligands similar to dapGT, their reduced selectivity can be attributed to the strong interactions of the carboxylate groups with V^V^O_2_^+^.^37^

The addition of an aqueous solution of VO_4_^3–^ (5.0 mM) to an aqueous solution of compound **3** (2.5 mM) resulted in the precipitation of a yellow solid, which was identified as a heteropolyoxo U^VI^O_2_(V^V^O_3_)_2_ mixed salt.^13^ The ^1^H NMR spectra of the solution only displayed signals corresponding to the tautomers of free H_2_glyxGT^2+^. This observation suggests that the high stability of the mixed U^VI^O_2_(V^V^O_3_)_2_ salt drives the reaction toward the decomposition of compound **3**, thereby confirming the low affinity of H_2_glyx GT^2+^ for binding U^VI^O_2_^2+^.

## Conclusions

In this study, we have demonstrated that planar, multidentate Girard-T-based acylhydrazone ligands can achieve highly selective binding of UO_2_^2+^ in aqueous media through enforced multidentate coordination within the uranyl equatorial coordination plane. A family of U^VI^O_2_^2+^ and Fe^III^ complexes with novel pentadentate (dapGT) and tetradentate (glyxGT) ligands were synthesized and subjected to structural and physicochemical characterization. The pentadentate dapGT ligand forms exceptionally stable complexes with uranyl ions and, under competitive conditions, effectively prevents coordination with vanadium ions. This reflects an intrinsic selectivity driven by geometric and denticity factors, without lacking binding capacity. This selectivity of dapGT to bind U^VI^O_2_^2+^and V^V^O_2_^+^ is the highest reported to date. In contrast, the more flexible tetradentate glyxGT ligand exhibits reduced stability but retains preferential uranyl binding. The lower stability constants of glyxGT for binding U^VI^O_2_^2+^, compared to other tetradentate preorganized rigid ligands reported in the literature,^60^ may be attributed to the considerable flexibility of glyxGT. Ongoing research involves the synthesis and both solid and solution characterization of more rigid Girard-T-based tetradentate ligands and their uranyl complexes, alongside the elucidation of glyxGT-UO_2_^2+^ hydrolytic instability. Furthermore, cost-effective materials for the extraction of uranium from seawater can be synthesized from acylhydrazone derivatives by substituting the −N­(CH_3_)^3+^ group with active moieties, such as −Cl, which can be attached to polymeric materials through grafting and copolymerization techniques. The instability of Girard-T-based ligands in coordinating uranyl ions at low pH levels may present a method for recycling uranium from materials that have absorbed it at the alkaline pH of seawater. This can be achieved through the acidification of the Girard-T-based absorbing material, offering potential applications in uranium recovery.

Together, these results establish equatorial-plane multidentate coordination by planar multidentate ligands as a robust and generalizable design principle for selective uranyl chelation in water, providing a foundation for the development of next-generation ligands for uranium extraction, waste management, and detoxification applications.

## Supplementary Material



## Data Availability

A dataset collection^107^ of the optimized structures is available in the ioChem-BD repository.^108^

## Notes

Additional experimental details, materials and methods, structural figures, additional ^1^H, ^51^V NMR spectra, DFT UV simulations, ESI-MS spectra, crystallographic data, and Cartesian coordinates of the optimized structures (D) (PDF)
