# Supplementary material for: Water-Soluble Girard T‑Based Multidentate Ligands for Highly Selective Uranyl(VI) Binding
Source: Inorg Chem. 2026 Sep 7;65(37):21867–80. doi: 10.1021/acs.inorgchem.6c03289 (PMC13599386; doi:10.1021/acs.inorgchem.6c03289)
Supplement: Supplementary file 1 [file ic6c03289_si_001.pdf]

## Supporting Information

# Water-Soluble Girard T-Based Multidentate Ligands for Highly Selective Uranyl(VI) Binding

*Angelos Amoiridis,[a,b] Matteo Marafante,[c] Odysseas D. Keramidas, Epameinondas Leontidis,*[a] Silvia Berto,*[c] Nuno A. G. Bandeira,*[d] Haralampos N. Miras,*[e] and Anastasios Keramidas*[a]*

[a] *University of Cyprus, Department of Chemistry, 2109 Nicosia, Cyprus. Email: akeramid@ucy.ac.cy*

[b] *Department of Health Sciences, School of Life & Health Sciences, University of Nicosia, 2417 Nicosia, Cyprus*

[c] *Dipartimento di Chimica, Università di Torino, Via P. Giuria 7, 10125, Torino, Italy Email: silvia.berto@unito.it*

[d] *Biosystems and Integrative Sciences Institute (BioISI)- Departamento de Química e Bioquímica. Faculdade de Ciências Universidade de Lisboa; 8.5.53 - C8 Campo Grande, 1749-016 Lisboa Portugal. E-mail: nuno.bandeira@ciencias.ulisboa.pt*

[e] *School of Chemistry, The University of Glasgow. Glasgow G12 8QQ (UK). Email: Charalampos.moiras@glasgow.ac.uk*

**Contents** **page**

Experimental S4

**Figure S1**. ORTEP plots of **1′** with 50% thermal ellipsoids. Counter anions have been omitted for clarity. S7

**Figure S2**. IR Spectra of (A) $H_2dapGT^{2+}$, **1** and (B) $H_2glyxGT^{2+}$, **3**. S8

**Figure S3.** ESI-MS of methanol solutions of $H_2dapGT^{2+}$ and $H_2glyxGT^{2+}$. S9

**Figure S4**. $^1H$ NMR of the aqueous solution of $H_2glyxGT^{2+}$ pD = 7.0. S10

**Figure S5**. $^1H$ NMR of the aqueous solution of $H_2dapGT^{2+}$ (4.00 mM) pD = 7.0. S10

**Figure S6**. 2D {$^1H$,$^{13}C$} grHSQC NMR (aromatic region) of the aqueous solution of $H_2dapGT^{2+}$ at pD = 7.0. S11

**Figure S7**. 2D {$^1H$} grCOSY NMR of the aqueous solution of $H_2glyxGT^{2+}$ (4.00 mM) pD = 5.3. S12

**Figure S8**. 2D {$^1H$} grCOSY NMR (aromatic region) of the aqueous solution of $H_2dapGT^{2+}$ (4.00 mM) at pD = 7.0. S12

**Figure S9.** $^1H$ NMR of the aqueous solution of $H_2glyxGT^{2+}$ (4.00 mM) *vs* pH. S13

**Figure S10.** 2D {$^1H$} grEXSY NMR of the aqueous solution of $H_2glyxGT^{2+}$ (4.00 mM) at pD = 7.0, assignments and exchange mechanism. S13

**Figure S11**. $^1H$ NMR of the aqueous solution of **1** (4.00 mM) and **3** (4.00 mM) pD = 7.0. S14

**Figure S12**. $^1H$ NMR spectra of aqueous solution (4.00 mM) of complex **1** at different pDs. S14

**Figure S13**. A) 2D {$^1H$} grCOSY NMR spectra B) 2D {$^1H$} grEXSY NMR spectra C) exchange mechanism, of a $D_2O$ solution of complex **1** (4.00 mM) at pD=10.5. S15

**Figure S14**. A) $^1H$ NMR and B) $^{51}V$ NMR for solutions of $H_2dapGT^{2+}$ (4.00 mM) and $NaVO_3$ (4.00 mM) at pD 4, 7 and 9. S15

**Figure S15**. ESI-MS of the reaction mixture of $NaVO_3$ (4 mM) and the ligand $H_2dapGT^{2+}$ (2 mM) in $H_2O$:MeOH at a) pH = 3 and b) pH = 9. S16

**Figure S16.** ESI-MS of the reaction mixture of $NaVO_3$ (4 mM) and the ligand $H_2glyxGT^{2+}$ (2 mM) in $H_2O$:MeOH at A) pH = 3 and B) pH = 9. S16

**Figure S17.** UV-Vis spectra (aqueous solution, pH=7) of a) $H_2dapGT^{2+}$ and **1** and b) $H_2glyxGT^{2+}$ and **3**. S18

**Figure S18.** Kohn-Sham orbitals and ligand field splitting of the 5f orbitals in a hexagonal bipyramid environment. S18

**Figure S19.** Calculated UV-Vis spectrum of complex **3**. S19

**Figure S20.** Natural difference density of SOC state 65 in complex 3 at 0.0005 a.u. isocontour. Purple represents electron depletion, yellow electron gain. S19

**Figure S21.** Calculated UV-Vis spectrum of $glyxGT^{2+}$. S19

**Figure S22**. Calculated absorption spectrum of the $[(U^{VI}O_2)glyxGT(\mu\text{-}OH)]_2^{2+}$ complex.

S20

**Figure S23**. Calculated UV-Vis spectrum of the optimised dioxo (top) and monoxo (bottom) bridged dimers. S20

**Figure S24.** Calculated UV-Vis spectrum of $[(U^{VI}O_2)(OH)_2(\kappa^4\text{-glyxGT})]$. S21

**Figure S25.** Transition difference densities of the most intense spinor states (108, 90 and 89 see Table 1) at 0.0005 a.u. isocontour. Purple represents electron depletion, yellow electron gain. S21

**Figure S26**. Calculated UV-vis spectrum of $[(U^{VI}O_2)(OH)_2(\kappa^3\text{-glyxGT})]$ S22

**Figure S27.** UV-Vis Spectra of the aqueous solution of complexes **4** (0.070 mM) and **5** (0.070 mM), at various pH values. S24

**Figure S28**. ESI-MS of complexes a) **1** and b) **3** in $H_2O$:MeOH medium. S25

**Figure S29.** ESI-MS of complexes a) **4** and b) **5** in $H_2O$:MeOH medium. S26

**Figure S30.** Species distribution for dapGT protonation ($c_{dapGT}$ = 3 $mM$, $I$ = 0.1 $M$ in $NaCl_{(aq)}$, $T$ = 25 °C) S27

**Figure S31**. Species distribution for glyxGT protonation ($c_{glyxGT}$ = 3 $mM$, $I$ = 0.1 $mM$ in $NaCl_{(aq)}$, $T$ = 25 °C) S27

**Figure S32.** Species distribution for $Fe^{III}$/dapGT aqueous system ($c_{Fe^{III}} = c_{dapGT}$ = 3 mM, $I$ = 0.1 M in $NaCl_{(aq)}$, $T$ = 25 °C). S28

**Figure S33**. $^{1}H$ NMR titration spectra of a solution of **1** (4.00 mM) with (A) $H_3$pidiox (1.00–10.0 mM) at pD=9 and (B) $H_2$dipic (1.00–12.0 mM) at pD=9. The spectra of the same solutions at different pD are identical to the specific ones and were thus omitted from the figure. S29

**Figure S34.** $^{13}C$ NMR Spectra (A) $^{13}CO_3^{2-}$ (200 mM) + **1** (4 mM) at various pDs (B) **1** (4 mM) with increasing concentration of $^{13}CO_3^{2-}$ at pD=9. S30

**Figure S35.** $^{1}H$ NMR titration spectra of a solution of **1** (4 mM) with $H_2$bihyat (1–12 mM). A) pD=7, B) pD=9. ● **1**, ● Free $H_2dapGT^{2+}$, * Ligated $H_2$bihyat, * Free $H_2$bihyat. S31

**Figure S36**. NMR Spectra (A) $^{1}H$ NMR and (B) $^{51}V$ NMR aqueous solution (pD=8) of **1** (2.50 mM) and $V^{V}O_2^{+}$ (5.00 mM) 24 and 48 h after mixing. S32

**Table S1.** Crystallographic parameters for complexes **1**, **1′**, **2** and **3**. S33

**Table S2**. Selected bond lengths (Å) and angles (°) of **1, 1′** and **2**. S36

**Table S3**. Selected bond lengths (Å) and angles (°) of **3**. S37

**Table S4**. Selected bond lengths (Å) and angles (°) of **4** and **5**. S39

**Table S5.** SOC state 65 composition in the basis of scalar states. S42

**Table S6.** Main electronic transitions corresponding to the highest intensity band in

$[U^{VI}O_2(OH)_2$-$\kappa^3$-(glyxGT)]. S42

**Table S7**. $U^{VI}O_2^{2+}$ speciation model. S43

**Table S8**. $[U^{VI}O_2(OH)_2]_{(s)}$ solubility product ($\log K_{ps}$) and related equilibrium.

S43

**Table S9**. $Fe^{III}$ speciation model. S44

**Experimental**

**Materials and Methods.** All chemicals and solvents were purchased from Merck. Microanalyses for C, H, and N were performed using a Euro-Vector EA3000 CHN elemental analyzer. FT-IR transmission spectra of the compounds, were acquired using a Shimadzu IRSpirit model spectrophotometer. The kinetic UV-vis measurements were recorded on a Photonics UV-vis spectrophotometer Model 400, equipped with a CCD array, operating in the range 250 to 1000 nm. The potentiometric titrations were carried out by Metrohm 888 Titrando titrator equipped with a combined $H^+$-ISE ($H^+$-Ion Selective Electrode) Unitrode (Metrohm, model 6.0259.100). Tiamo 2.5 software was used to control the potentiometric experiments. The ligand $H_2$bihyat was synthesized according to the literature. Merck silica gel 60 $F_{254}$ TLC plates were used for thin layer chromatography.

**Single X-ray crystal Structure Analysis.** Single crystal analysis was performed on an Xcalibur Oxford Diffractometer equipped with a Sapphire 3 CCD detector and a 4-cycle Kappa geometry goniometer, using enhanced Mo K$\alpha$ ($\lambda$=0.71073 Å) X-ray source and graphite radiation monochromator. Analytical absorption correction was applied using CrysAlis RED software. CrysAlis CCD and CrysAlis RED software were used for data collection and data reduction/cell refinement respectively. The structure of the compounds was solved by direct methods and refined by full-matrix least-squares techniques on $F^2$ using SHELXS-97.[1, 2] Special computing molecular graphics incorporated in the WinGX 3.2 interface were used. All the non-H atoms were anisotropically refined. The positions of hydrogen atoms in all structures were calculated from stereochemical considerations and kept fixed isotropic during refinement or found in DF map and refined with isotropic thermal parameters.

**NMR and UV-vis Spectroscopies.**

**NMR measurements.** All NMR samples were prepared from crystalline uranium(VI) compounds in $D_2O$ at 25 °C just prior to NMR spectrometric determinations. NMR spectra were recorded on a Bruker Avance III 500 MHz spectrometer. A 30°-pulse width was applied for the $^{1}H$, $^{13}C$ NMR and $^{51}V$ measurements, and 2, 2 and 0.1 s relaxation delay respectively. The samples were prepared from purified molecules at 25 °C just prior to NMR spectrometric measurements. Data acquisition and processing were accomplished using TopSpin 4.0.6 and MultiSpecNMR 5.0.0 (https://sourceforge.net/projects/multispecnmr/). Standard pulse programs as implemented in TopSpin were used for 1D and 2D {$^{1}H$}grCOSY, {$^{1}H$}grEXSY and {$^{1}H$,$^{13}C$}grHSQC data acquisition.

**Determination of commercial $NaVO_3$ purity.** The purity of the commercial $NaVO_3$ was determined as peroxovanadate by UV-Vis spectroscopy. First, the molecular absorptivity ($\varepsilon$) of peroxovanadate was found using 99.95% $V_2O_5$. The determination of $\varepsilon$ was carried out by reacting of $V_2O_5$ with excess of $H_2O_2$ (3 equivalents) in 0.5 M $H_2SO_4$.[3] Various concentrations of this solution were plotted against the absorption at 453 nm. The molecular absorptivity, $\varepsilon$=188.39 $M^{-1}$ $cm^{-1}$ per vanadium, was calculated from eight samples.

$NaVO_3$ reacted with excess of $H_2O_2$ (3 equivalents) in 0.5 M $H_2SO_4$ leading to the characteristic, red-colored solution. The concertation of vanadium in this solution was calculated from the absorbance at 453 nm. The parity of the $NaVO_3$ salt was calculated as the mean value of eight samples containing various amounts of vanadium and found to be 89.42 %.

**Sample Preparation for $^{1}H$ & $^{51}V$ NMR and UV-Vis measurements.**

For the $^{1}H$ and $^{51}V$ NMR measurements the stock solutions (20.0 and 50.0 mM) were prepared by dissolving crystalline materials in $D_2O$. The final solutions were prepared by mixing the appropriate quantities of the stock solution and the pD of the solutions were adjusted by the addition of DCl/NaOD. The values recorded from the pH-meter for $D_2O$ solutions are lower than the pH values by 0.407 units, so this value was added to the pH readings to get the pD values.[4]

**Electrospray Ionization Mass Spectrometry (ESI-MS)**

All MS data were collected using a Q-trap, time-of-flight MS (Maxis Impact MS) instrument supplied by Bruker Daltonics Ltd. The detector was a time-of-flight, micro-channel plate detector and all data was processed using the Bruker Daltonics Data Analysis 4.1 software, whilst simulated isotope patterns were investigated using Bruker Isotope Pattern software and Molecular Weight Calculator 6.45. The calibration solution used was Agilent ES tuning mix solution, Recorder No. G2421A, enabling calibration between approximately 100 *m/z* and 2000 *m/z*. This solution was diluted 60:1 with MeCN. Samples were dissolved in $H_2O$:MeOH solvent mixture and introduced into the MS *via* direct injection at 180 μL $h^{-1}$. The ion polarity for all MS scans recorded was negative, at 453.15 K, with the voltage of the capillary tip set at 4000 V, endplate offset at −500 V, funnel 1 RF at 300 Vpp and funnel 2 RF at 400 Vpp.

**Potentiometry**

The potentiometric titrations were carried out by $H^+$-ISE and were conducted at a constant temperature of $T = 25 \pm 0.1$ °C, under purified $N_2$. The ionic strength was adjusted at $I = 0.1$ M by means of $NaCl_{(aq)}$. Ultra-pure water, produced by a MilliQ system (R = 18.2 MΩ·cm$^{-1}$), was used to prepare all the solutions and samples. The

used electrode was calibrated before each titration for free hydrogen ion concentration, according to the method described by Irving et al.[36] 10 $cm^3$ of samples were titrated using standardized $NaOH_{(aq)}$ solution as titrant. $NaOH_{(aq)}$ and $HCl_{(aq)}$ solutions were prepared by diluting concentrated ampoules (Tritisol®) and standardized with potassium hydrogen phthalate and TRIS (tris(idrossimetil)amminometano), respectively (dried for at least 24 hours. Carbonate-free $NaOH_{(aq)}$ was prepared weekly using freshly boiled ultra-pure water, stored in dark bottles and protected from atmospheric $CO_2$ using soda lime traps. $H_2$dapGT·$Cl_2$ or $H_2$glyxGT·$Cl_2$ were weighted by analytical balance and dissolved in a precise volume of 0.1 M $NaCl_{(aq)}$. Uranyl acetate dihydrate, $UO_2(ac)_2{\cdot}2H_2O$ and $FeCl_3$ stock solutions were prepared, standardized, and utilized as source of $U^{VI}O_2^{2+}$ and $Fe^{III}$, respectively, in the studied solutions. $H^+$-ISE potentiometric titrations were performed on 1.00 mM $\leq c_L \leq$ 3.00 mM $H_2$dapGT·$Cl_2$ or $H_2$glyxGT·$Cl_2$ solutions to define the protonation constants. To study the metal/ligand systems, $U^{VI}O_2^{2+}$ or $Fe^{III}$, and $H_2$dapGT·$Cl_2$ or $H_2$glyxGT·$Cl_2$ mixtures, with metal/ligand concentration ratios 1:1, 1:2 and 1:3, were prepared. The concentrations were ranging between 1.00 mM $\leq c_M \leq$ 3.00 mM, and 1.00 mM $\leq c_L \leq$ 3.00 mM. HCl was added to achieve pH 1.7. The potentiometric data was fitted using BSTAC4 software.[5]

**Computational Details**

Methodology – The ORCA 6.0.1 program code[6-9] was used in all calculations. The ωr2-SCAN range corrected hybrid metaGGA class functional[10-12] was used in the geometry optimisations of each model. The DLU-X2C scalar relativistic Hamiltonian[13-15] was employed to account for relativistic effects. The Gaussian contracted relativistic X2C-SVPall basis set[16] was used for all the lighter elements whereas for uranium the

segmented all electron relativistically contracted SARC-DKH-TZVP[17] basis set was employed. Multi-centre integrals were approximated by the RIJCOSX[18-21] technique with automatically generated auxiliary basis sets.[22] Solvent effects were taken into account by the C-PCM method[23, 24] with the default settings for water.

Time-Dependent density functional runs employed the r2-SCAN[11] functional and kernels without the Tamm-Dancoff[25] approximation (*TDA false* command). Apart from the ligand glyxgt, all models had the inclusion of spin-orbit coupling through mean-field theory (SOMF). [26, 27] The configuration expansion was for 60 roots for the mono-nuclear models and 120 roots for the dinuclear models.

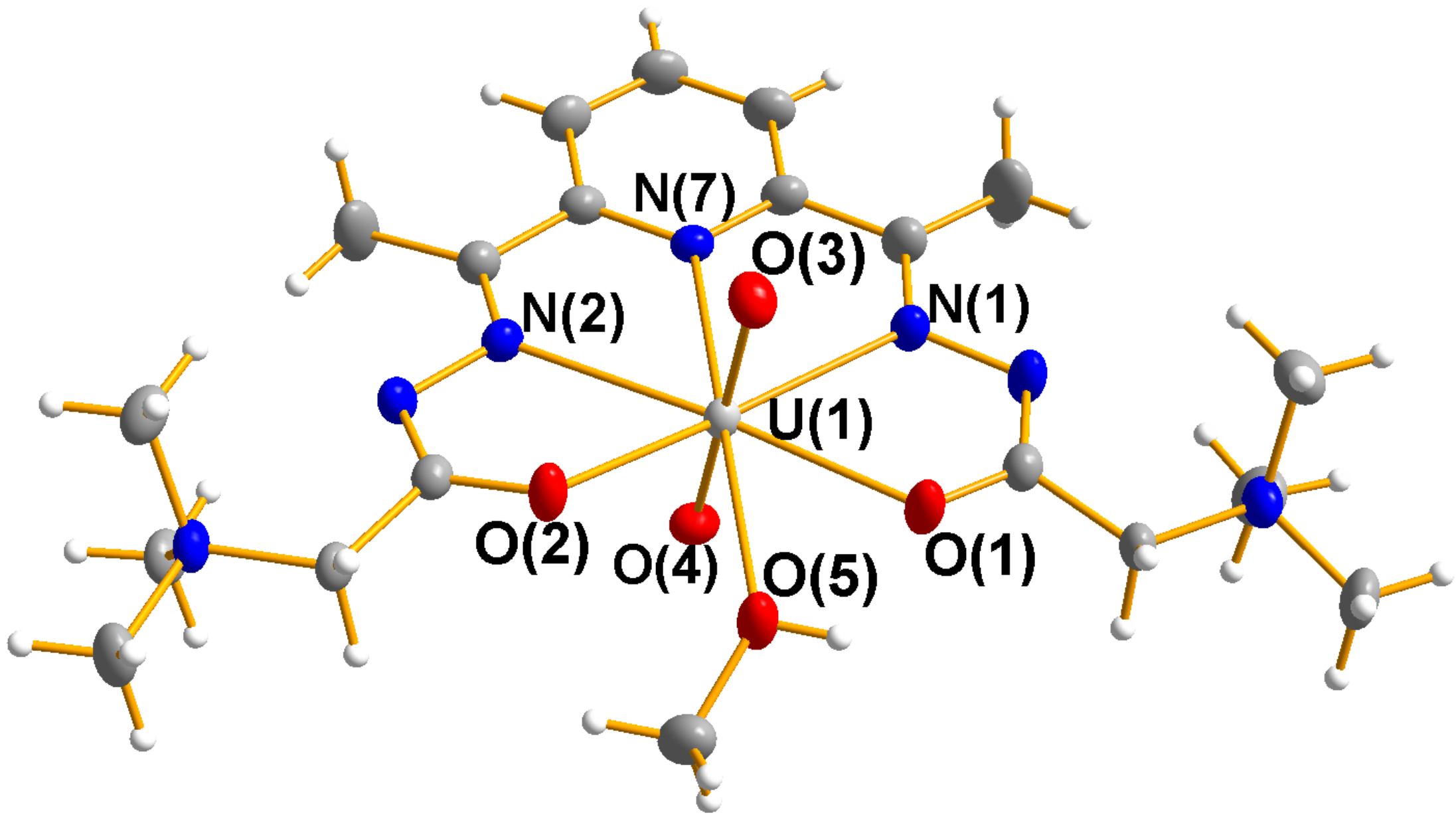


**Figure S1**. ORTEP plots of **1′** with 50% thermal ellipsoids. Counter anions have been omitted for clarity.

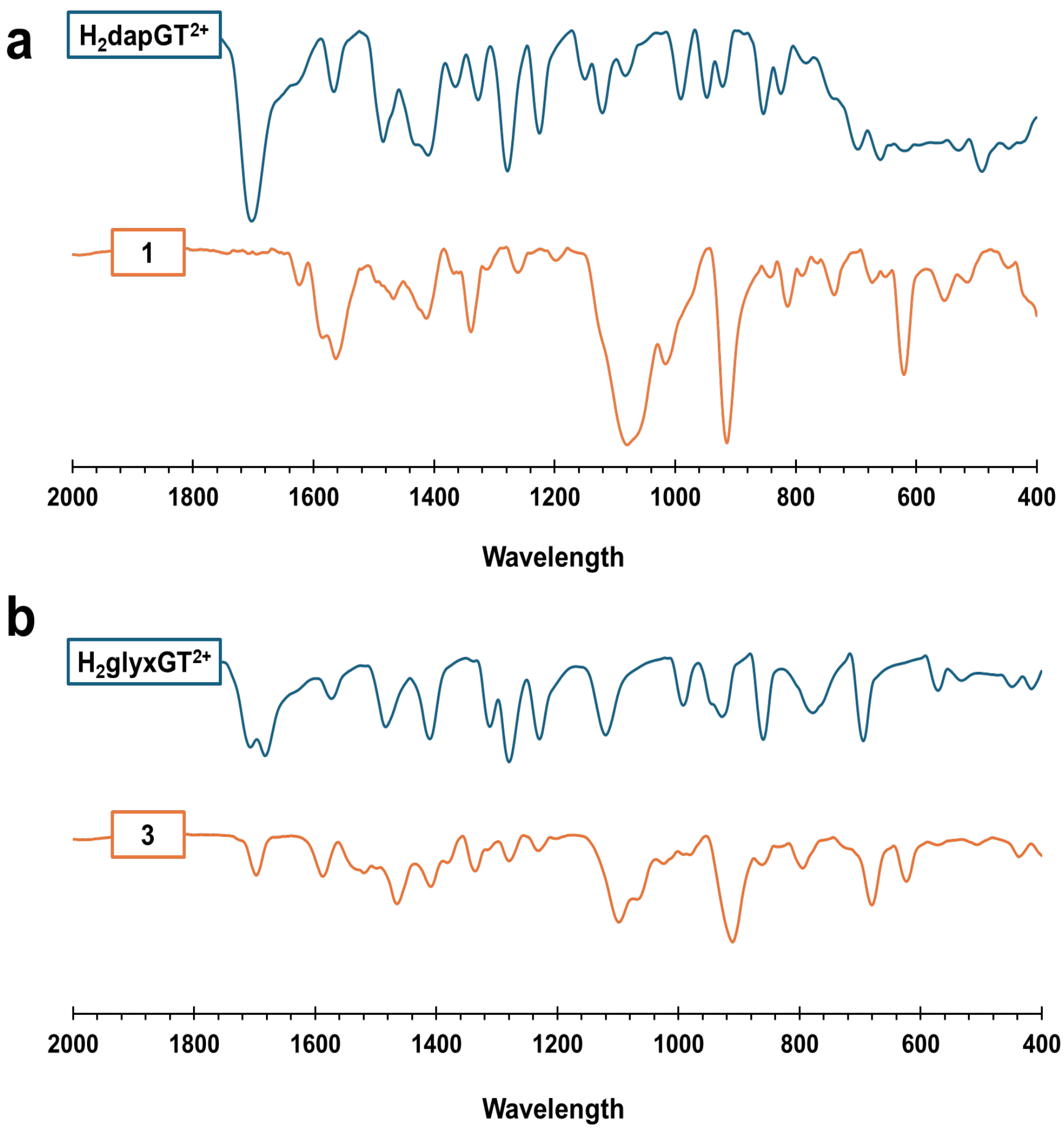


**Figure S2**: IR Spectra of (A) $H_2dapGT^{2+}$, **1** and (B) $H_2glyxGT^{2+}$, **3**.

The ketone-enol character of the acylhydrazone ligands in the complexes was confirmed by IR spectroscopy (Figure S2). The free organic molecules exhibited a pronounced peak at 1700 $cm^{-1}$ [ν(C=O stretching)], which shifted to approximately 1080 $cm^{-1}$ [*ν*(C–O stretching)] upon complexation (IR spectra of **1**–**5**). This shift was attributed to the enolization of the amide carbonyl moiety in the free ligands following their ligation to $U^{VI}O_2^{2+}$ (Scheme 6), which is consistent with the crystallographic characterization of the complexes. The peak at 1700 $cm^{-1}$ in the spectrum of **3** was attributed to the coordinated acetate anion. *ν*(U–O stretching) was observed at 920 $cm^{-1}$ for **1** and at 900 $cm^{-1}$ for **3**.

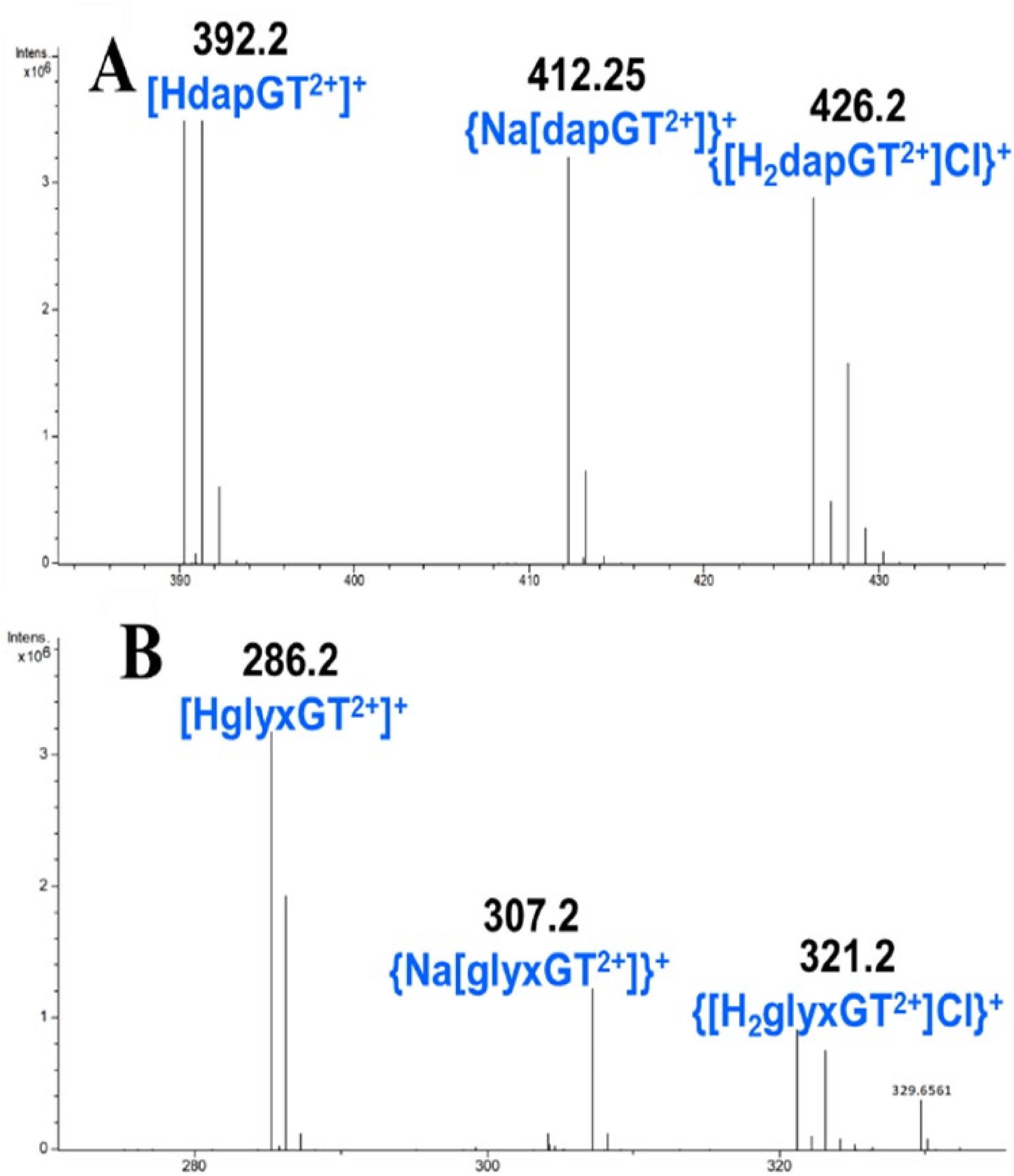


**Figure S3.** ESI-MS of methanol solutions of $H_2dapGT^{2+}$ and $H_2glyxGT^{2+}$.

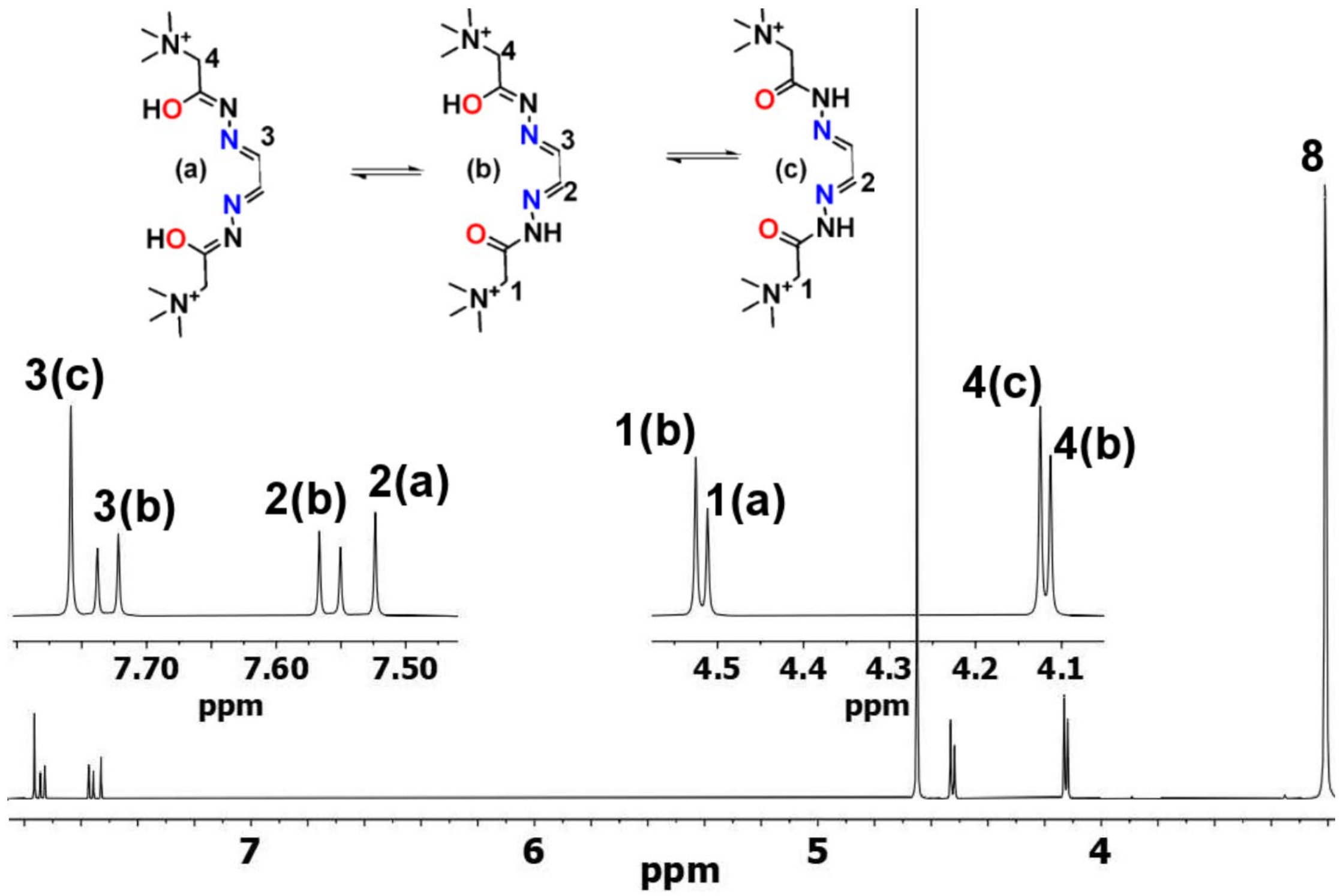


**Figure S4**. $^{1}$H NMR of the aqueous solution of $H_2glyxGT^{2+}$ pD = 7.0.

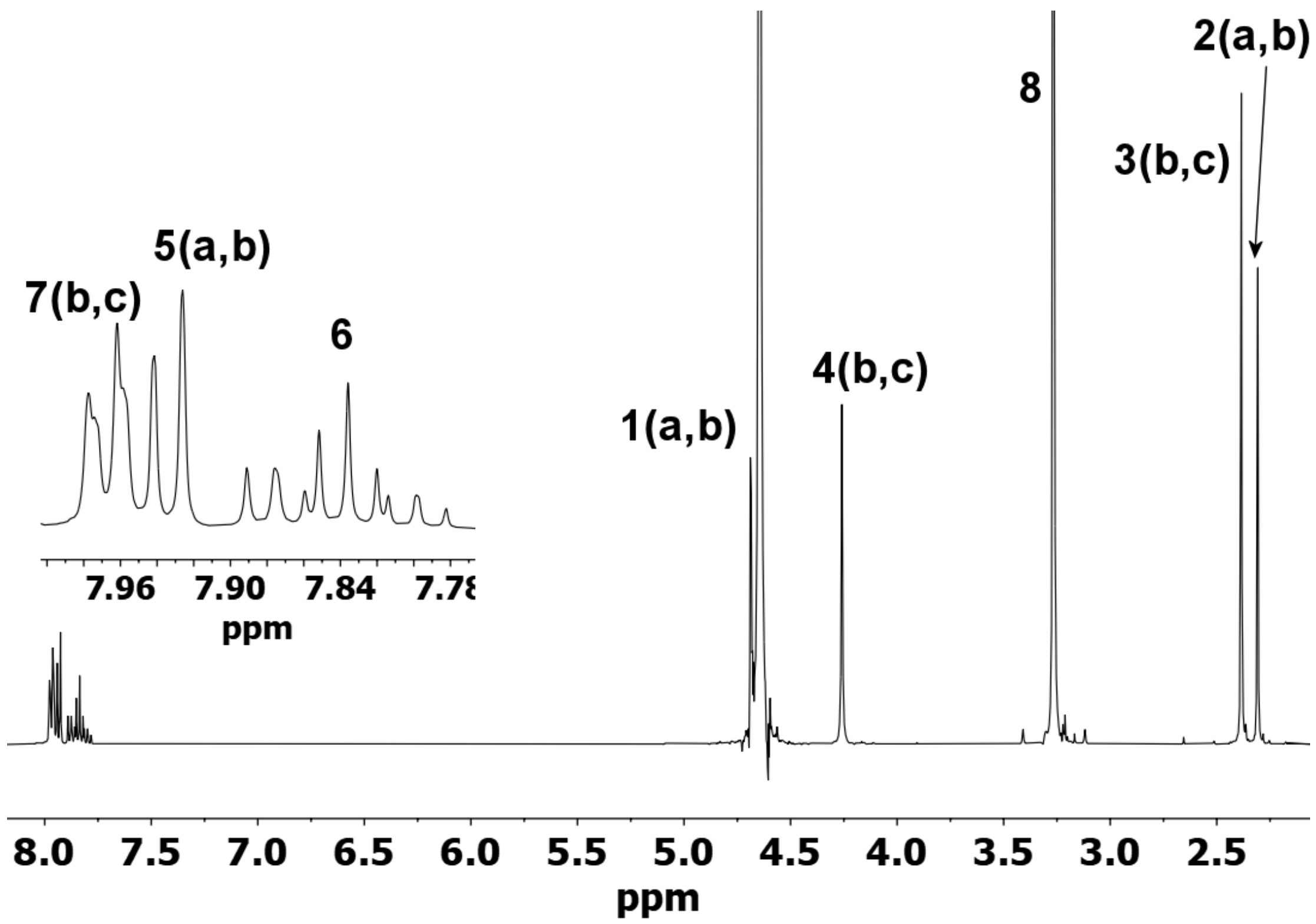


**Figure S5**. $^{1}$H NMR of the aqueous solution of $H_2dapGT^{2+}$ (4.00 mM) pD = 7.0.

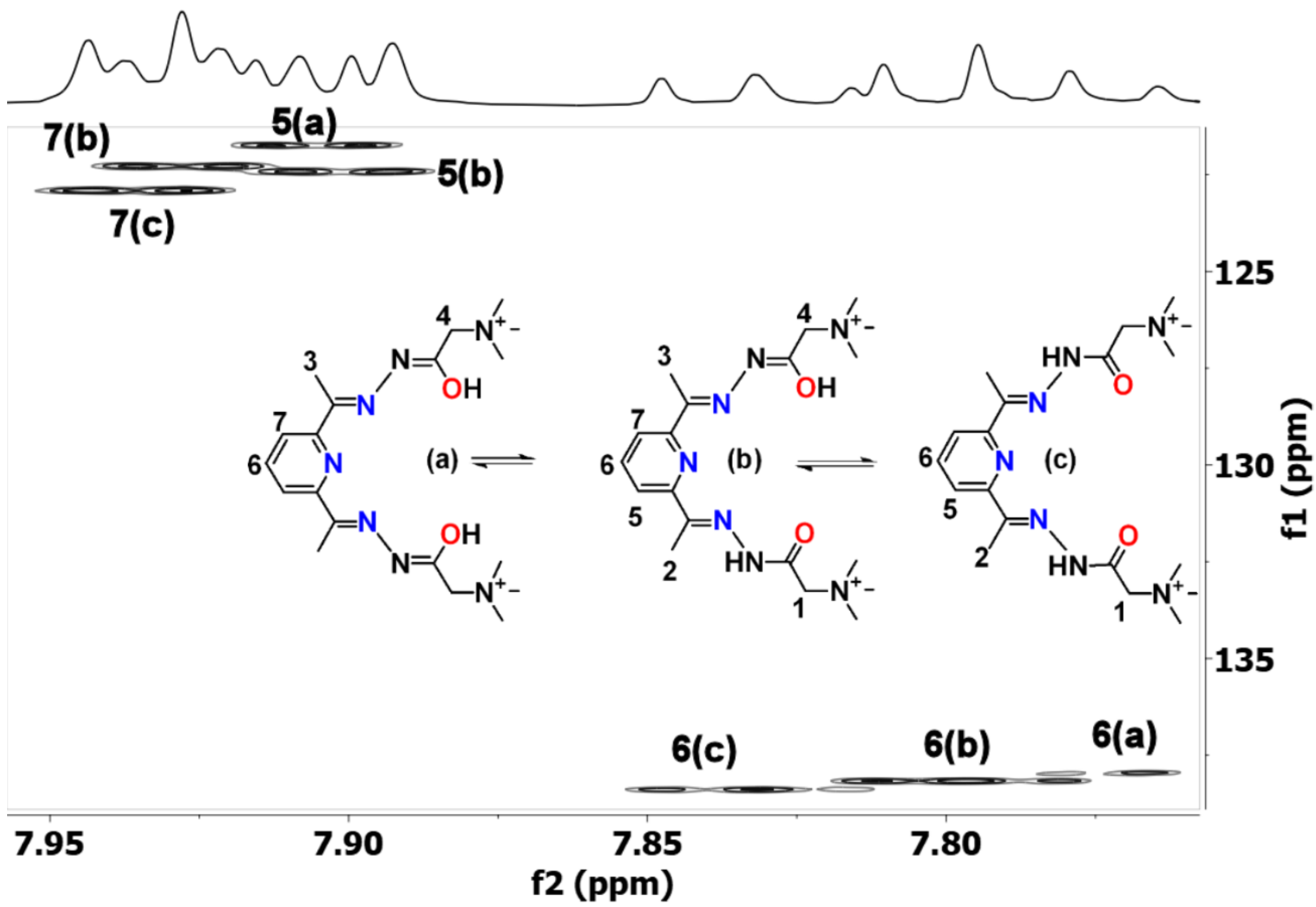


**Figure S6**. 2D {$^{1}H$,$^{13}C$} grHSQC NMR (aromatic region) of the aqueous solution of $H_2dapGT^{2+}$ at pD = 7.0.

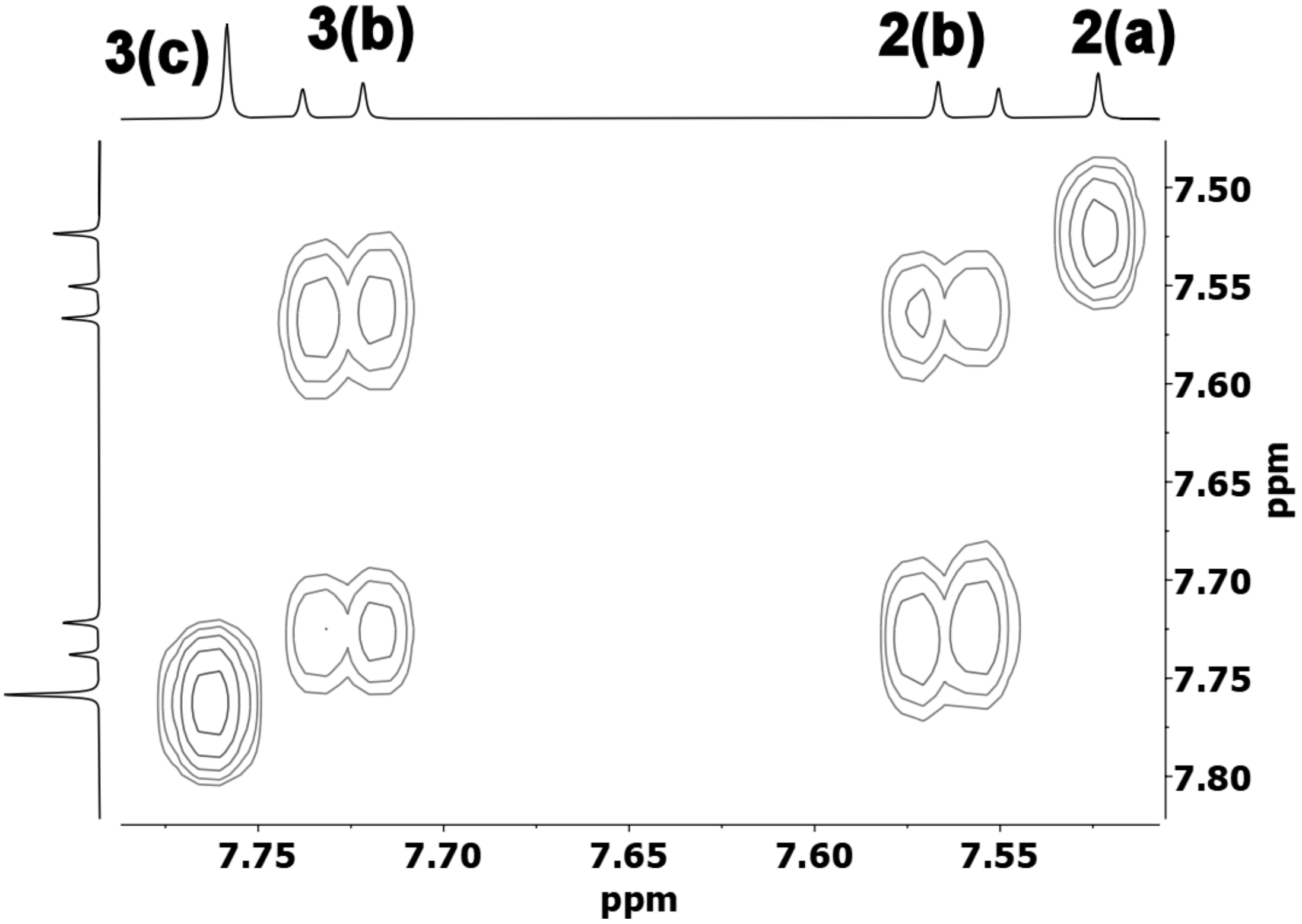


**Figure S7**. 2D {$^{1}$H} grCOSY NMR of the aqueous solution of $H_2glyxGT^{2+}$ (4.00 mM) pD = 5.3.

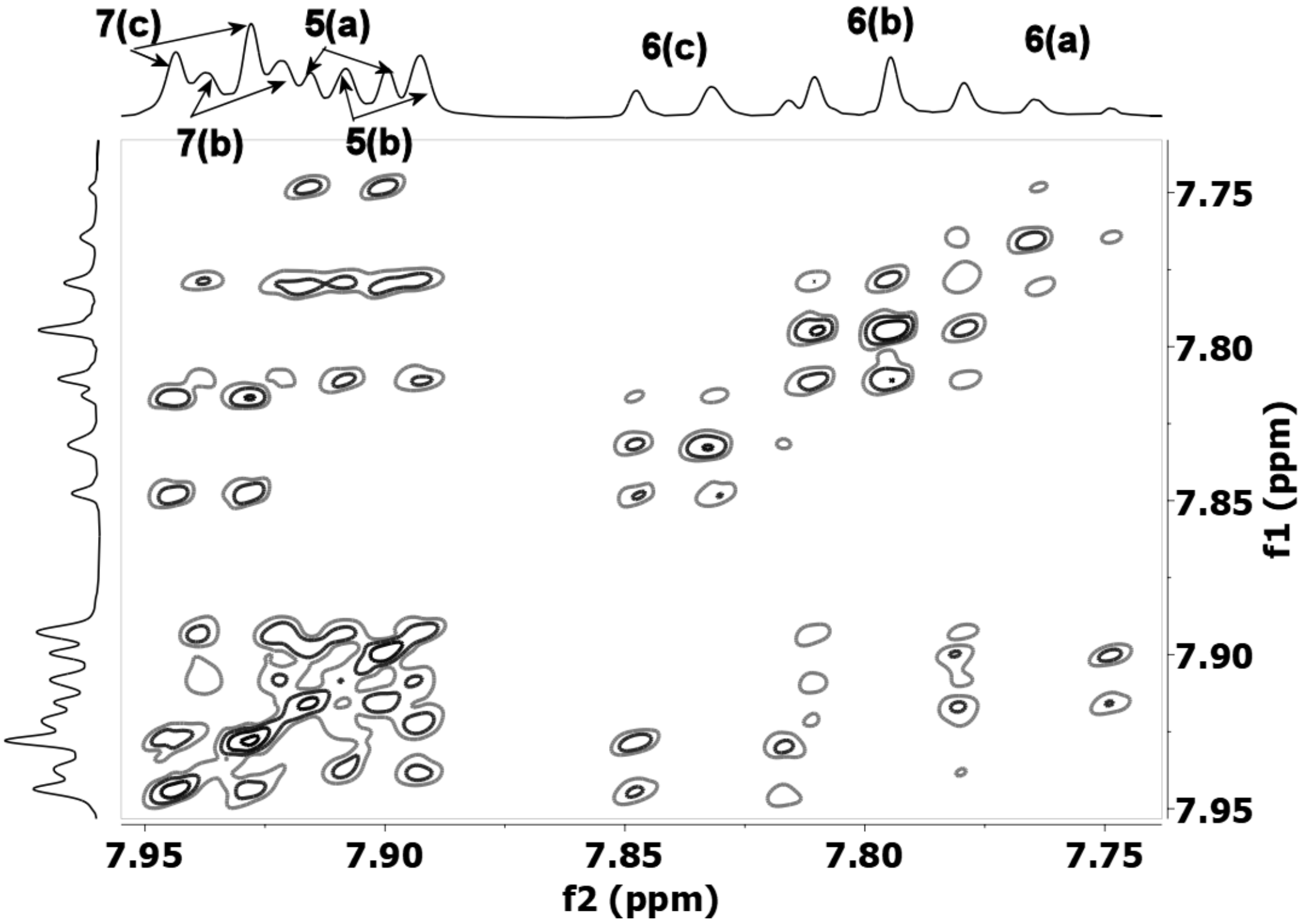


**Figure S8**. 2D {$^{1}$H} grCOSY NMR (aromatic region) of the aqueous solution of $H_2dapGT^{2+}$ (4.00 mM) at pD = 7.0.

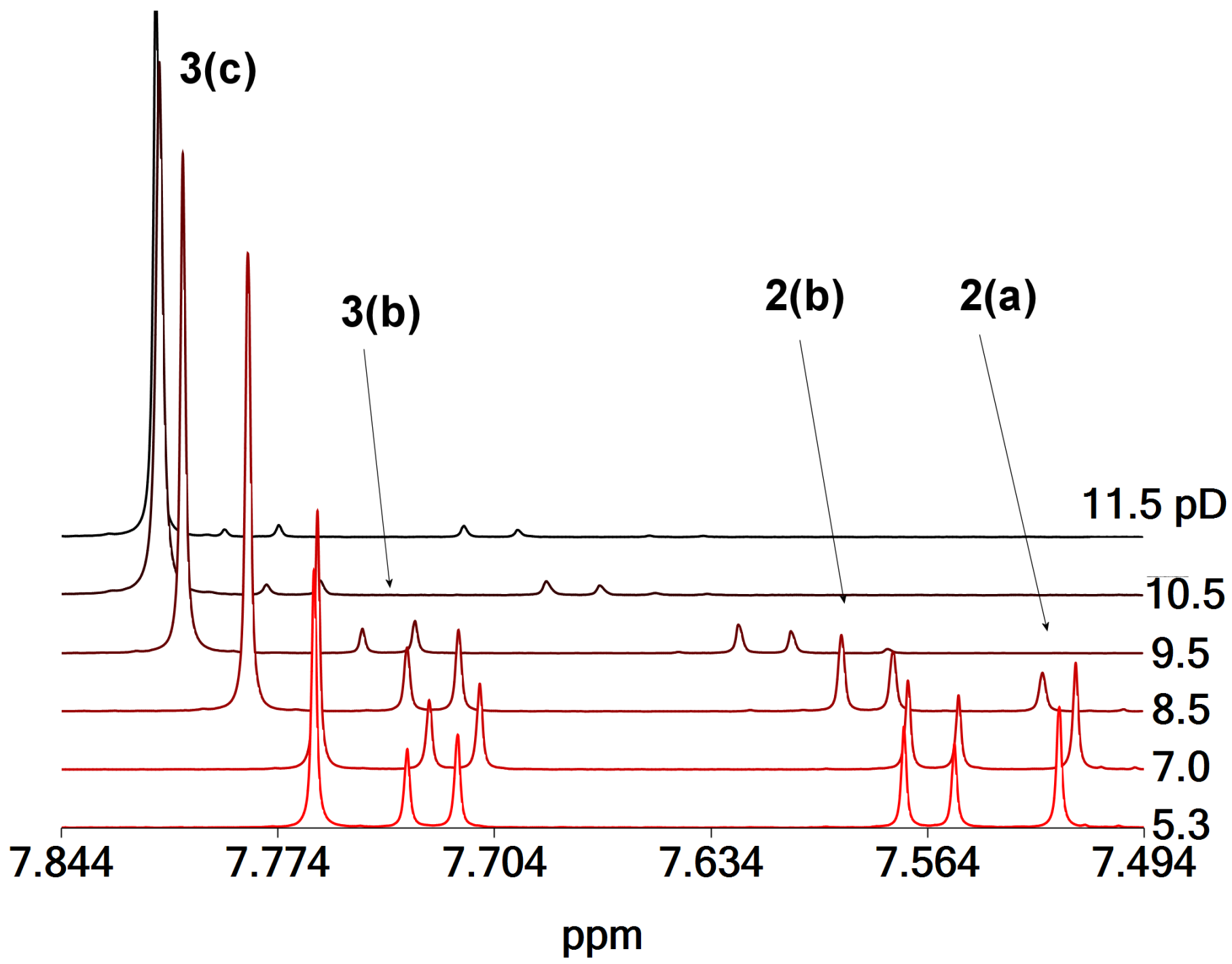


**Figure S9.** $^{1}$H NMR of the aqueous solution of $H_2glyxGT^{2+}$ (4.00 mM) *vs* pH.

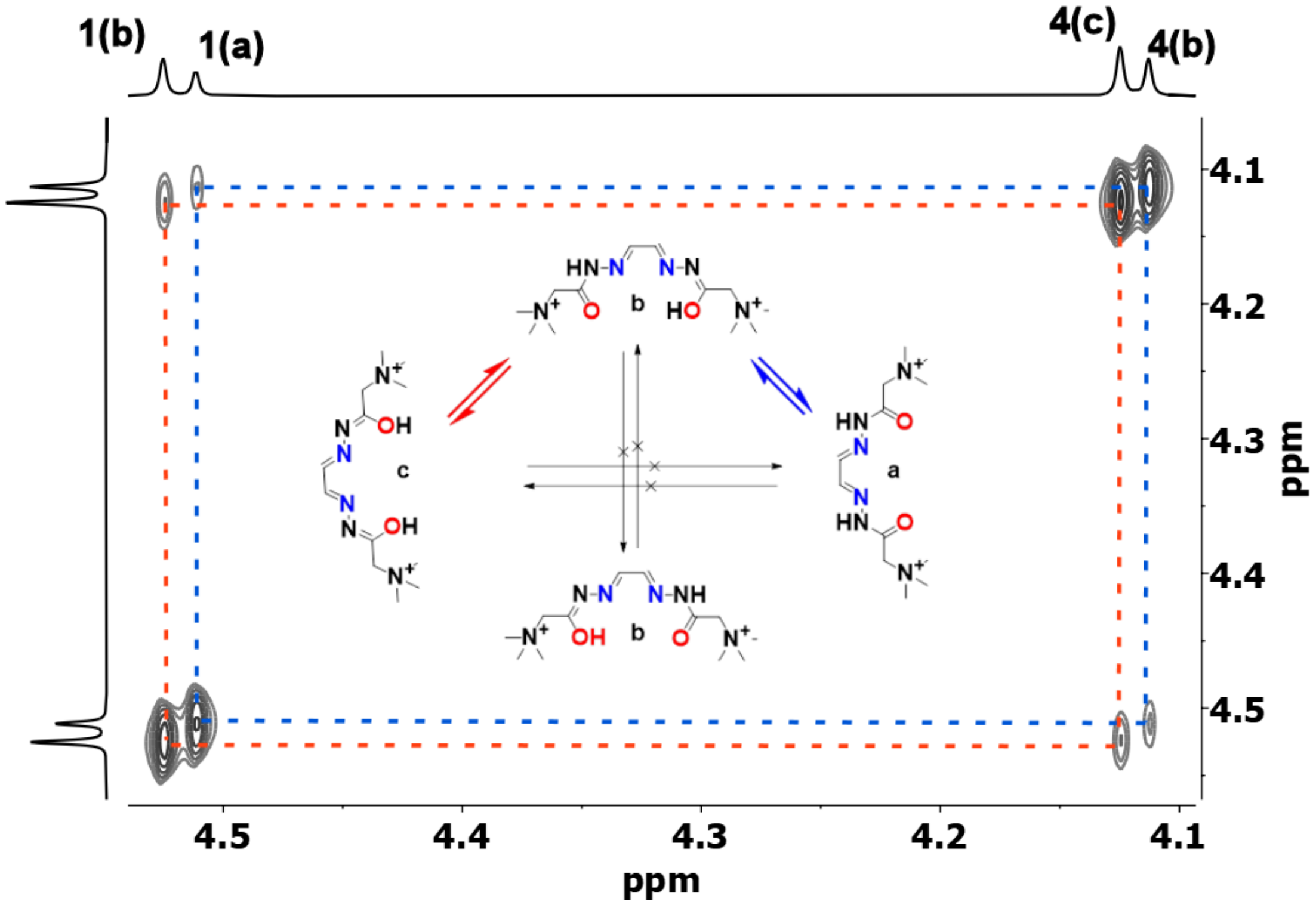


**Figure S10.** 2D {$^{1}$H} grEXSY NMR of the aqueous solution of $H_2glyxGT^{2+}$ (4.00 mM) at pD = 7.0, assignments and exchange mechanism.

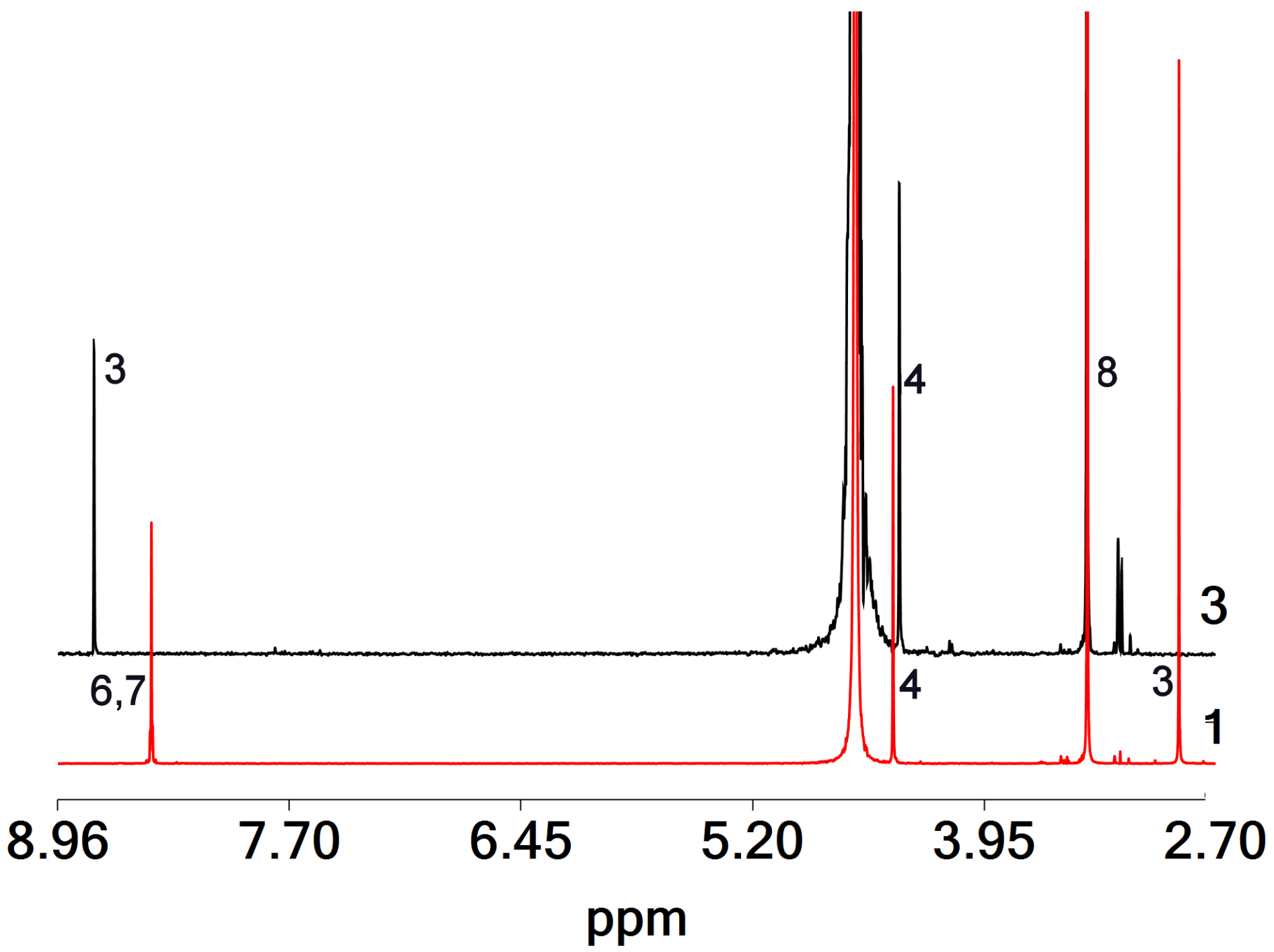


**Figure S11**. $^{1}H$ NMR of the aqueous solution of **1** (4.00 mM) and **3** (4.00 mM) pD = 7.0.

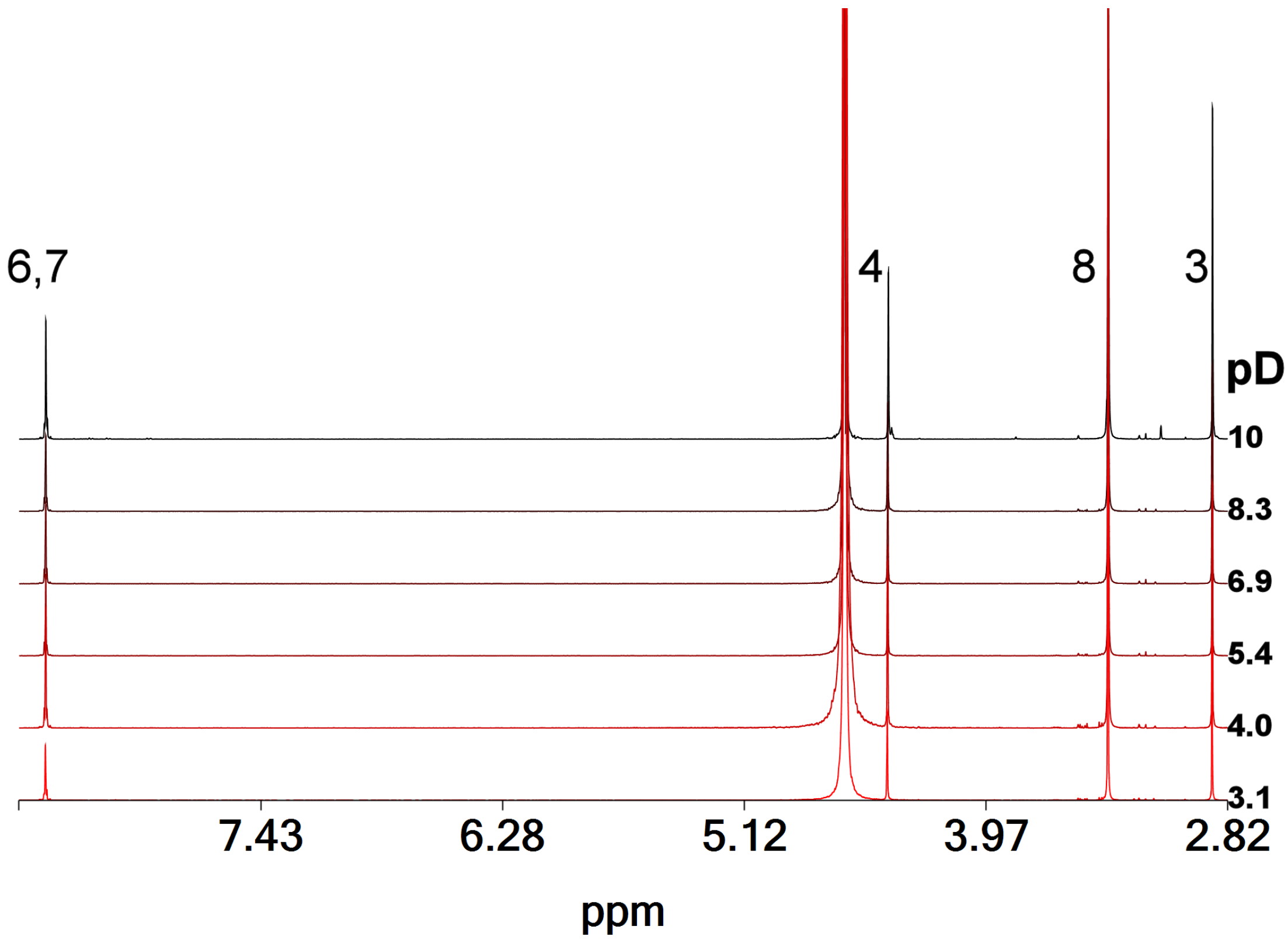


**Figure S12**. $^{1}$H NMR spectra of aqueous solution (4.00 mM) of complex **1** at different pDs.

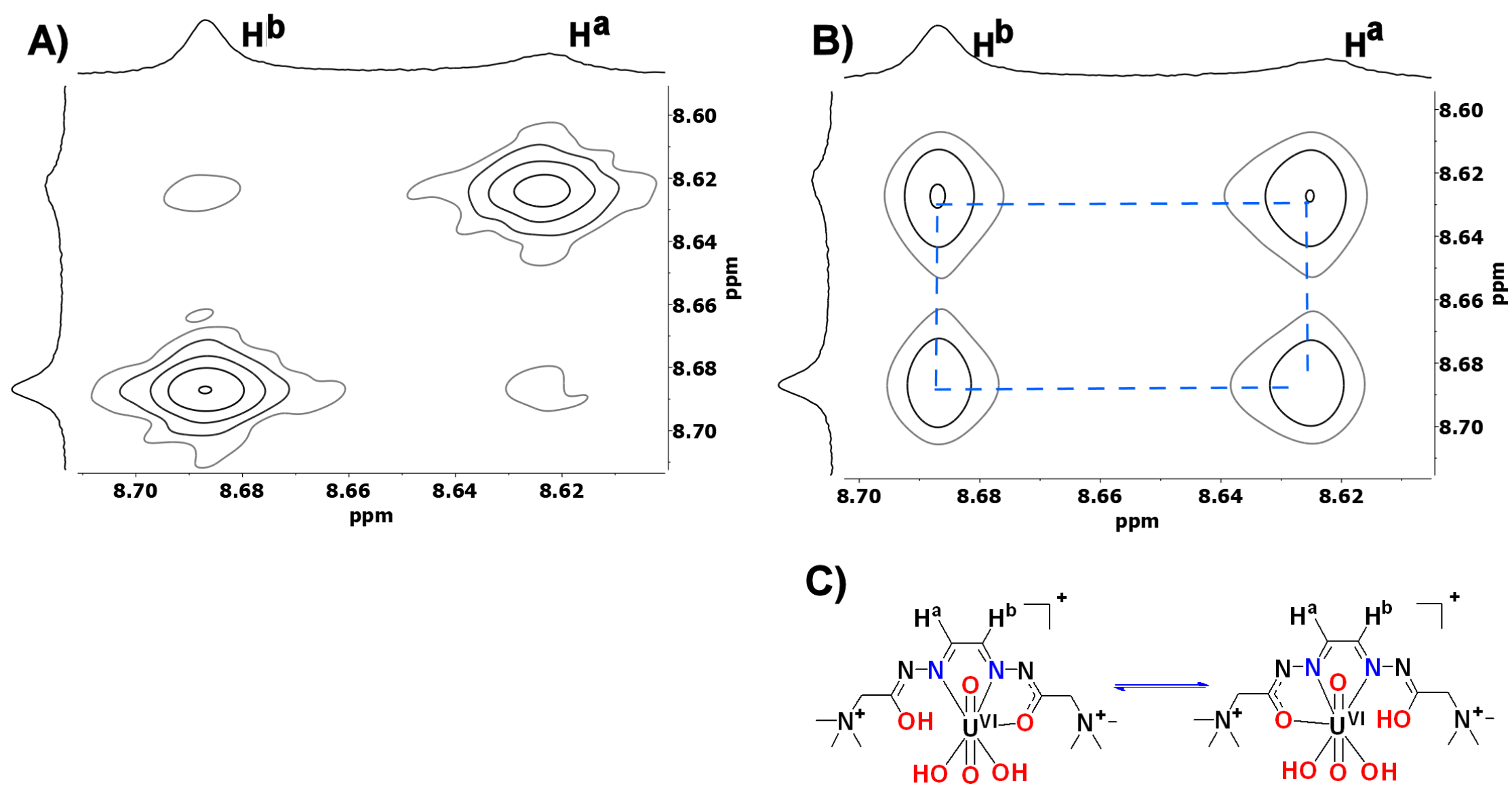


**Figure S13**. (A) 2D {$^{1}$H} grCOSY NMR spectra (B) 2D {$^{1}$H} grEXSY NMR spectra (C) exchange mechanism, of a $D_2O$ solution of complex **1** (4.00 mM) at pD=10.5.

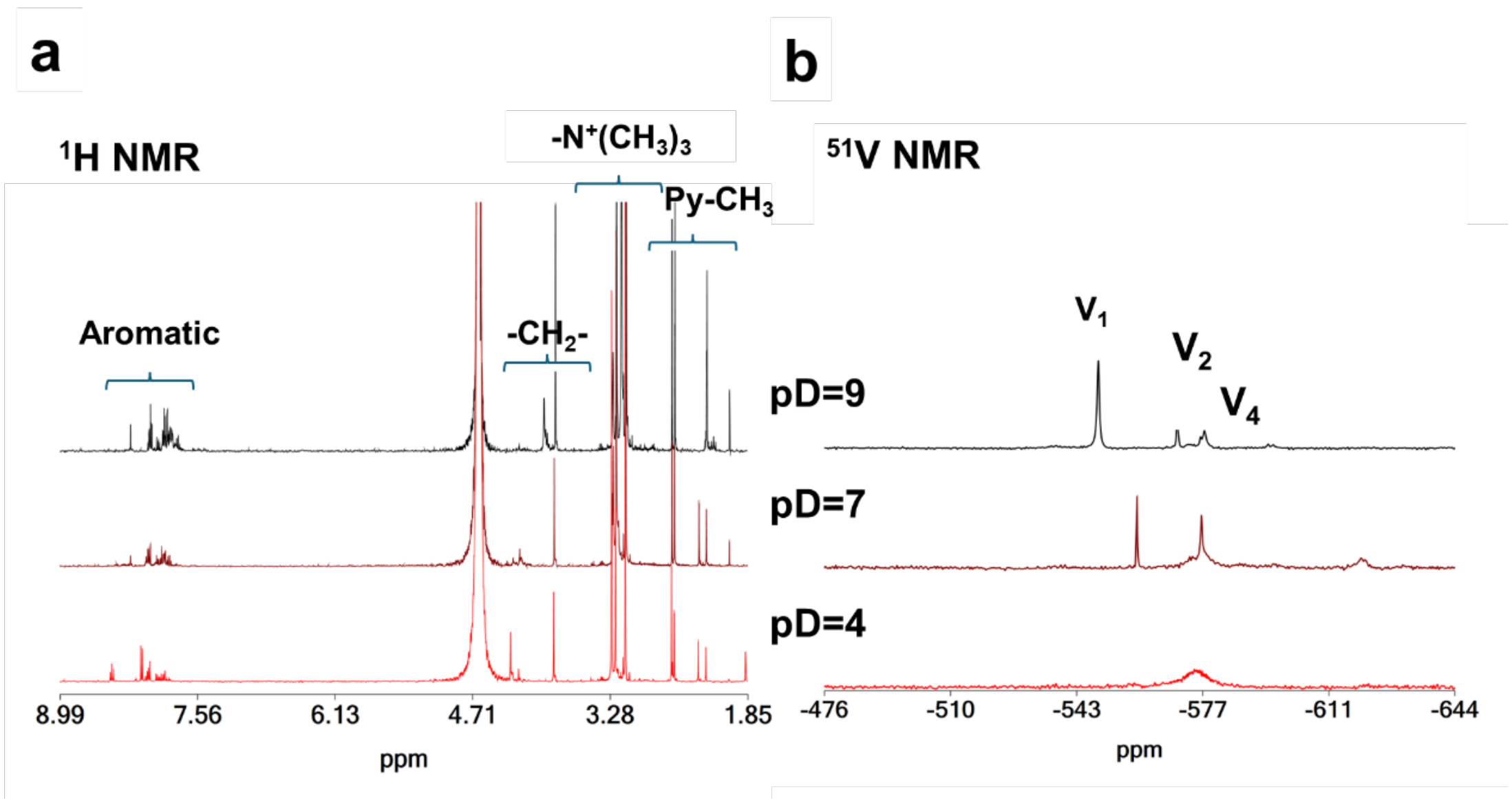


**Figure S14**. (A) $^{1}H$ NMR and (B) $^{51}V$ NMR for solutions of $H_2dapGT^{2+}$ (4.00 mM) and $NaVO_3$ (4.00 mM) at pD 4, 7 and 9.

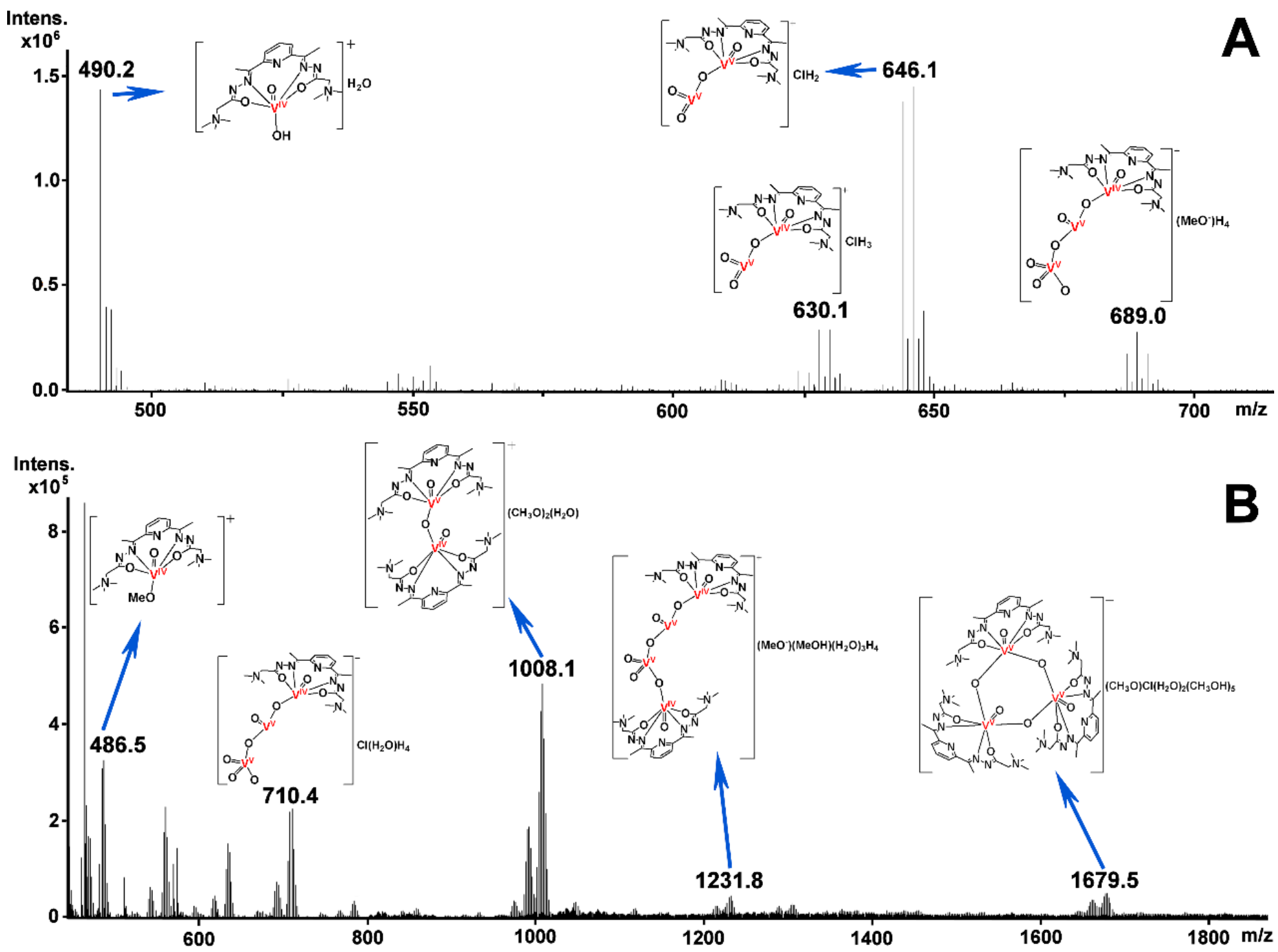


**Figure S15.** ESI-MS of the reaction mixture of $NaVO_3$ (4 mM) and the ligand $H_2dapGT^{2+}$ (2 mM) in $H_2O$:MeOH at (A) pH = 3 and (B) pH = 9.

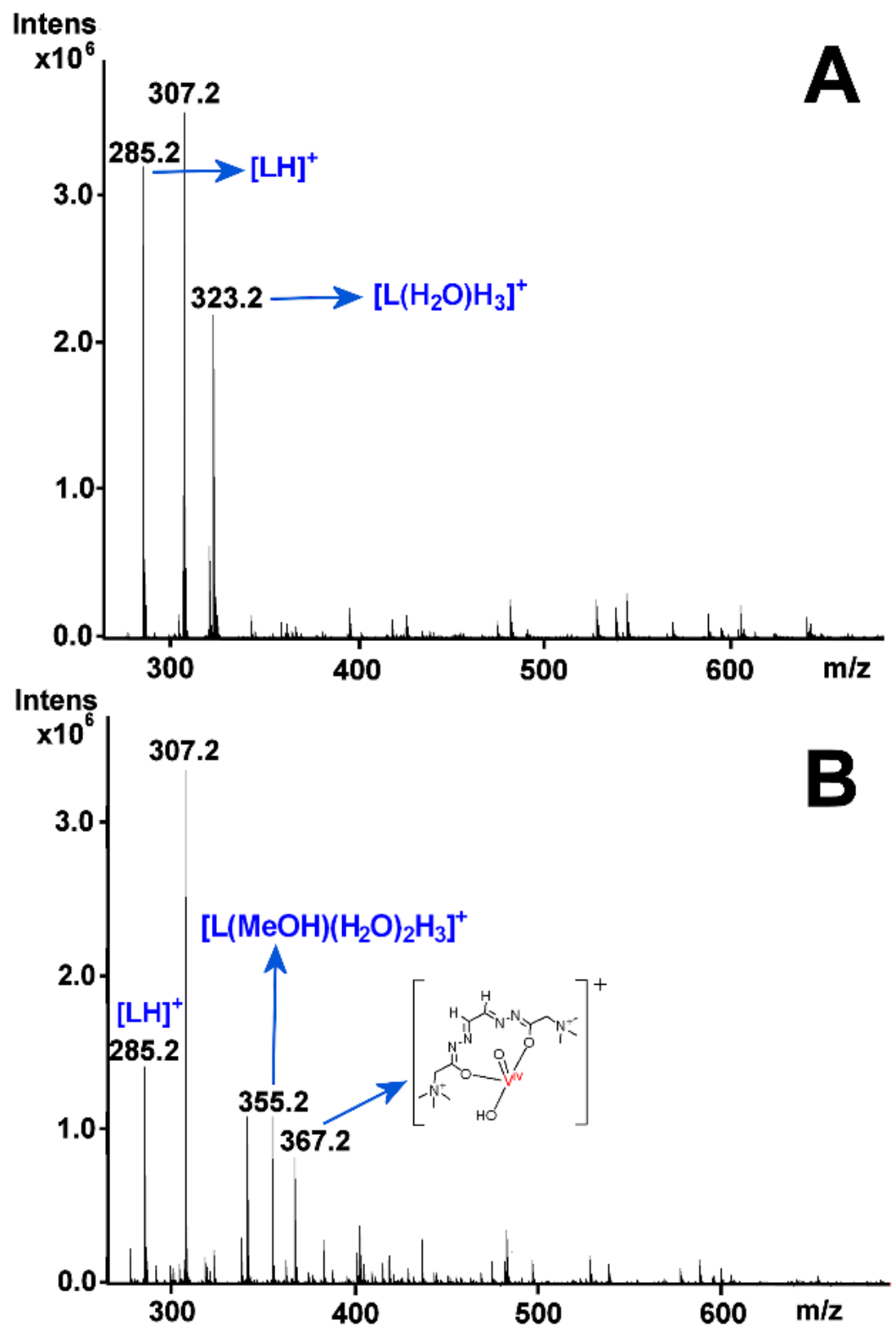


**Figure S16.** ESI-MS of the reaction mixture of $NaVO_3$ (4 mM) and the ligand $H_2glyxGT^{2+}$ (2 mM) in $H_2O$:MeOH at (A) pH = 3 and (B) pH = 9.

This approach aimed to identify any species that may be generated during the formation of vanadium-based complexes in solution. The potential identification of intermediate species could yield insights into the mechanistic details of $V^VO_2^+$ ions and ligands. For comparison, the spectra of the two ligands were recorded under the same experimental conditions (Figure S13). The isotopic envelopes can be attributed to the intact ligands with different counterions and protonation states. ESI-MS studies of the reaction mixtures were performed in positive ionization mode. The identified species formed instantly upon mixing $NaVO_3$ (4 mM) and $H_2dapGT^{2+}$ (2 mM) in aqueous medium. Figure S25 shows the mass spectra of species formed in the reaction mixture. Singly charged distribution envelopes at m/z 486.5 and 490.2 correspond to $[V^{IV}O(dapGT)(OH)(H_2O)]$+ and $[V^{IV}O(dapGT)(MeOH)]^+$. At elevated m/z values,

higher nuclearity species were observed due to condensation processes at elevated pH. Higher nuclearity species with isotopic envelopes at m/z 630.1, 646.1, 689.0, 710.4, 1008.1, 1231.8 and 1679.5, had formulae $[V^{IV}OV^{V}O_3(dapGT)(Cl)(H_3)]^+$, $[V^{V}OV^{V}O_2(dapGT)(Cl)(H_2)]^+$, $[V^{IV}O(V^{V}O_3)_2(dapGT)$ $(MeO^-)(H_4)]^+$, $[V^{IV}O(V^{V}O_3)_2(dapGT)(Cl)(H_2O)(H_4)]^+$, $[V^{V}O(dapGT)OV^{IV}O(dapGT)(MeO^-)_2(H_2O)]^+$, $[V^{IV}O(dapGT)(V^{V}O_3)V^{IV}O(dapGT)$ $(MeO^-)(MeOH)(H_2O)_3H_4]^+$ and $\{[V^{IV}O(dapGT)(O)]_3Cl(H_2O)_2(MeO^-)(MeOH)_5]^+$. The interaction between $NaVO_3$ and $H_2glyxGT^{2+}$ ligand showed species forming immediately upon combining $NaVO_3$ (4 mM) and $H_2glyxGT^{2+}$ (2 mM) at pH 3-9, though speciation was more restricted compared to the $V^{V}O_2^+/H_2dapGT^{2+}$ interaction and pH-dependent. Figure S26 shows singly charged envelopes at m/z 285–323 for the free ligand, while the complex formed at pH 7–9 with an envelope at m/z 367.1 ($[V^{IV}O(glyxGT)(OH)]^+$).

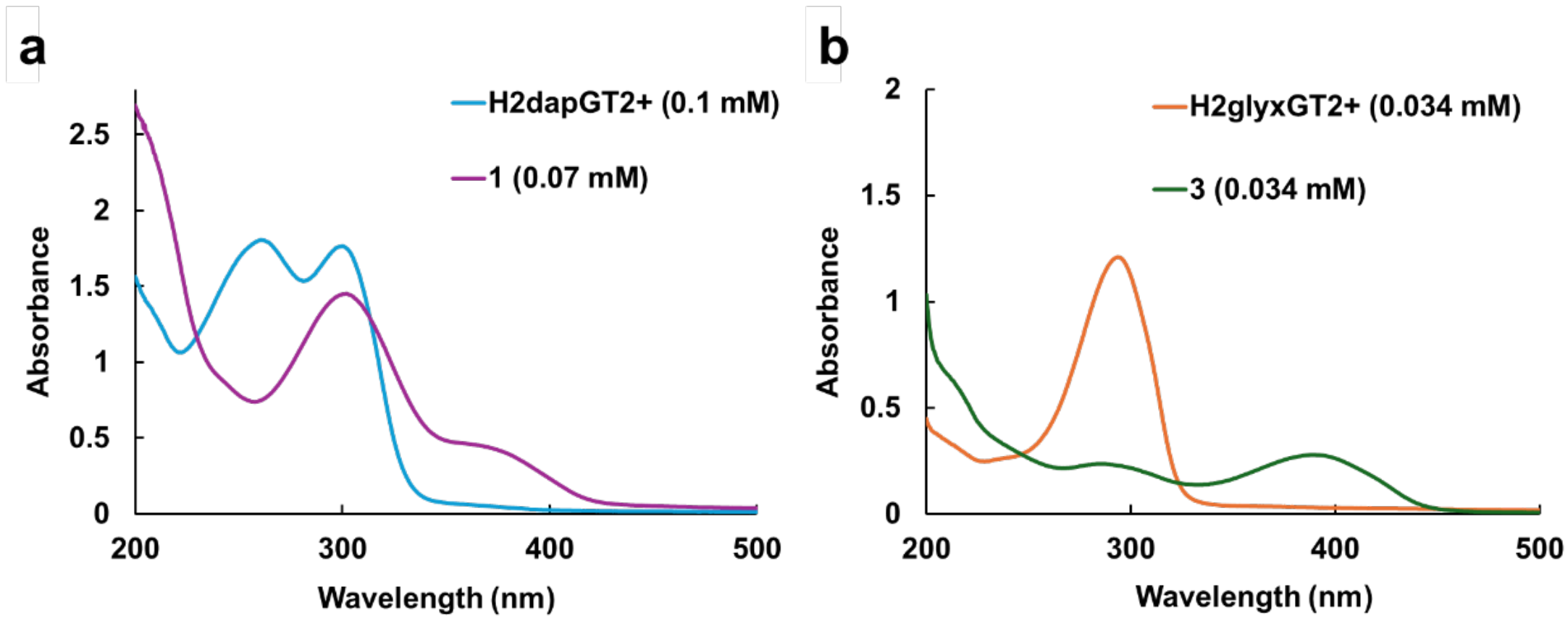


**Figure S17**. UV-Vis spectra (aqueous solution, pH=7) of (A) $H_2dapGT^{2+}$ and **1** and (B) $H_2glyxGT^{2+}$ and **3**.

*UV-vis Spectroscopy*

The spectrum of $H_2dapGT^{2+}$ displays two peaks at $\lambda_{max}$ = 263 and 301 nm. In the corresponding complex **1**, the peak at $\lambda_{max}$ = 301 nm is retained, while a shoulder emerges between 346 nm and 430 nm ($\varepsilon = 0.55 \times 10^4\ M^{-1}cm^{-1}$), attributed to LMCT. $H_2glyxGT^{2+}$ exhibits a strong absorption peak at $\lambda_{max}$ = 295 nm. In addition to the peak at 295 nm, complex **3** exhibited an LMCT peak at $\lambda_{max}$ = 394 nm ($\varepsilon = 0.81 \times 10^4\ M^{-1}cm^{-1}$). A solution of complex **4** at pH 3.0 gave intraligand peaks at $\lambda_{max}$ = 238 ($\varepsilon = 1.82 \times 10^4\ M^{-1}cm^{-1}$), $\lambda_{max}$ = 282 ($\varepsilon = 1.65 \times 10^4\ M^{-1}cm^{-1}$), and 314(sh) ($\varepsilon = 1.26 \times 10^4\ M^{-1}cm^{-1}$), and an LMCT peak at $\lambda_{max}$ = 376 ($\varepsilon = 0.27 \times 10^4\ M^{-1}cm^{-1}$).

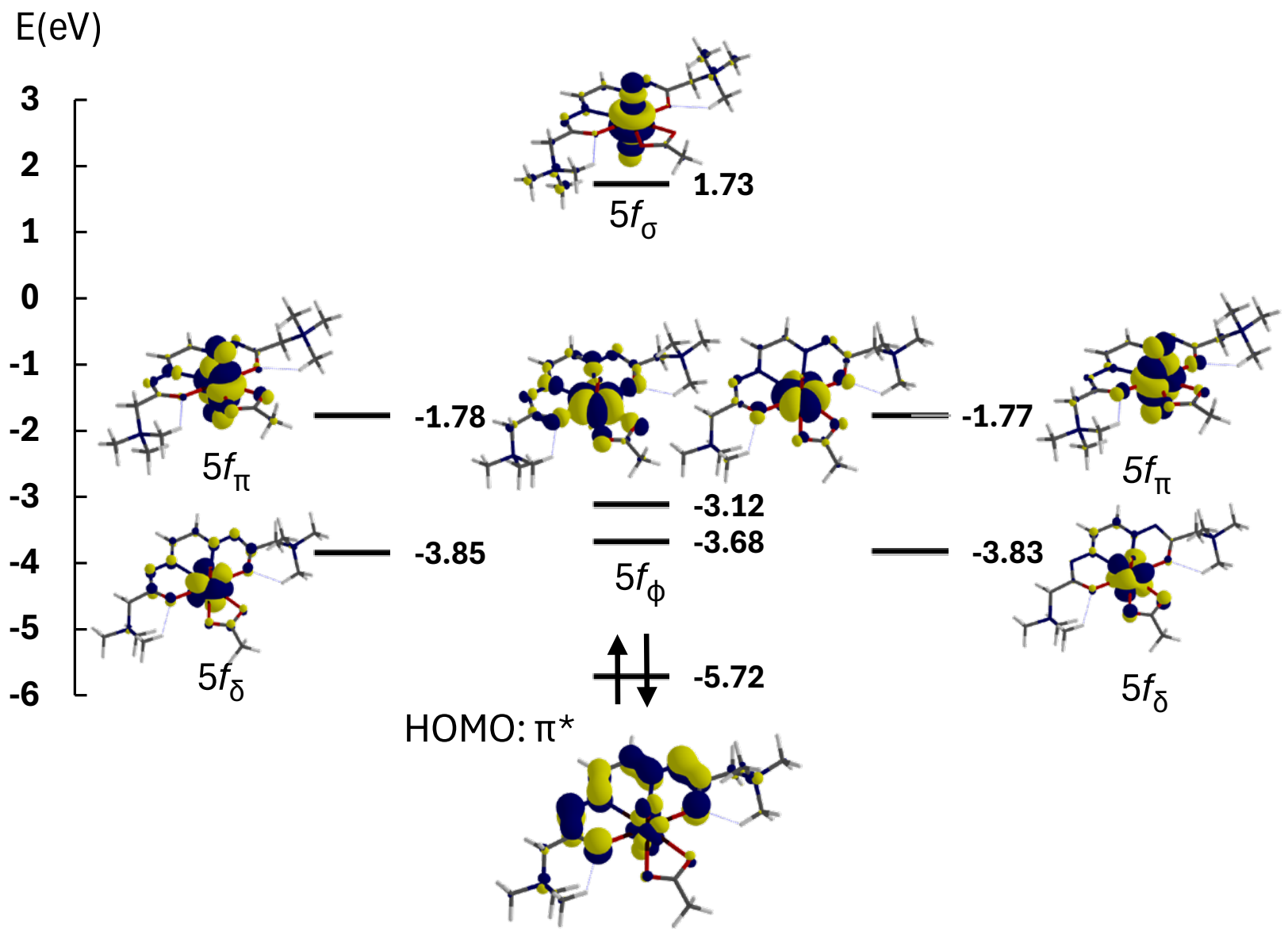


**Figure S18.** Kohn-Sham orbitals and ligand field splitting of the 5f orbitals in a hexagonal bipyramid environment.

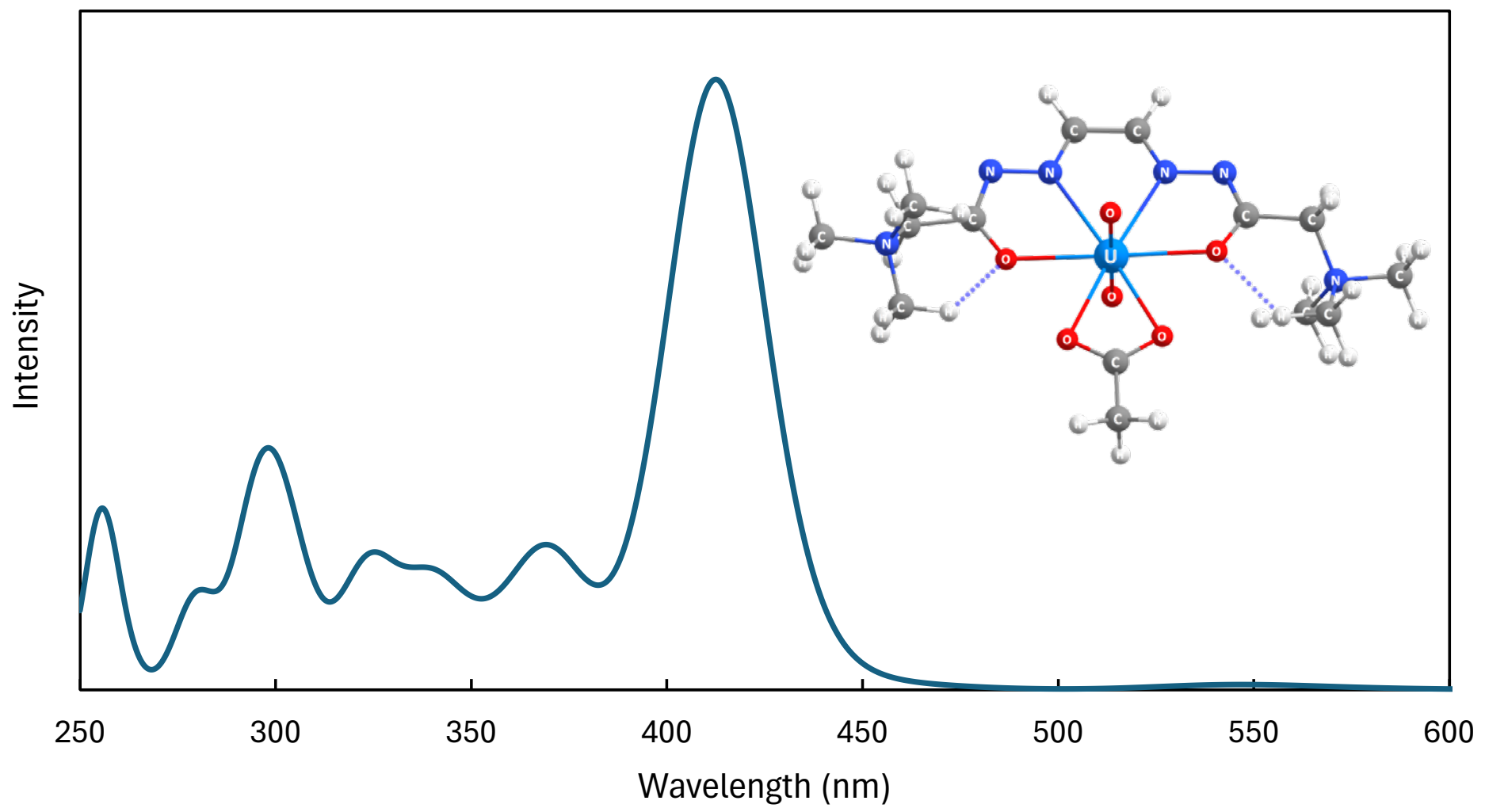


**Figure S19.** Calculated UV-Vis spectrum of complex **3**.

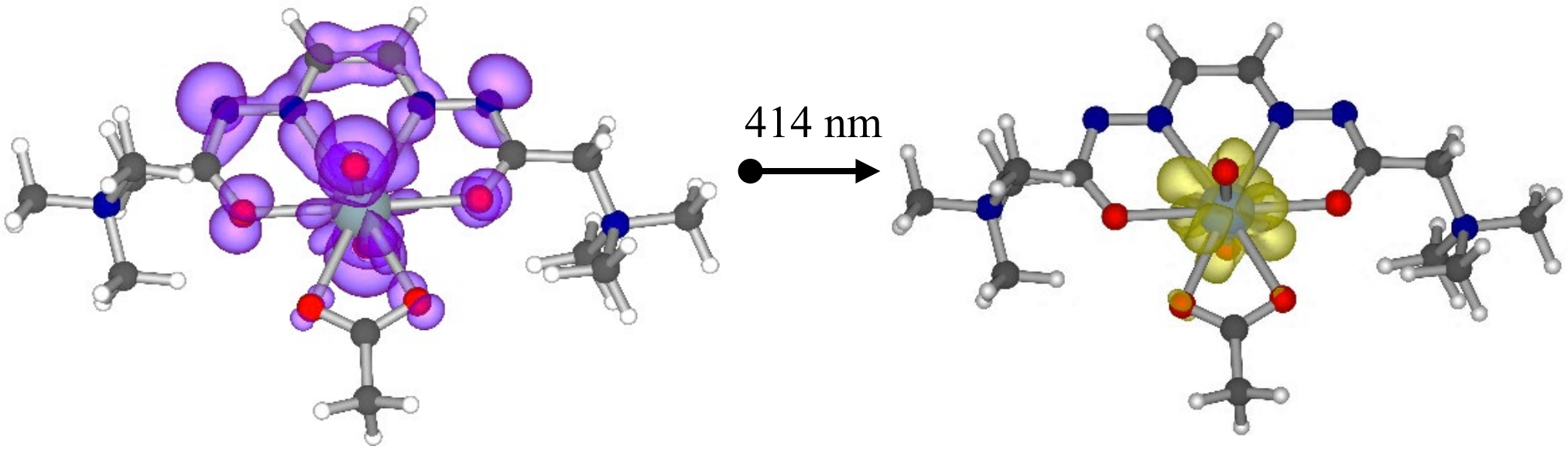


**Figure S20.** Natural difference density of SOC state 65 in complex 3 at 0.0005 a.u. isocontour. Purple represents electron depletion, yellow electron gain.

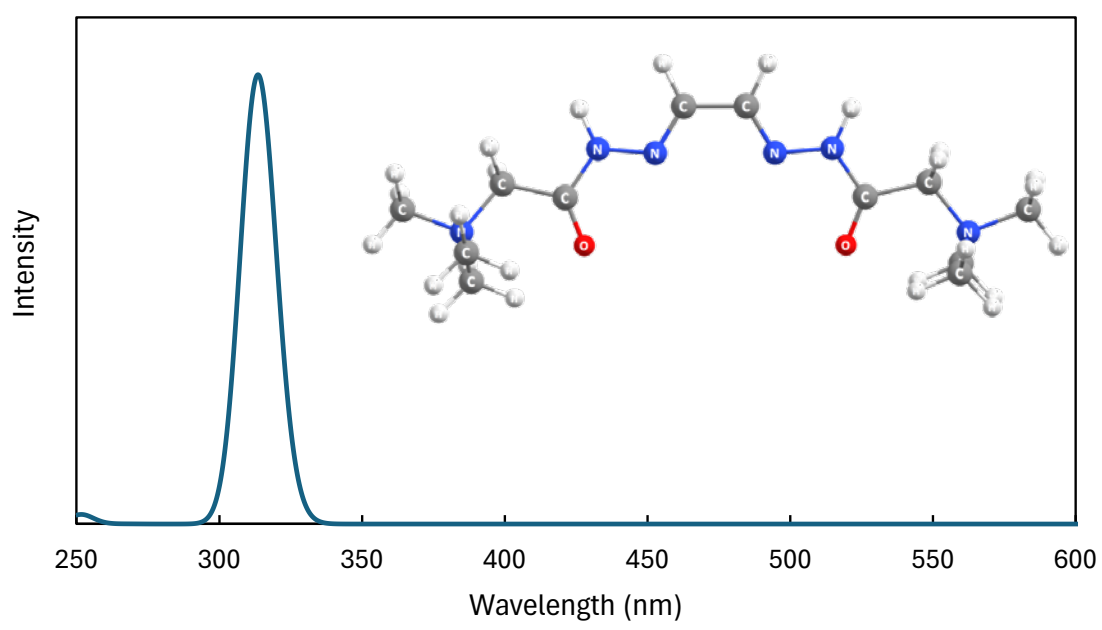


**Figure S21.** UV-Vis spectrum of glyxGT$^{2+}$.

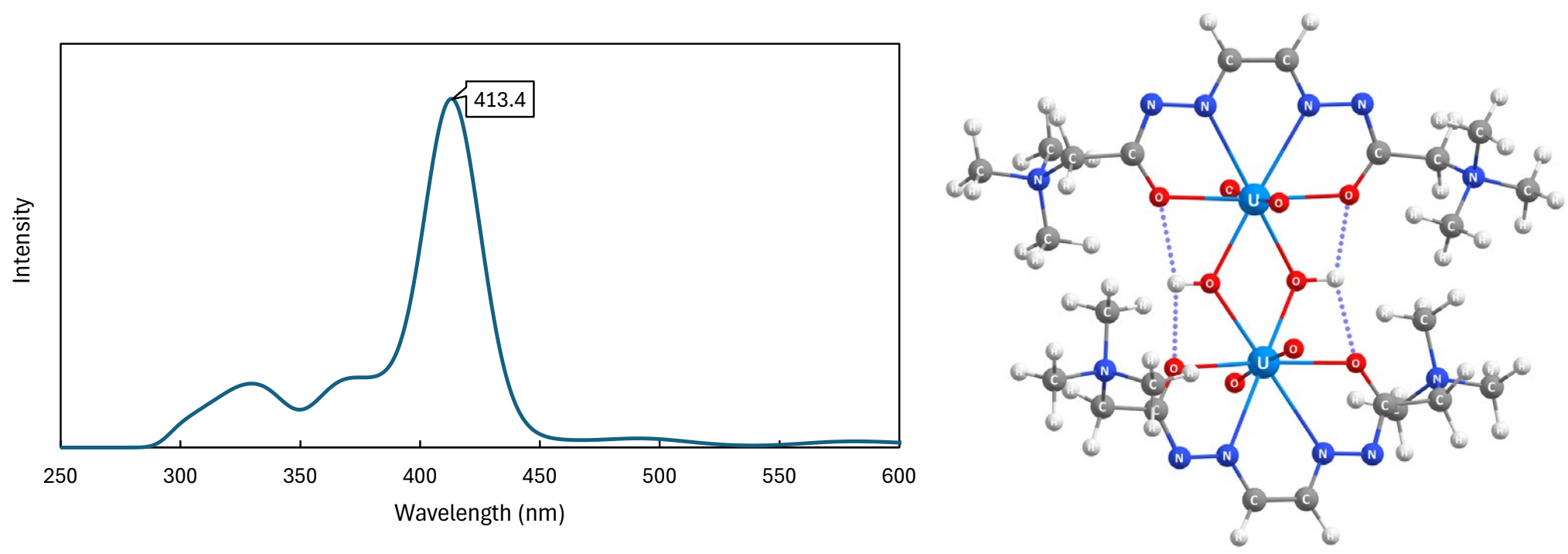


**Figure S22**. Calculated UV-Vis spectrum of the $[(U^{VI}O_2)glyxGT(\mu\text{-}OH)]_2^{2+}$ complex.

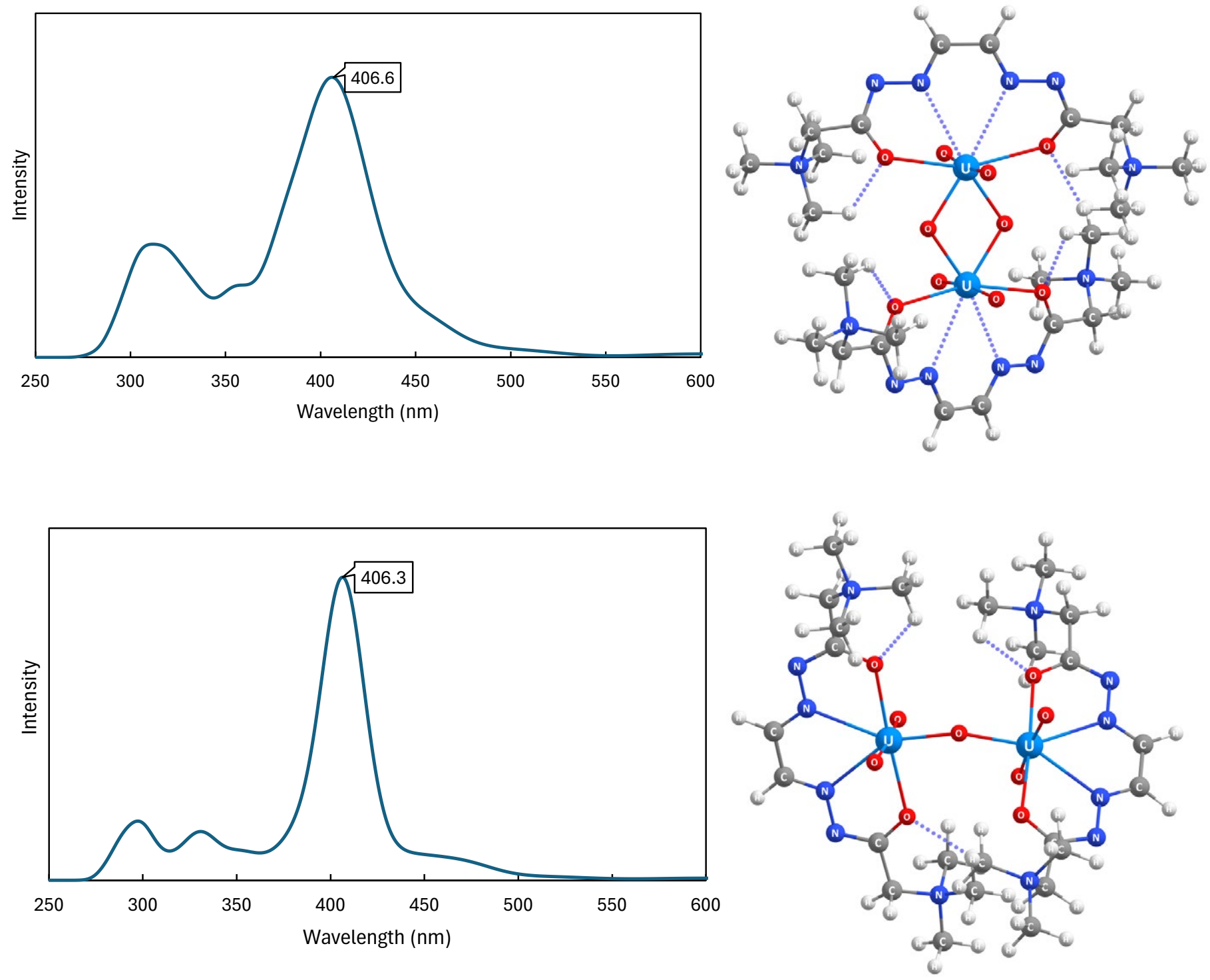


**Figure S23**. Calculated UV-Vis spectrum of the optimised dioxo (top) and monoxo (bottom) bridged dimers.

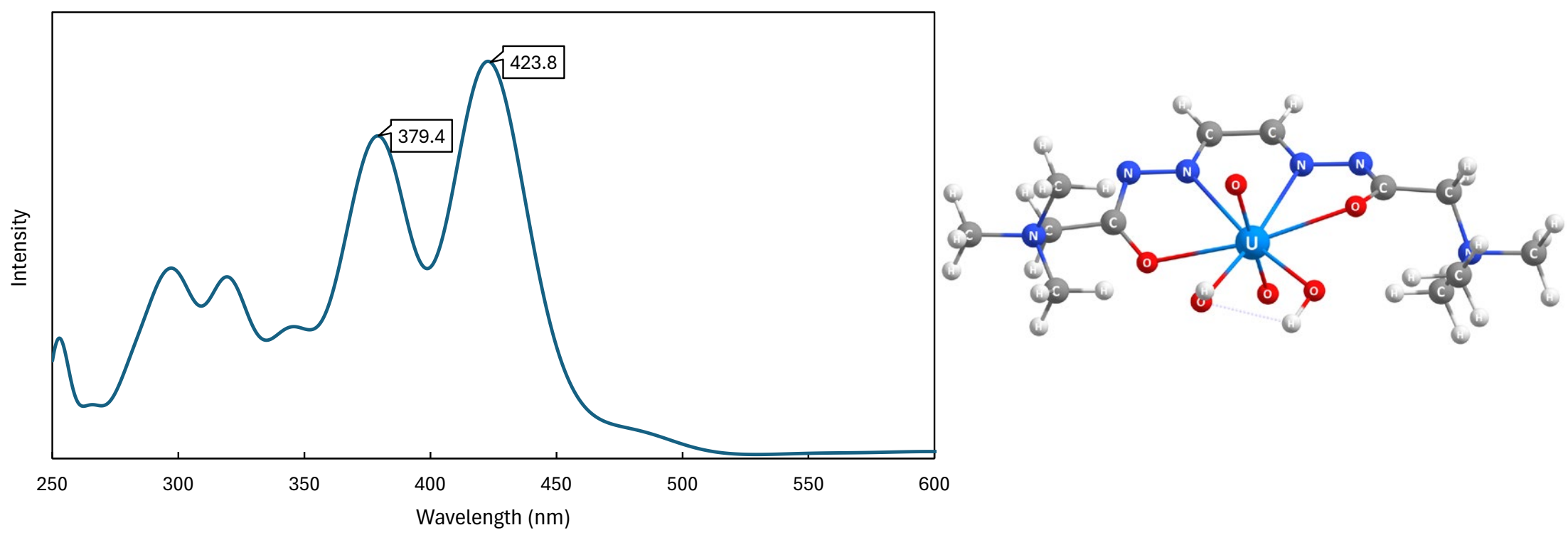


**Figure S24.** UV-Vis spectrum of [($U^{VI}O_2$)$(OH)_2$($\kappa^4$-glyxGT)].

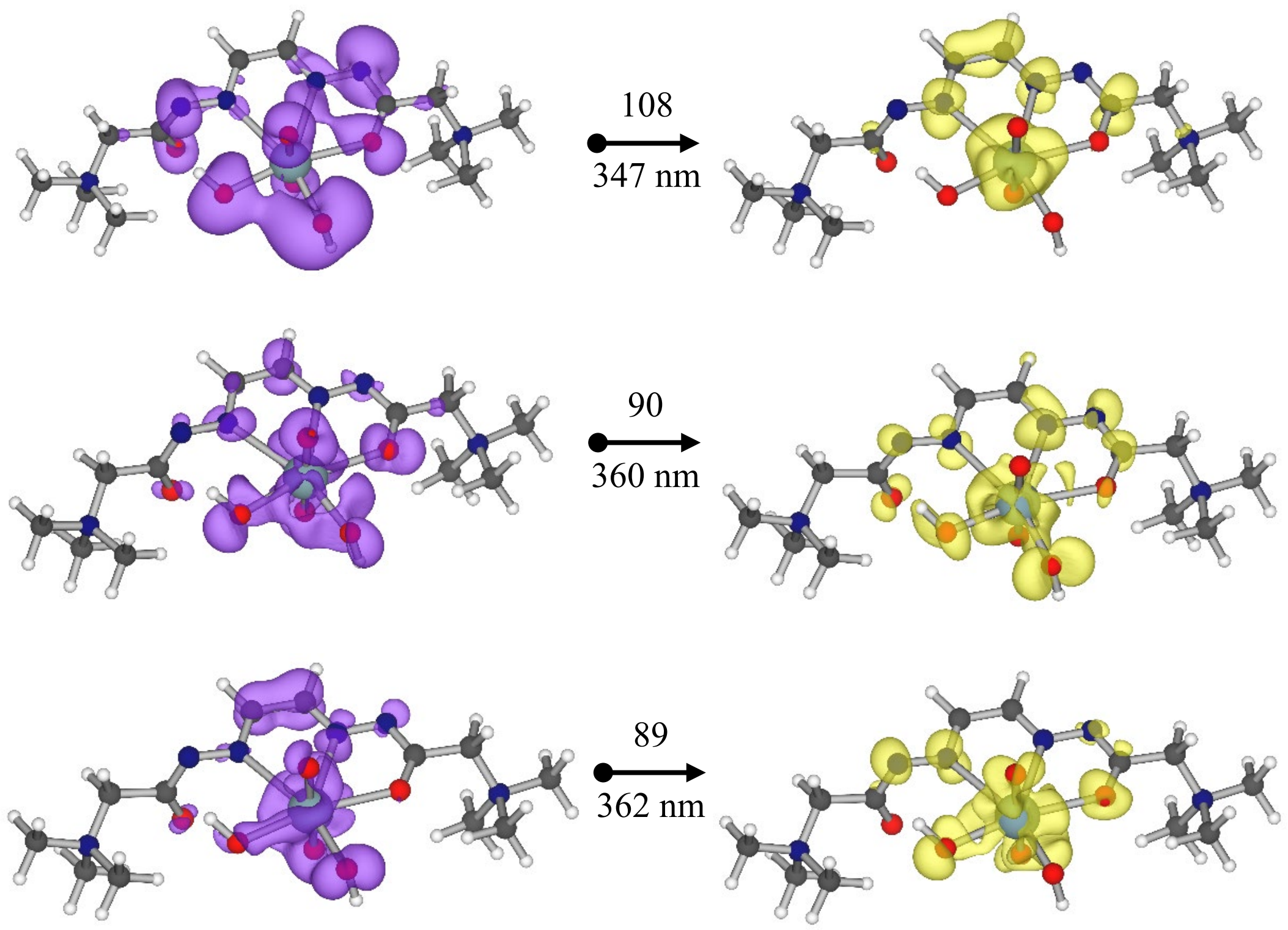


**Figure S25.** Transition difference densities of the most intense spinor states (108, 90 and 89 see Table 1) at 0.0005 a.u. isocontour. Purple represents electron depletion, yellow electron gain.

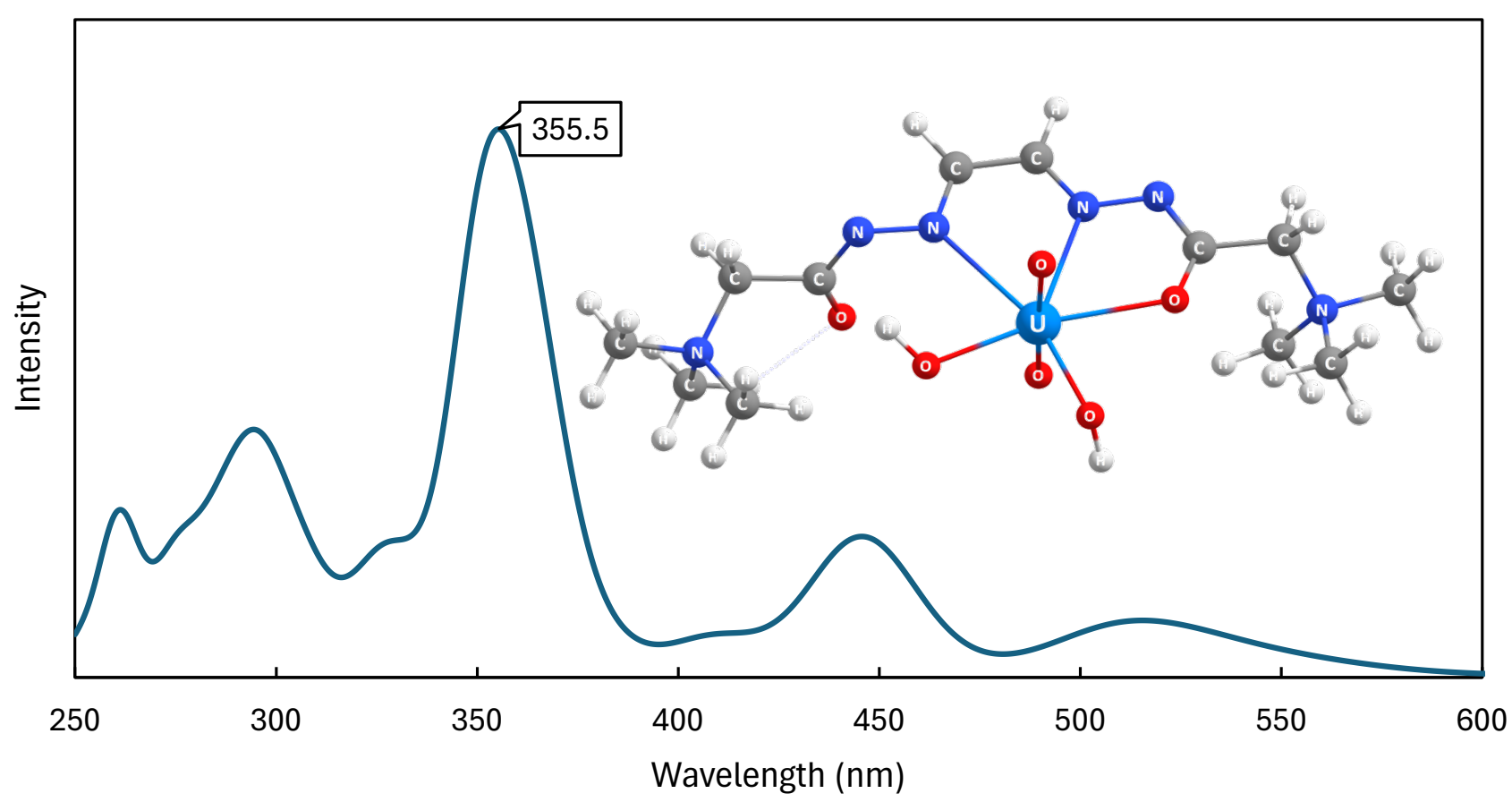


**Figure S26**. Calculated UV-vis spectrum of [($U^{VI}O_2$)$(OH)_2$($\kappa^3$-glyxGT)].

The electronic structure of **3** is that of a typical $U^{VI}O_2^{2+}$ complex with tightly packed 5*f*$_\phi$ and 5*f*$_\delta$ pairs in the region −3.8 −3.1 eV (Figure S14). The HOMO is mostly ligand-based, and although the LUMO+3 (−3.31 eV) is also ligand-based, most of the near valence region excited states will have ligand-to-metal charge transfer (LMCT) character. Both complex **3** and the free ligand are fairly well characterized in solution and will serve as a benchmark for the adopted methodology (TD-r2SCAN). All the structures were geometrically optimized, as specified in the section with computational details. The calculations were conducted with spin-orbit coupling (SOC), and the peaks were widened with a Gaussian function to compare them with the experimental spectrum. With respect to the electronic transitions, describing them in terms of individual occupied and virtual molecular orbitals is impractical, as the relevant excited states are highly delocalized in their composition, obscuring a clear interpretation of the transitions. Moreover, spin-orbit coupling, which results in a complex-valued linear combination of singlet and triplet transitions, adds another layer of complexity to the makeup of the relevant excited states. Therefore, it is simpler to analyze the excited state using natural difference densities that condense all the required information as real-valued isosurfaces. Based on the analysis of **3**, the simulated band in the visible region is situated at 414 nm (Figure S15), corresponding to the spin-orbit coupled state 65. Its nature is mostly due to ligand-to-metal charge transfer (LMCT) stemming from singlet 11 combined with singlet 18, triplet 22, and a collection of other states contributing under 4% (Table S5). The two signed difference densities for SOC state 65 are shown in Figure S16. The experimental spectrum records a band at 394 nm, which shows excellent agreement with the TDDFT-simulated spectrum. The UV-vis spectrum of the free $glyxGT^{2+}$ ligand was also calculated, and its absorption peak at 314 nm (Figure S17) agreed well with the experiment ($\lambda_{exp}$=295 nm). Both calculated

spectra appeared slightly redshifted compared to the experiment. To identify the species giving rise to the absorption band around 326 nm, a number of possible candidates were evaluated (Figures S18 and S19). It was then hypothesized based on ESI-MS evidence that a single uranyl unit $[(U^{VI}O_2)(OH)_2(\kappa^4\text{-glyxGT})]$ might be present at high pH values, *vide infra*. This species exhibited a dense spectrum, but its absorption wavelengths were still too far removed to be considered the missing species (9). However, NMR data indicate that the ligand is under-coordinated in solution, adopting $\kappa^3$ rather than $\kappa^4$ denticity.

This does have an acceptable deviation from the experimental value (356 nm vs. 326 nm), as shown in Figure 7. While the calculated species is neutral, the ESI/MS detects a cation with an identical composition to that of protonated nitrogen. There is a small energy difference of −24.7 kJ $mol^{-1}$ in favour of the undercoordinated ($\kappa^3$) isomer with respect to the $\kappa^4$ form. This is likely to be because the latter is more sterically crowded. Turning to the electronic transitions, describing them in terms of the occupied and virtual molecular orbitals would be tedious because the relevant excited states are too spread out in their composition, affording an unclear picture of the transitions. Moreover, spin-orbit coupling, which results in a complex-valued linear combination of singlet and triplet transitions, adds another layer of complexity to the makeup of the relevant excited states. Essentially, there are five transitions with the highest oscillator strength in the 350–360 nm region. These are excited spinor states 89, 90, 98, 107 and 108, which have their normalized weights based on spin-free transitions, as shown in Table S6. Except for microstate 108, the remaining states have their highest scalar state contributions under 20%, some of which are singlets ($S_{21}$, $S_{24}$) and triplets ($T_{22}$, $T_{25}$). The most summarized view of the character of these spinor transitions may be

visualized by their respective transition difference densities, that is, the difference in the electron density of the occupied MOs involved in the excited state from the electron density of the virtual MOs being occupied. These are displayed in Figure S21, where it can be seen that the lone pairs of the hydroxo moieties play a major role in states 108, 89, and 90. The regions that gain electron density are not surprisingly dominated by the 5f character, although some of the ligands around uranyl are also involved. Without spin-orbit coupling, the most intense states were $S_{21}$ ($\lambda$ = 362, f = 0.076) and $S_{24}$ ($\lambda$ = 354 nm, f = 0.158). The characteristics of these spin-free transitions can be observed in the natural transition orbitals plotted in Figure S21. In this instance, these three transitions cannot be solely classified as LMCT because a significant portion of the ligand is also involved in accepting the electron charge.

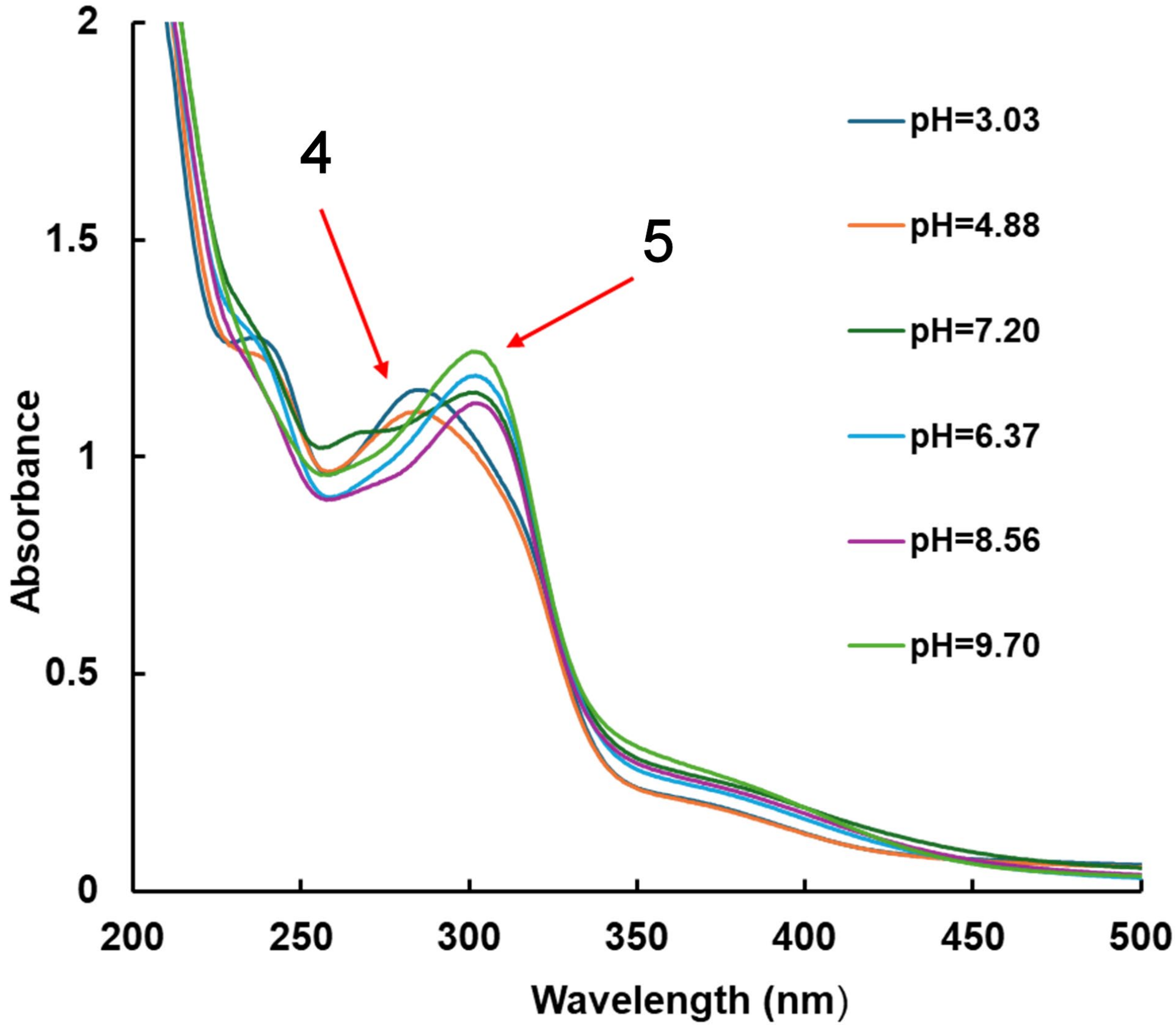


**Figure S27.** UV-Vis Spectra of the aqueous solution of complexes **4** (0.070 mM) and **5** (0.070 mM), at various pH values.

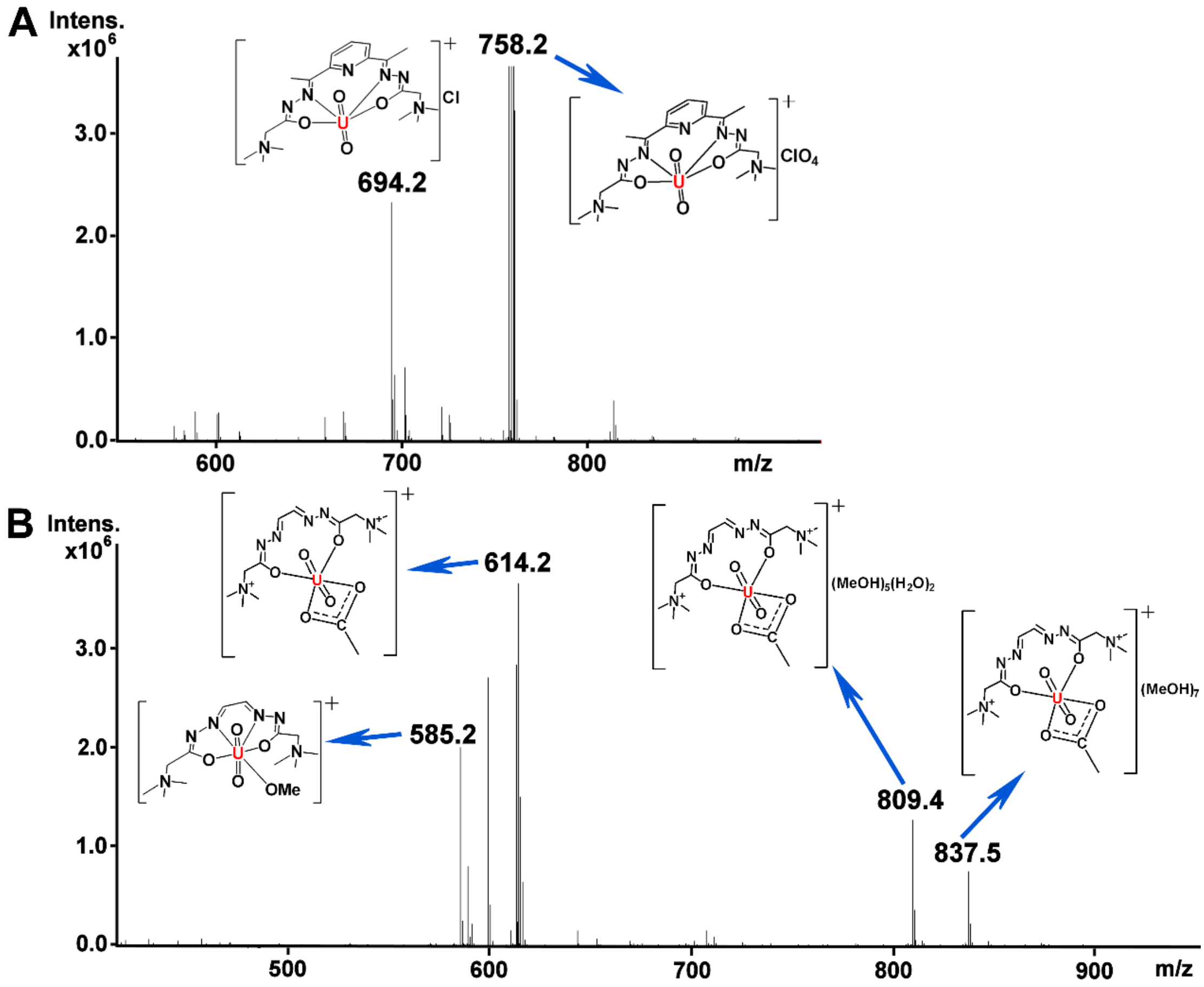


**Figure S28**. ESI-MS of complexes (A) **1** and (B) **3** in $H_2O$:MeOH medium.

*ESI-MS Investigation of the Speciation of **1** and **3–5** in $D_2O$ at Various pDs.* Additional characterization of the solution of $U^{VI}O_2^{2+}$ complexes **1** and **3** with $H_2dapGT^{2+}$ and $H_2glyxGT^{2+}$ as well the $Fe^{III}$ adducts (**4** and **5**) with the $H_2dapGT^{2+}$ ligand was conducted using high-resolution ESI-MS to assess the stability of the compounds in solution. [28-31] The ESI-MS analyses were carried out in a $H_2O$:MeOH medium in the positive ionization mode. In the case of **1** and **3**, two sets of isotopic distribution envelopes within the range of m/z 600–850 were attributed to the presence of intact complexes, which arise from the combination of protons, counterions, and solvent molecules (Figure S23). More specifically, the observed isotopic envelopes centred at

694.2 and 758.2 m/z can be assigned to species with the formulae $[(U^{VI}O_2dapGT)Cl]^+$ and $[(U^{VI}O_2dapGT)ClO_4]^+$.

Similarly, the ESI-MS spectrum of complex **3** revealed a series of singly charged distribution envelopes in the range of m/z 500–900, which corresponds to the intact cluster (Figure S23B). More specifically, the isotopic distribution envelope at m/z 585.2, 614.32, 809.4 and 837.5 can be attributed to the formulae $[(U^{VI}O_2glyxGT)(MeO)]^+$, $[(U^{VI}O_2glyxGT)(AcO)]^+$, $\{[(U^{VI}O_2glyxGT)(AcO)](MeOH)_5(H_2O)_2\}^+$ and $\{[(U^{VI}O_2glyxGT)(AcO)](MeOH)_7\}^+$ respectively.

The ligand $H_2dapGT^{2+}$ demonstrated coordinating efficacy with $Fe^{II}$ metal ions in aqueous solutions, leading to the formation of stable complexes, as discussed in the experimental and X-ray diffraction analysis sections. Compounds **4** and **5** also demonstrated stability in solution, as revealed by ESI-MS studies. More specifically, the spectrum of compound **4** revealed a series of isotopic envelopes in the m/z 500–1000 region of region (Figure S24A). The lower m/z value species centered at m/z 506.5, 515.2 and 579.2 are fragments of the oxo-bridged dimeric species that can occur during the ionization and ion transfer processes during the course of the investigation and can be assigned to the species with formulae, $[(Fe^{III}dapGT)Cl_2]^+$, $[(Fe^{III}dapGT)(MeO)_2]^+$ and $[(Fe^{III}dapGT)(MeO)_2(H_2O)_4]^+$ respectively.

At higher m/z values, an isotopic envelope observed at m/z 1012.2 values can be assigned to compound **4** using the formula $[(Fe^{III}dapGT)_2(MeO)_2(H_2O)_2]^+$. Similarly, the ESI-MS spectrum of compound **5** revealed a singly charged isotopic envelope at

m/z 575.1 which can be assigned to the positively charged species with the formula $[(Fe^{III}dapGT)(MeO)_2(H_2O)_2]^+$ as shown in Figure S24B.

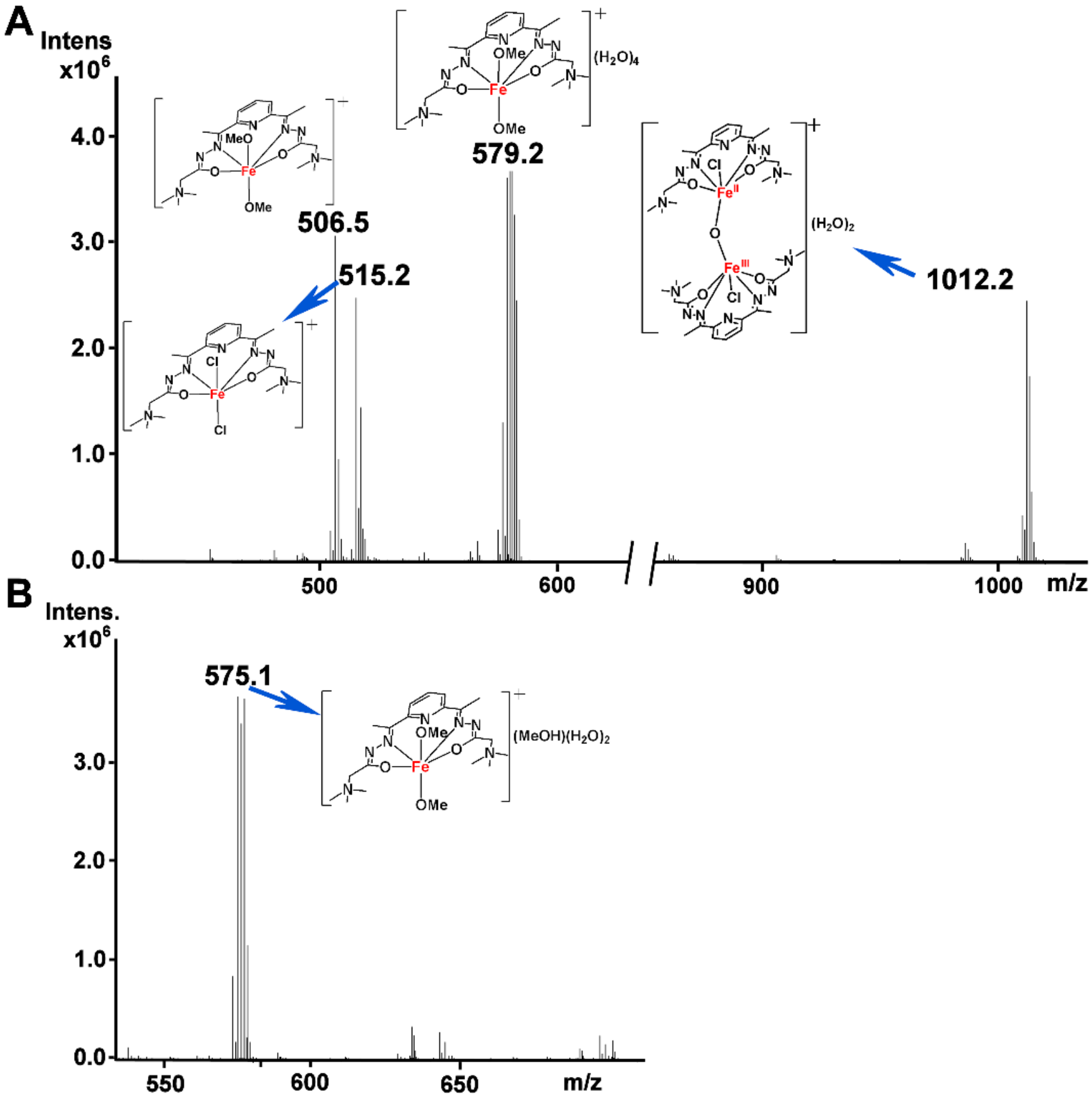


**Figure S29.** ESI-MS of complexes (A) **4** and (B) **5** in $H_2O$:MeOH medium.

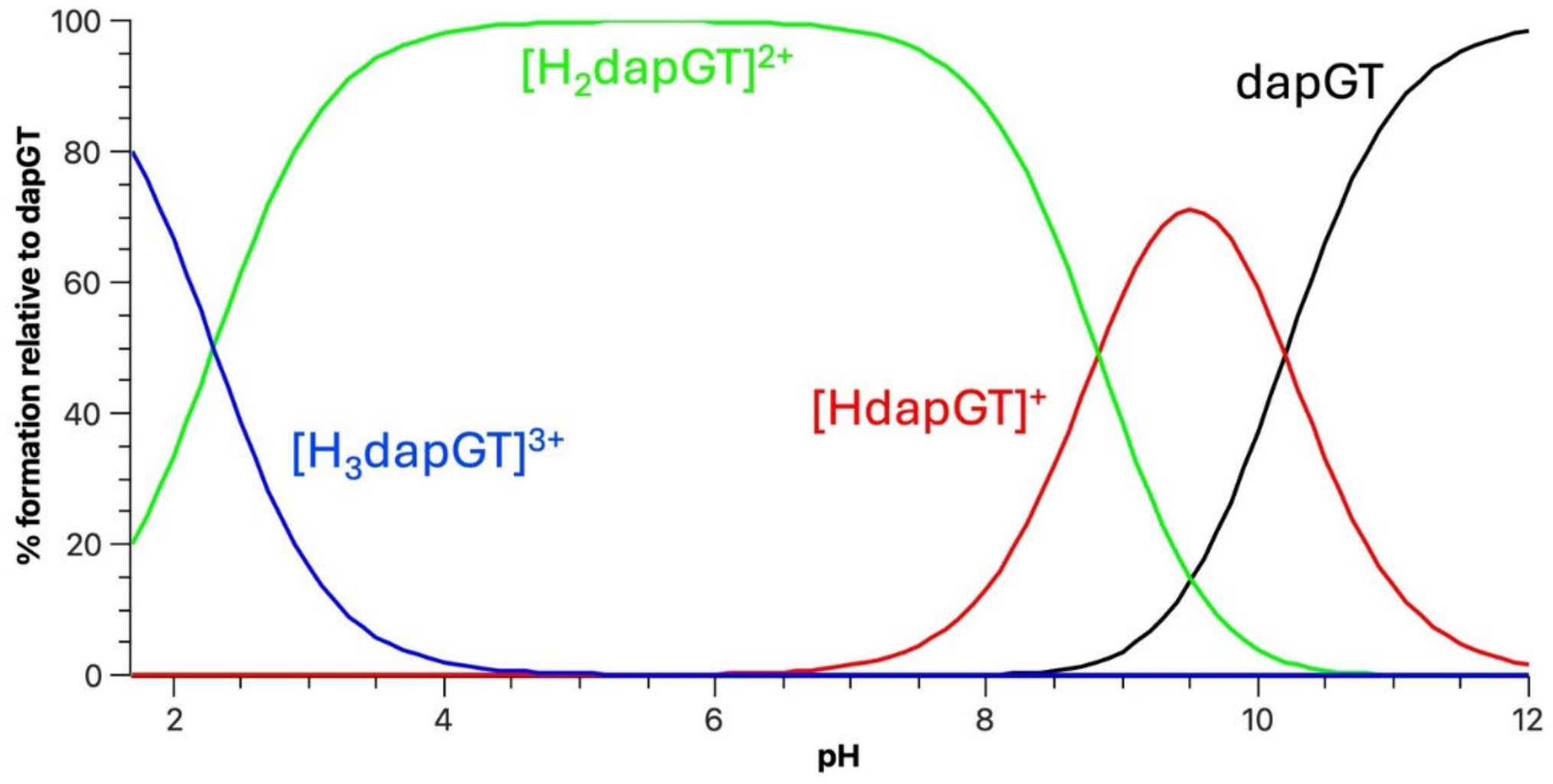


**Figure S30.** Species distribution of dapGT as a function of pH ($c_{dapGT}$ = 3 *mM*, *I* = 0.1 *mM* in NaCl$_{(aq)}$, *T* = 298.15 K)

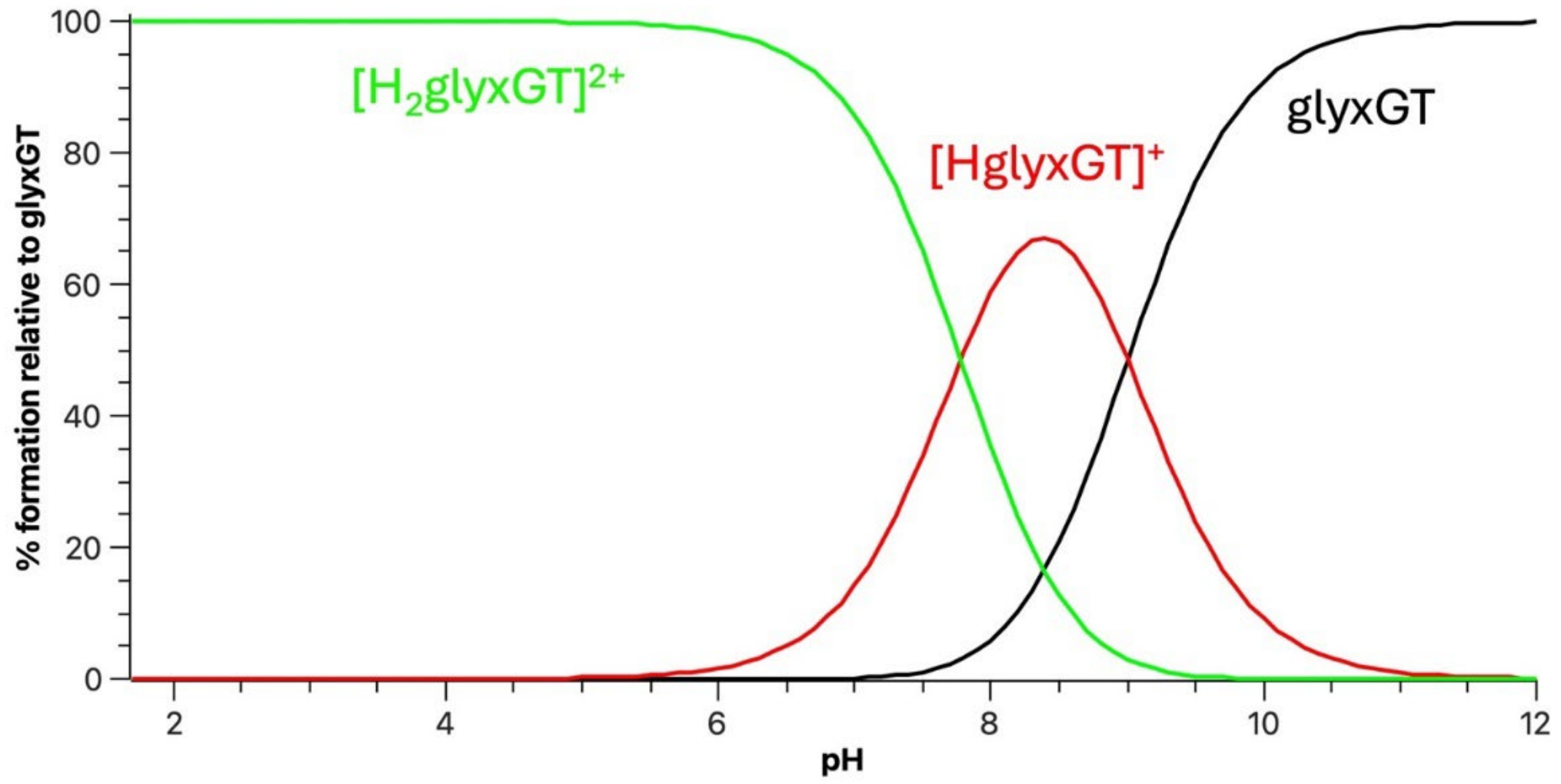


**Figure S31**. Species distribution of glyxGT as a function of pH ($c_{glyxGT}$ = 3 *mM*, *I* = 0.1 *mM* in NaCl$_{(aq)}$, *T* = 298.15 K)

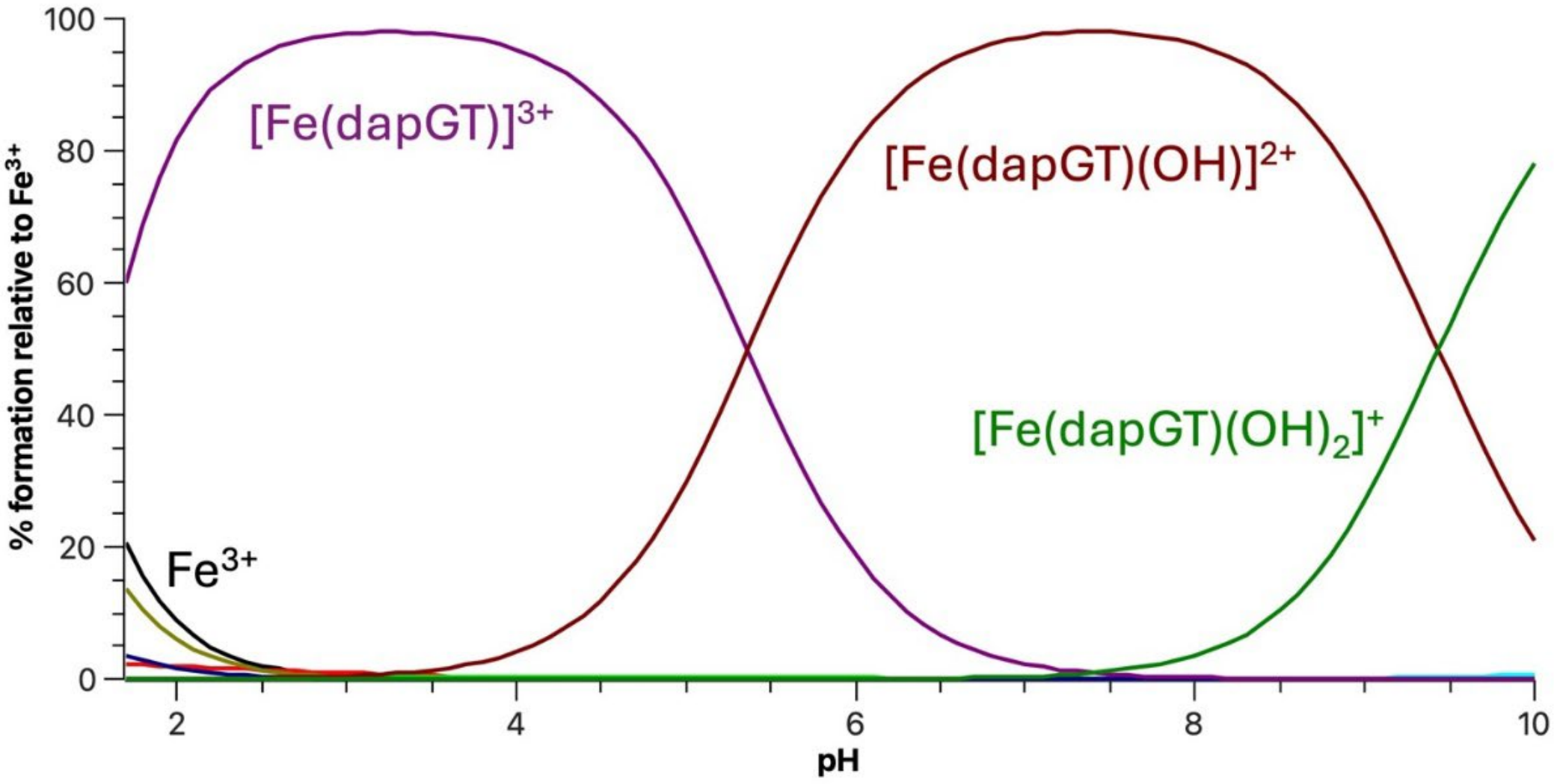


**Figure S32.** Species distribution for $Fe^{III}$/dapGT aqueous system ($c_{FeIII} = c_{dapGT}$ = 3 mM, *I* = 0.1 mM in $NaCl_{(aq)}$, *T* = 298.15 K).

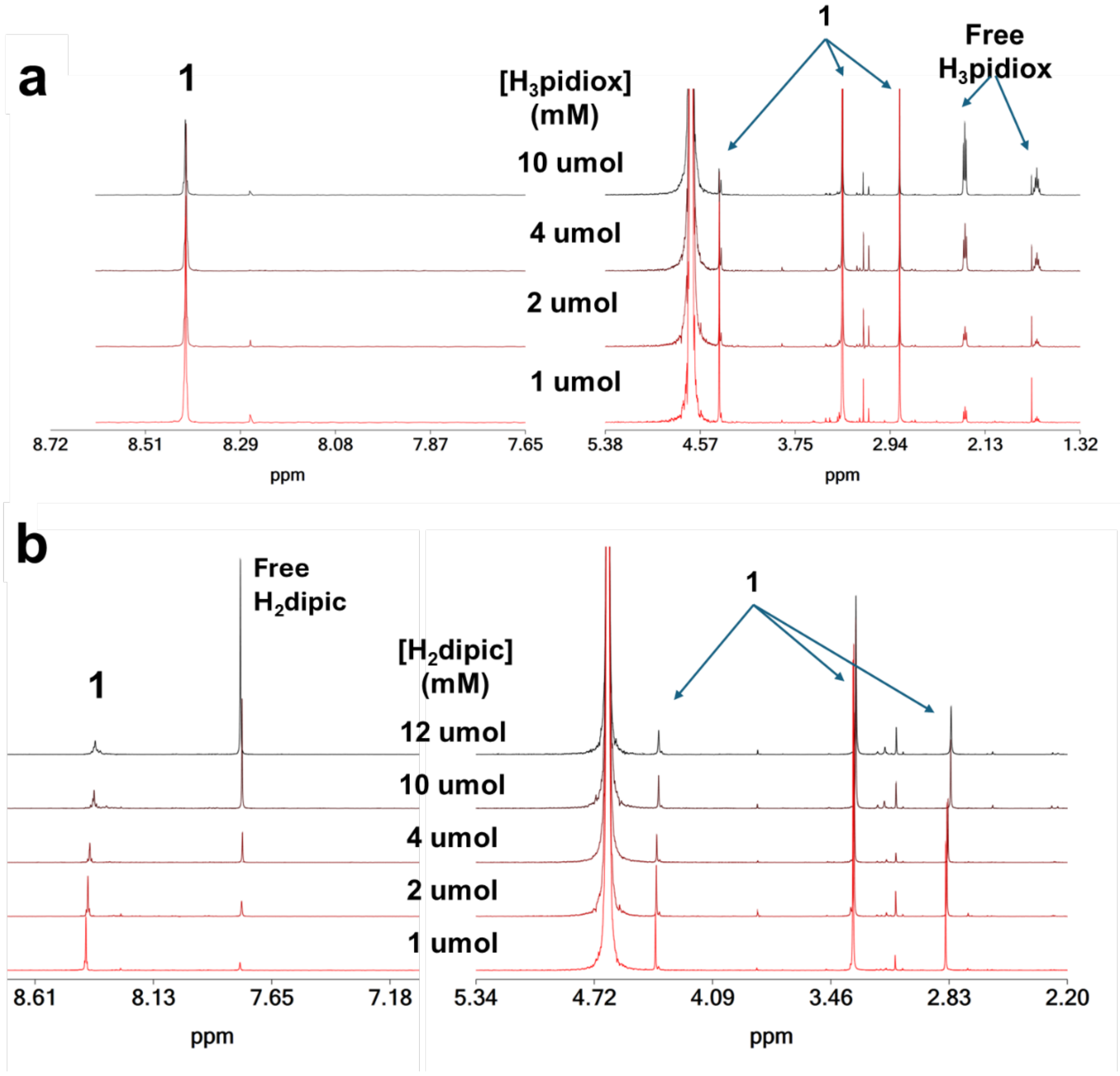


**Figure S33**. $^{1}$H NMR titration spectra of a solution of **1** (4.00 mM) with (A) $H_3$pidiox (1.00–10.0 mM) at pD=9 and (B) $H_2$dipic (1.00–12.0 mM) at pD=9. The spectra of the same solutions at different pD are identical to the specific ones and were thus omitted from the figure.

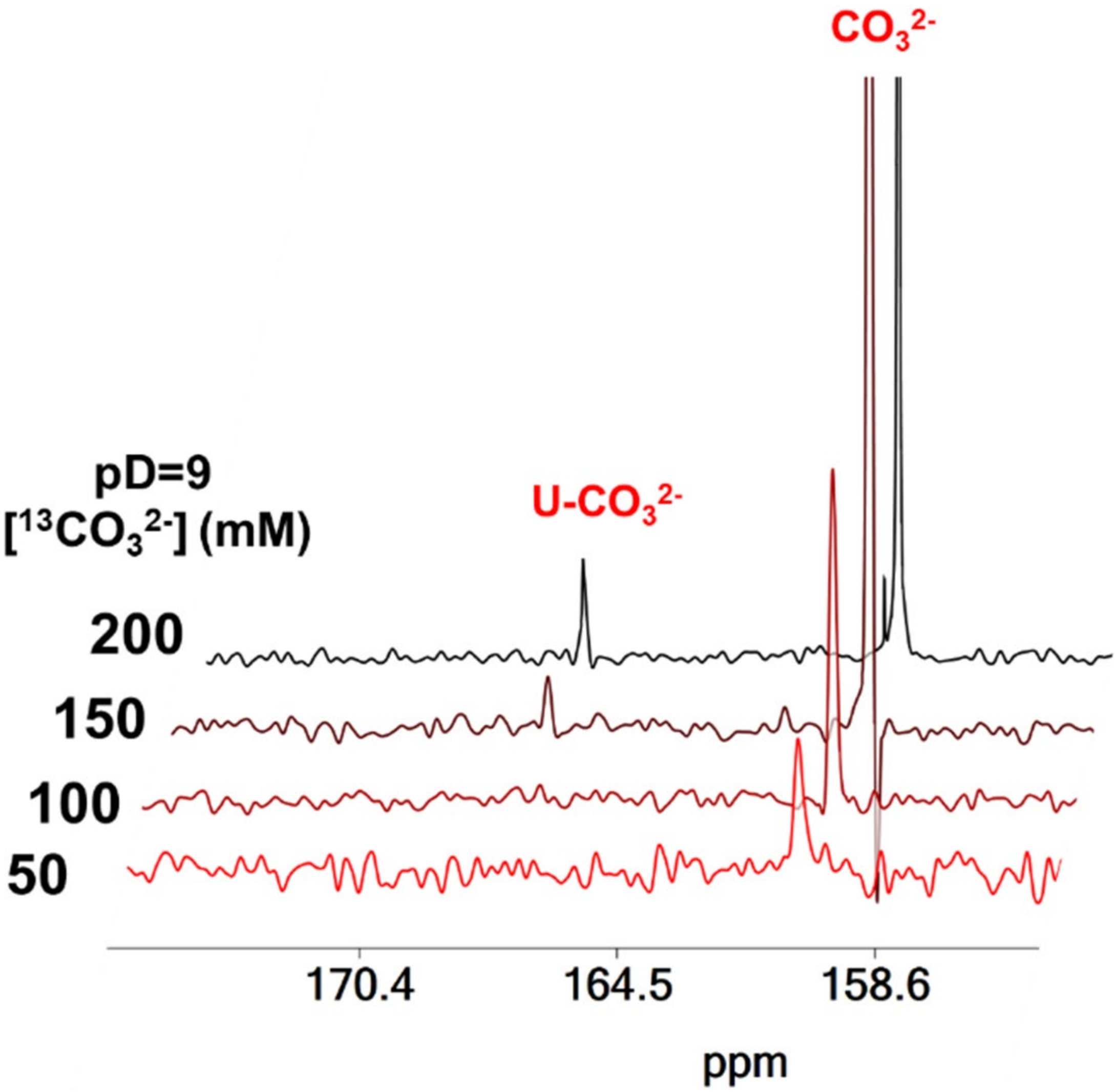


**Figure S34.** $^{13}$C NMR Spectra (A) $^{13}CO_3^{2-}$ (200 mM) + **1** (4.00 mM) at various pDs (B) **1** (4 mM) with increasing concentration of $^{13}CO_3^{2-}$ at pD=9.

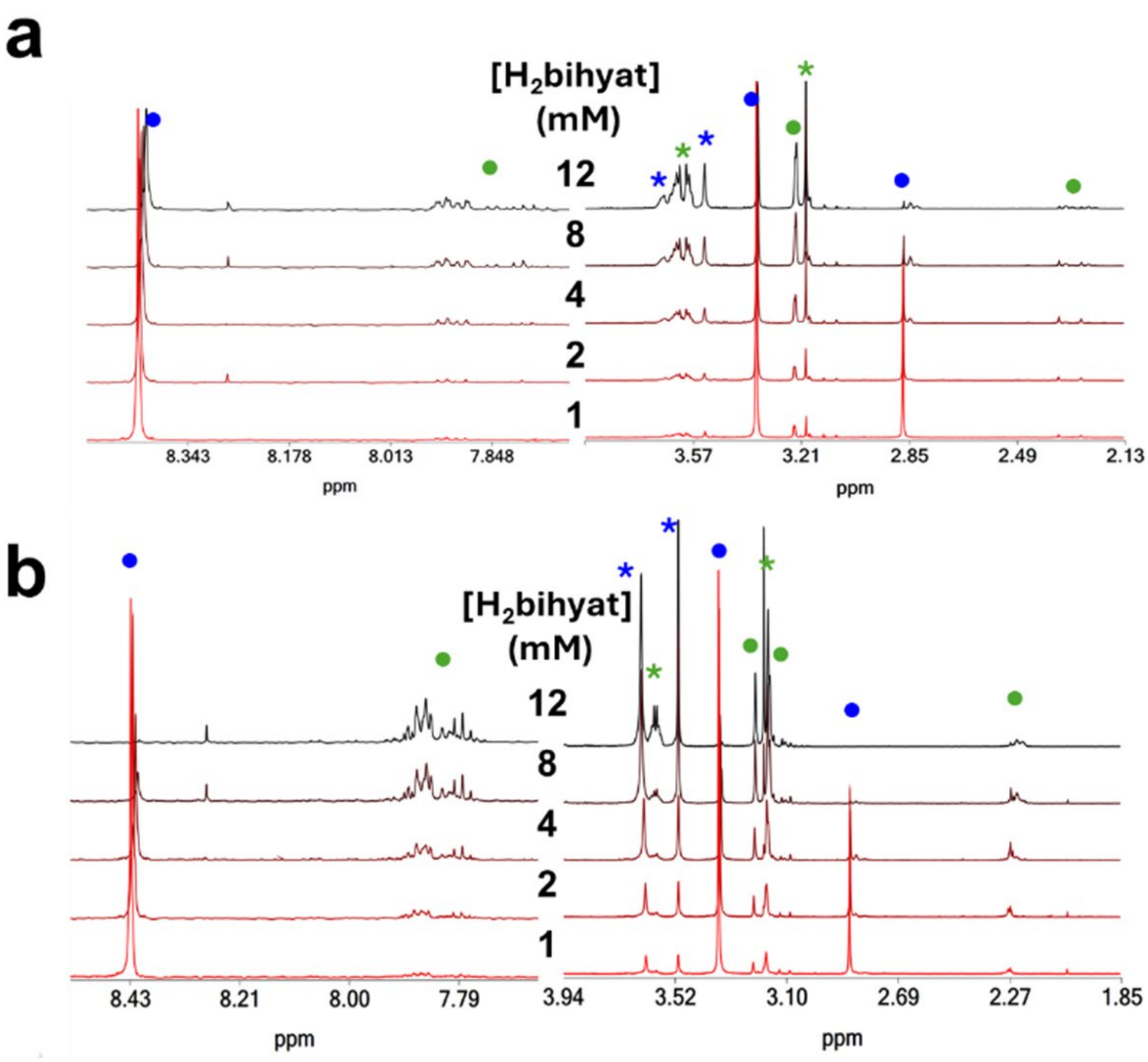


**Figure S35.** $^{1}$H NMR titration spectra of a solution of **1** (4.00 mM) with $H_2$bihyat (1.00–12.0 mM) (A) pD=7, (B) pD=9. ● **1**, ● free $H_2dapGT^{2+}$, * ligated $H_2$bihyat, * free $H_2$bihyat.

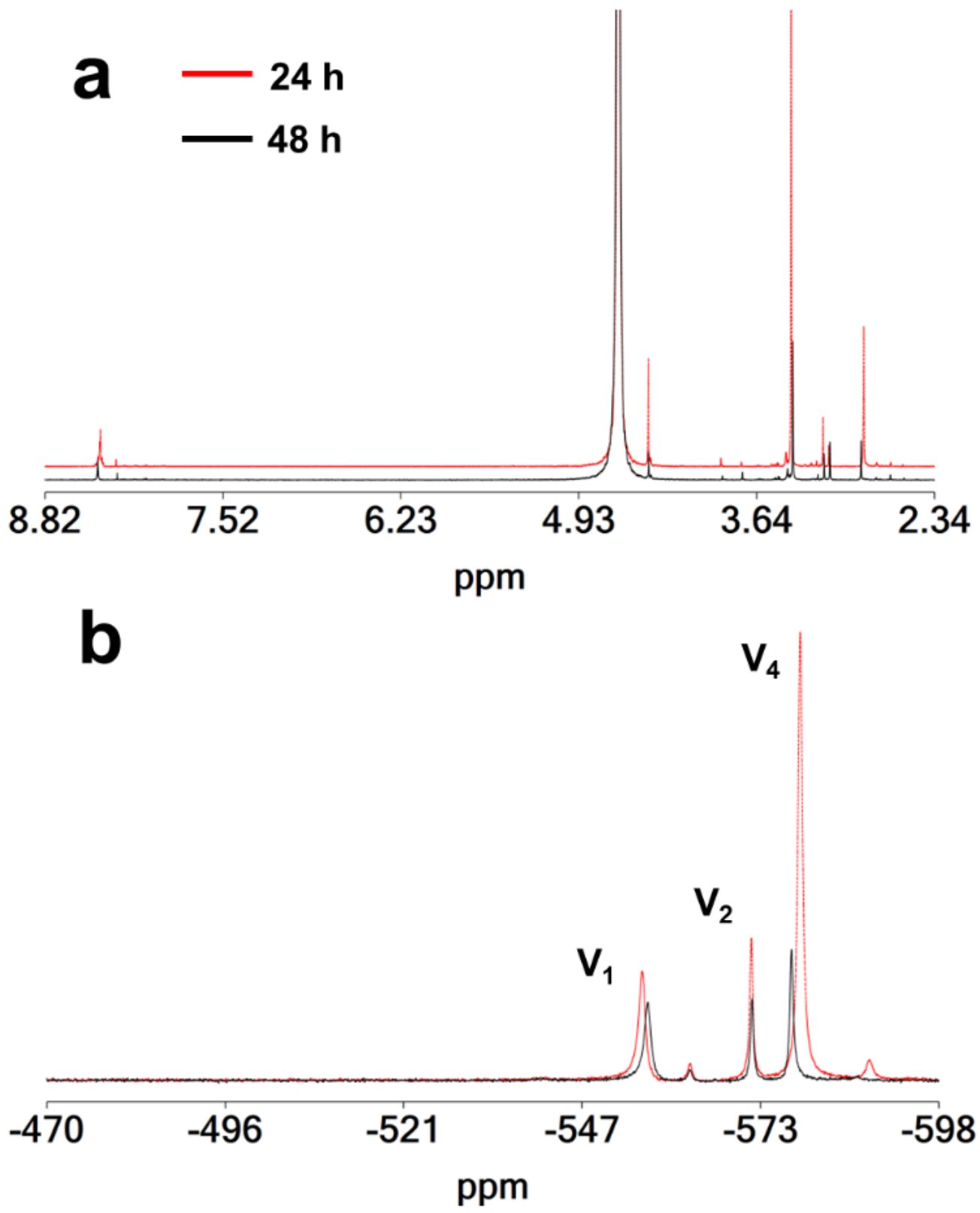


**Figure S36**. NMR Spectra (A) $^{1}H$ NMR and (B) $^{51}V$ NMR aqueous solution (pD=8) of **1** (2.5 mM) and $V^{V}O_2^+$ (5.00 mM) 24 (red line) and 48 (black line) h after mixing.

**Table S1.** Crystallographic parameters for complexes **1**, **1′**, **2** and **3**.

| Parameter | 1 | 1′ | 2 |
|---|---|---|---|
| Empirical formula | $C_{19}H_{33.5}Cl_2N_7O_{13.22}U$ | $C_{20}H_{35}F_{12}N_7O_5Sb_2U$ | $C_{38}H_{94}N_{14}O_{36}U_2V_4$ |
| Formula weight | 880.52 | 1163.08 | 2003.09 |
| Temperature/K | 180.0(3) | 293(2) | 149.98(10) |
| Crystal system | monoclinic | triclinic | triclinic |
| Space group | $P2_1/c$ | P-1 | P-1 |
| a/Å | 9.8510(1) | 9.4662(1) | 9.3537(2) |
| b/Å | 11.9857(2) | 14.3439(1) | 12.1221(3) |
| c/Å | 25.6157(3) | 14.5205(1) | 15.4533(4) |
| α/° | 90 | 64.850(1) | 84.946(2) |
| β/° | 92.1150(10) | 89.416(1) | 87.151(2) |
| γ/° | 90 | 74.540(1) | 82.664(2) |
| Volume/$Å^3$ | 3022.41(7) | 1708.08(3) | 1729.78(7) |
| Z | 4 | 2 | 1 |
| $\rho_{calc}g/cm^3$ | 1.935 | 2.261 | 1.923 |
| $\mu/mm^{-1}$ | 17.395 | 26.641 | 18.201 |
| F(000) | 1713.0 | 1092.0 | 984.0 |
| Crystal size/$mm^3$ | 0.08 × 0.059 × 0.045 | 0.13 × 0.11 × 0.07 | 0.13 × 0.04 × 0.019 |
| Radiation | Cu Kα (λ = 1.54184) | Cu Kα (λ = 1.54184) | Cu Kα (λ = 1.54184) |
| 2Θ range for data collection/° | 6.906 to 154.504 | 6.772 to 153.766 | 5.746 to 152.274 |
| Index ranges | $-12 \le h \le 12, -11 \le k \le 14, -32 \le l \le 31$ | $-11 \le h \le 11, -18 \le k \le 18, -17 \le l \le 18$ | $-11 \le h \le 11, -15 \le k \le 15, -19 \le l \le 15$ |
| Reflections collected | 22044 | 61950 | 19844 |
| Independent reflections | 6188 [$R_{int}$ = 0.0363, $R_{sigma}$ = 0.0334] | 6850 [$R_{int}$ = 0.0455, $R_{sigma}$ = 0.0169] | 6925 [$R_{int}$ = 0.0445, $R_{sigma}$ = 0.0453] |
| Data/restraints/parameters | 6188/1/411 | 6850/3/436 | 6925/23/477 |

| | | | |
|---|---|---|---|
| Goodness-of-fit on $F^2$ | 1.044 | 1.130 | 1.051 |
| Final R indexes [I>=2σ (I)] | $R_1$ = 0.0245, $wR_2$ = 0.0569 | $R_1$ = 0.0214, $wR_2$ = 0.0533 | $R_1$ = 0.0295, $wR_2$ = 0.0733 |
| Final R indexes [all data] | $R_1$ = 0.0285, $wR_2$ = 0.0584 | $R_1$ = 0.0215, $wR_2$ = 0.0533 | $R_1$ = 0.0325, $wR_2$ = 0.0746 |
| Largest diff. peak/hole / e $Å^{-3}$ | 1.41/−0.99 | 0.90/−1.57 | 1.26/−1.67 |

| **Parameter** | **3** |
|---|---|
| Empirical formula | $C_{14}H_{27}ClN_6O_{10}U$ |
| Formula weight | 712.89 |
| Temperature/K | 150.00(10) |
| Crystal system | triclinic |
| Space group | P-1 |
| a/Å | 7.40610(10) |
| b/Å | 13.8299(2) |
| c/Å | 14.4698(2) |
| α/° | 110.607(2) |
| β/° | 103.7200(10) |
| γ/° | 100.4450(10) |
| Volume/$Å^3$ | 1289.27(4) |
| Z | 2 |
| $\rho_{calc}g/cm^3$ | 1.836 |
| $\mu/mm^{-1}$ | 19.173 |
| F(000) | 684.0 |
| Crystal size/$mm^3$ | 0.14 × 0.09 × 0.08 |
| Radiation | Cu Kα (λ = 1.54184) |
| 2Θ range for data collection/° | 6.906 to 151.98 |

| | |
|---|---|
| Index ranges | $-8 \leq h \leq 9$, $-17 \leq k \leq 17$, $-18 \leq l \leq 16$ |
| Reflections collected | 13923 |
| Independent reflections | 5143 [$R_{int}$ = 0.0452, $R_{sigma}$ = 0.0425] |
| Data/restraints/parameters | 5143/0/341 |
| Goodness-of-fit on $F^2$ | 1.030 |
| Final R indexes [I>=2σ (I)] | $R_1$ = 0.0238, $wR_2$ = 0.0567 |
| Final R indexes [all data] | $R_1$ = 0.0247, $wR_2$ = 0.0574 |
| Largest diff. peak/hole / e Å$^{-3}$ | 1.42/−1.13 |

**Table S2**. Selected bond lengths (Å) and angles (°) of **1**, **1′** and **2**.

| | **1** | **1′** | **2** |
|---|---|---|---|
| U1–O1 | 2.405(3) | 2.376(3) | 2.421(3) |
| U1–O2 | 2.403(3) | 2.376(2) | 2.384(3) |
| U1–O3 | 1.769(3) | 1.769(3) | 1.778(3) |
| U1–O4 | 1.771(3) | 1.775(3) | 1.777(3) |
| U1–O5 | 2.603(3) | 2.624(3) | 2.416(3) |
| U1–N1 | 2.570(3) | 2.621(3) | 2.632(4) |
| U1–N2 | 2.571(3) | 2.579(3) | 2.612(4) |
| U1–N7 | 2.607(3) | 2.625(3) | 2.649(4) |
| O1–U1–O5 | 60.33(9) | 58.28(9) | 63.60(11) |
| O1–U1–N7 | 121.23(10) | 120.13(9) | 116.40(11) |
| O1–U1–O2 | 116.78(10) | 118.76(9) | 123.77(11) |
| O1–U1–N2 | 175.60(11) | 174.70(10) | 159.02(13) |
| O1–U1–N1 | 61.11(10) | 59.93(9) | 59.46(11) |
| O2–U1–O5 | 61.44(9) | 61.08(9) | 64.15(11) |
| O2–U1–N7 | 121.80(10) | 121.03(9) | 119.01(11) |
| O2–U1–N2 | 61.26(10) | 60.86(9) | 60.08(11) |
| O2–U1–N1 | 168.42(11) | 173.76(10) | 172.75(12) |
| O3–U1–O4 | 176.31(13) | 177.13(12) | 176.90(15) |
| O3–U1–O1 | 95.43(12) | 90.99(12) | 81.40(14) |
| O3–U1–O5 | 82.05(11) | 96.70(12) | 97.40(14) |
| O3–U1–N7 | 80.29(12) | 88.14(11) | 93.98(14) |
| O3–U1–O2 | 99.01(12) | 88.00(12) | 86.01(14) |

| | | | |
|---|---|---|---|
| O3–U1–N2 | 88.82(12) | 94.26(11) | 78.28(15) |
| O3–U1–N1 | 92.56(12) | 85.94(12) | 101.11(14) |
| O5–U1–N7 | 162.34(10) | 174.87(10) | 168.43(12) |
| O4–U1–O1 | 85.20(13) | 89.75(12) | 101.67(14) |
| O4–U1–O5 | 101.38(12) | 86.05(11) | 84.42(14) |
| O4–U1–N7 | 96.27(12) | 89.09(11) | 84.31(13) |
| O4–U1–O2 | 83.90(12) | 94.07(12) | 92.56(14) |
| O4–U1–N2 | 90.62(13) | 85.03(11) | 98.62(14) |
| O4–U1–N1 | 84.55(13) | 92.04(12) | 80.27(14) |
| N1–U1–O5 | 120.25(10) | 118.19(9) | 115.75(12) |
| N1–U1–N7 | 60.60(10) | 60.30(10) | 59.54(12) |
| N2–U1–O5 | 119.41(10) | 120.24(9) | 124.22(11) |
| N2–U1–N7 | 60.54(10) | 60.83(10) | 60.33(12) |
| N2–U1–N1 | 119.90(10) | 121.09(10) | 119.66(12) |

**Table S3**. Selected bond lengths (Å) and angles (°) of **3**.

| | **3** |
|---|---|
| U1–O3 | 1.781(3) |
| U1–O2 | 2.415(3) |
| U1–O1 | 2.409(3) |
| U1–O4 | 1.782(3) |
| U1–O6 | 2.461(3) |
| U1–O5 | 2.481(3) |
| U1–N1 | 2.597(4) |

| | |
|---|---|
| U1–N2 | 2.594(3) |
| O3–U1–O2 | 87.61(13) |
| O3–U1–O1 | 87.74(13) |
| O3–U1–O4 | 178.84(15) |
| O3–U1–O6 | 91.59(13) |
| O3–U1–O5 | 93.15(13) |
| O3–U1–N1 | 99.56(12) |
| O3–U1–N2 | 89.62(12) |
| O2–U1–O6 | 64.49(11) |
| O2–U1–O5 | 116.52(11) |
| O2–U1–N1 | 119.44(11) |
| O2–U1–N2 | 59.88(11) |
| O1–U1–O2 | 175.13(11) |
| O1–U1–O6 | 117.10(11) |
| O1–U1–O5 | 65.20(11) |
| O1–U1–N1 | 60.02(10) |
| O1–U1–N2 | 118.64(11) |
| O4–U1–O2 | 91.24(14) |
| O4–U1–O1 | 93.41(14) |
| O4–U1–O6 | 88.06(13) |
| O4–U1–O5 | 87.51(13) |
| O4–U1–N1 | 80.87(13) |
| O4–U1–N2 | 89.66(12) |
| O6–U1–O5 | 52.03(11) |

| | |
|---|---|
| O6–U1–N1 | 168.21(11) |
| O6–U1–N2 | 124.25(11) |
| O5–U1–N1 | 122.88(11) |
| O5–U1–N2 | 175.39(10) |
| N2–U1–N1 | 60.13(11) |

**Table S4**. Selected bond lengths (Å) and angles (°) of **4** and **5**.

| | **4** | **5** | |
|---|---|---|---|
| | | **Fe(1)** | **Fe(2)** |
| Fe–O1 | 2.075(3) | 2.105(2) | 2.091(2) |
| Fe–O2 | 2.206(3) | 2.116(2) | 2.119(2) |
| Fe–O3 | - | 1.791(2) | 1.791(2) |
| Fe–O4 | - | 2.215(3) | 2.255(2) |
| Fe–N1 | 2.148(3) | 2.205(3) | 2.228(3) |
| Fe–N2 | 2.222(3) | 2.228(3) | 2.211(3) |
| Fe–N7 | 2.1980(15) | 2.214(3) | 2.234(3) |
| Fe–Cl1 | 2.2959(10) | - | - |
| Fe–Cl2 | 2.3993(10) | - | - |
| O1–Fe–O2 | 77.30(10) | 76.06(10) | 76.60(9) |
| O1–Fe–O4 | - | 83.46(10) | 84.37(11) |
| O1–Fe–N1 | 73.07(11) | 71.42(10) | 71.20(10) |
| O1–Fe–N2 | 147.99(11) | 145.45(10) | 146.93(10) |
| O1–Fe–N7 | 143.49(9) | 139.89(10) | 139.89(10) |
| O2–Fe–O4 | - | 84.81(11) | 84.33(9) |
| O2–Fe–N1 | 150.32(11) | 146.74(10) | 144.80(10) |

| | | | |
|---|---|---|---|
| O2–Fe–N2 | 70.78(10) | 70.86(10) | 70.90(9) |
| O2–Fe–N7 | 138.98(9) | 139.86(10) | 138.75(10) |
| O3–Fe–O1 | - | 98.46(10) | 98.13(10) |
| O3–Fe–O2 | - | 97.42(10) | 98.55(10) |
| O3–Fe–O4 | - | 177.35(11) | 176.54(11) |
| O3–Fe–N1 | - | 94.09(11) | 99.68(10) |
| O3–Fe–N2 | - | 95.43(11) | 92.60(10) |
| O3–Fe–N7 | - | 93.79(10) | 94.27(10) |
| O4–Fe–N2 | - | 83.91(11) | 86.53(10) |
| N1–Fe–O4 | - | 84.77(12) | 78.81(10) |
| N1–Fe–N2 | 138.70(11) | 138.86(10) | 137.52(10) |
| N7–Fe–O4 | - | 83.57(10) | 82.29(10) |
| N1–Fe–N7 | 70.50(10) | 69.71(10) | 69.16(10) |
| N7–Fe–N2 | 68.24(9) | 69.78(10) | 69.48(10) |
| C1–Fe–Cl2 | 178.01(4) | - | - |
| O1–Fe–Cl1 | 93.89(8) | - | - |
| O1–Fe–Cl2 | 87.27(8) | - | - |
| O2–Fe–Cl1 | 91.82(8) | - | - |
| O2–Fe–Cl2 | 86.86(8) | - | - |
| N1–Fe–Cl1 | 91.88(8) | - | - |
| N1–Fe–Cl2 | 90.01(8) | - | - |
| N2–Fe–Cl1 | 89.43(9) | - | - |
| N2–Fe–Cl2 | 88.73(9) | - | - |
| N7–Fe–Cl1 | 89.55(7) | - | - |

| | | | |
|---|---|---|---|
| N7–Fe–Cl2 | 90.47(6) | - | - |

**Table S5.** SOC state 65 composition in the basis of scalar states.

| Spinor state | Largest scalar contributors | *f* (Osc. Strength) | λ(nm) |
|---|---|---|---|
| **65** | 40%$S_{11}$<br>7%$S_{18}$<br>4%$T_{22}$ | 0.134 | 414 |

**Table S6.** Main electronic transitions corresponding to the highest intensity band in $[U^{VI}O_2(OH)_2$-$\kappa^3$-(glyxGT)].

| Spinor state | Largest scalar contributors | *f* (Osc. Strength) | λ(nm) |
|---|---|---|---|
| **89** | 15%$S_{21}$<br>7%$S_{19}$<br>5%$S_{16}$<br>5%$T_{20}$ | 0.039 | 362 |
| **90** | 18%$T_{25}$<br>12%$S_{22}$<br>4%$S_{18}$ | 0.044 | 360 |
| **98** | 12% $T_{22}$<br>10%$S_{18}$<br>8%$T_{26}$<br>8%$T_{27}$ | 0.021 | 355 |
| **107** | 19%$S_{24}$<br>9%$T_{29}$<br>7%$T_{32}$ | 0.037 | 348 |
| **108** | 23%$S_{24}$<br>12%$T_{27}$ | 0.057 | 347 |

**Table S7**. $UO_2^{2+}$ speciation model.

| $nUO_2^{2+} + pCl^- + rac^- + sH^+ \rightleftarrows (UO_2^{2+})_n(Cl^-)_p(ac^-)_r(H^+)_s$ | | |
|---|---|---|
| ***n,p,r,s*** | **species** | **log*β*** |
| 1,0,0,–1 | $[UO_2(OH)]^+$ | –5.25 ± 0.24 [a] |
| 1,0,0,–2 | $UO_2(OH)_2$ | –12.15 ± 0.07 [a] |
| 1,0,0,–3 | $[UO_2(OH)_3]^-$ | –20.25 ± 0.42 [a] |
| 1,0,0,–4 | $[UO_2(OH)_4]^{2-}$ | –32.40 ± 0.68 [a] |
| 2,0,0,–1 | $[(UO_2)_2(OH)]^{3+}$ | –2.7 ± 1.0 [a] |
| 2,0,0,–2 | $[(UO_2)_2(OH)_2]^{2+}$ | –5.62 ± 0.08 [a] |
| 3,0,0,–4 | $[(UO_2)_3(OH)_4]^{2+}$ | –11.9 ± 0.3 [a] |
| 3,0,0,–5 | $[(UO_2)_3(OH)_5]^+$ | –15.75 ± 0.12 [a] |
| 3,0,0,–7 | $[(UO_2)_3(OH)_7]^-$ | –32.2 ± 0.8 [a] |
| 4,0,0,–7 | $[(UO_2)_4(OH)_7]^+$ | –21.9 ± 1.0 [a] |
| 1,1,0,0 | $[UO_2(Cl)]^+$ | 0.17 ± 0.02 [b] |
| 1,2,0,0 | $UO_2(Cl)_2$ | 1.1 ± 0.4 [b] |
| 0,0,1,1 | Hac | 4.755 |
| 1,0,1,0 | $[UO_2(ac)]^+$ | 2.86 [c] |
| 1,0,2,0 | $UO_2(ac)_2$ | 4.62 [c] |
| 1,0,3,0 | $[UO_2(ac)_3]^-$ | 7.22 [c] |
| 1,0,3,–1 | $[UO_2(ac)_3(OH)]^{2-}$ | 2.14 [c] |

*log β from literature at I = 0 M and T = 25 °C:* [a 32], [b33], [c 34]

**Table S8**. $[UO_2(OH)_2]_{(s)}$ solubility product ($\log K_{ps}$) and related equilibrium.

| $(UO_2^{2+})_n(H^+)_s + sH^+ \rightleftarrows nUO_2^{2+} + sH_2O$ | | |
|---|---|---|
| ***n,s*** | **equilibrium** | **log*K*ps** |
| 1, −2 | $UO_2(OH)_{2(s)} + 2H^+ \rightleftarrows UO_2^{2+} + 2H_2O$ | 4.81 ± 0.20 [a] |

*$\log K_{ps}$ from literature at I = 0 M and T = 25 °C:* [a 35]

**Table S9**. $Fe^{III}$ speciation model.

| $n Fe^{3+} + p Cl^- + s H^+ \rightleftarrows (Fe^{3+})_n(Cl^-)_p(H^+)_s$ | | |
|---|---|---|
| ***n,p,s*** | **species** | **log*β*** |
| 1,0,−1 | $[Fe(OH)]^{2+}$ | –2.20 ± 0.02 [a] |
| 1,0,−2 | $[Fe(OH)_2]^+$ | –5.71 ± 0.02 [a] |
| 1,0,−3 | $Fe(OH)_3$ | –12.26 ± 0.26 [a] |
| 1,0,−4 | $[Fe(OH)_4]^-$ | –21.60 ± 0.23 [a] |
| 2 0,−2 | $[Fe_2(OH)_2]^{4+}$ | –2.91 ± 0.07 [a] |
| 3,0,−4 | $[Fe_3(OH)_4]^{5+}$ | –6.3 ± 0.1 [a] |
| 1,1,0 | $[Fe(Cl)]^{2+}$ | 1.52 ± 0.10 [b] |
| 1,2,0 | $[Fe(Cl)_2]^+$ | 2.4 ± 0.3 [b] |

*log β from literature at I = 0 M and T = 25 °C:* [a 36], [b 32]

## References


(1) *SHELXL-97: Program for the Refinement of Crystal Structure*; Sheldrick, G. M., Ed.; University of Göttingen, 1997.
(2) *SHELXS-97: Program for the Solution of Crystal Structure*; Sheldrick, G. M., Ed.; University of Göttingen, 1997.
(3) Zhang, Q.; Fu, S.; Li, H.; Liu, Y. A novel method for the determination of hydrogen peroxide in bleaching effluents by spectroscopy. *BioResources* [Article] **2013**, *8*, 3699-3705. DOI: 10.15376/biores.8.3.3699-3705
(4) Covington, A. K.; Paabo, M.; Robinson, R. A.; Bates, R. G. Use of the glass electrode in deuterium oxide and the relation between the standardized pD (paD) scale and the operational pH in heavy water. *Anal. Chem.* **1968**, *40*, 700-706. DOI: 10.1021/ac60260a013
(5) DE STEFANO, C.; Mineo, P.; Rigano, C.; Sammartano, S. Ionic Strength Dependence of Formation Constants. XVII. The Calculation of Equilibrium Concentrations and Formation Constants. *Annali di Chimica* **1993**, *83*, 243-277
(6) Neese, F.; Wennmohs, F.; Becker, U.; Riplinger, C. The ORCA quantum chemistry program package. *J. Chem. Phys.* **2020**, *152*, 224108. DOI: 10.1063/5.0004608
(7) Neese, F. Software update: The ORCA program system—Version 5.0. *WIREs Comput. Mol. Sci.* **2022**, *12*, e1606. DOI: 10.1002/wcms.1606
(8) Neese, F. The SHARK integral generation and digestion system. *J. Comput. Chem.* **2023**, *44*, 381-396. DOI: 10.1002/jcc.26942
(9) Lechner, M. H.; Papadopoulos, A.; Sivalingam, K.; Auer, A. A.; Koslowski, A.; Becker, U.; Wennmohs, F.; Neese, F. Code generation in ORCA: progress, efficiency and tight integration. *Phys. Chem. Chem. Phys.* [10.1039/D4CP00444B] **2024**, *26*, 15205-15220. DOI: 10.1039/D4CP00444B
(10) Wittmann, L.; Neugebauer, H.; Grimme, S.; Bursch, M. Dispersion-corrected r2SCAN based double-hybrid functionals. *J. Chem. Phys.* **2023**, *159*. DOI: 10.1063/5.0174988 (accessed 1/20/2025).
(11) Furness, J. W.; Kaplan, A. D.; Ning, J.; Perdew, J. P.; Sun, J. Accurate and Numerically Efficient r2SCAN Meta-Generalized Gradient Approximation. *J. Phys. Chem. Lett.* **2020**, *11*, 8208-8215. DOI: 10.1021/acs.jpclett.0c02405

(12) Sun, J.; Ruzsinszky, A.; Perdew, J. P. Strongly Constrained and Appropriately Normed Semilocal Density Functional. *Phys. Rev. Lett.* **2015**, *115*, 036402. DOI: 10.1103/PhysRevLett.115.036402
(13) Peng, D.; Reiher, M. Local relativistic exact decoupling. *J. Chem. Phys.* **2012**, *136*, 244108. DOI: 10.1063/1.4729788 (accessed 1/20/2025).
(14) Sikkema, J.; Visscher, L.; Saue, T.; Iliaš, M. The molecular mean-field approach for correlated relativistic calculations. *J. Chem. Phys.* **2009**, *131*, 124116. DOI: 10.1063/1.3239505 (accessed 1/20/2025).
(15) Peng, D.; Middendorf, N.; Weigend, F.; Reiher, M. An efficient implementation of two-component relativistic exact-decoupling methods for large molecules. *J. Chem. Phys.* **2013**, *138*, 184105. DOI: 10.1063/1.4803693 (accessed 1/20/2025).
(16) Pollak, P.; Weigend, F. Segmented Contracted Error-Consistent Basis Sets of Double- and Triple-ζ Valence Quality for One- and Two-Component Relativistic All-Electron Calculations. *J. Chem. Theory Comput.* **2017**, *13*, 3696-3705. DOI: 10.1021/acs.jctc.7b00593
(17) Pantazis, D. A.; Neese, F. All-Electron Scalar Relativistic Basis Sets for the Actinides. *J. Chem. Theory Comput.* **2011**, *7*, 677-684. DOI: 10.1021/ct100736b
(18) Neese, F. An improvement of the resolution of the identity approximation for the formation of the Coulomb matrix. *J. Comput. Chem.* **2003**, *24*, 1740-1747. DOI: 10.1002/jcc.10318
(19) Neese, F.; Wennmohs, F.; Hansen, A.; Becker, U. Efficient, approximate and parallel Hartree–Fock and hybrid DFT calculations. A 'chain-of-spheres' algorithm for the Hartree–Fock exchange. *Chem. Phys.* **2009**, *356*, 98-109. DOI: 10.1016/j.chemphys.2008.10.036
(20) Izsák, R.; Neese, F. An overlap fitted chain of spheres exchange method. *J. Chem. Phys.* **2011**, *135*, 144105. DOI: 10.1063/1.3646921 (accessed 10/29/2024).
(21) Neese, F.; Olbrich, G. Efficient use of the resolution of the identity approximation in time-dependent density functional calculations with hybrid density functionals. *Chem. Phys. Lett.* **2002**, *362*, 170-178. DOI: https://doi.org/10.1016/S0009-2614(02)01053-9
(22) Stoychev, G. L.; Auer, A. A.; Neese, F. Automatic Generation of Auxiliary Basis Sets. *J. Chem. Theory Comput.* **2017**, *13*, 554-562. DOI: 10.1021/acs.jctc.6b01041
(23) Garcia-Ratés, M.; Neese, F. Effect of the Solute Cavity on the Solvation Energy and its Derivatives within the Framework of the Gaussian Charge Scheme. *J. Comput. Chem.* **2020**, *41*, 922-939. DOI: 10.1002/jcc.26139 (accessed 2024/09/10).
(24) Barone, V.; Cossi, M. Quantum Calculation of Molecular Energies and Energy Gradients in Solution by a Conductor Solvent Model. *J. Phys. Chem. A* **1998**, *102*, 1995-2001. DOI: 10.1021/jp9716997
(25) Hirata, S.; Head-Gordon, M. Time-dependent density functional theory within the Tamm-Dancoff approximation. *Chem. Phys. Lett.* **1999**, *314*, 291. DOI: 10.1016/S0009-2614(99)01149-5
(26) Heß, B. A.; Marian, C. M.; Wahlgren, U.; Gropen, O. A mean-field spin-orbit method applicable to correlated wavefunctions. *Chem. Phys. Lett.* **1996**, *251*, 365-371. DOI: 10.1016/0009-2614(96)00119-4
(27) Neese, F. Efficient and accurate approximations to the molecular spin-orbit coupling operator and their use in molecular g-tensor calculations. *J. Chem. Phys.* **2005**, *122*, 034107. DOI: 10.1063/1.1829047
(28) Miras, H. N.; Long, D. L.; Kögerler, P.; Cronin, L. Bridging the gap between solution and solid state studies in polyoxometalate chemistry: Discovery of a family of [V1M 17]-based cages encapsulating two {VVO4} moieties. *Dalton Trans.* [Article] **2008**, 214-221. DOI: 10.1039/b714285d
(29) Miras, H. N.; Stone, D.; Long, D. L.; McInnes, E. J. L.; Kögerler, P.; Cronin, L. Exploring the structure and properties of transition metal templated {VM17(VO4)2} dawson-like capsules. *Inorg. Chem.* [Article] **2011**, *50*, 8384-8391. DOI: 10.1021/ic200943s
(30) Miras, H. N.; Wilson, E. F.; Cronin, L. Unravelling the complexities of inorganic and supramolecular self-assembly in solution with electrospray and cryospray mass spectrometry. *Chem. Commun.* [Article] **2009**, 1297-1311. DOI: 10.1039/b819534j

(31) Yan, J.; Gao, J.; Long, D. L.; Miras, H. N.; Cronin, L. Self-assembly of a nanosized, saddle-shaped, solution-stable polyoxometalate anion built from pentagonal building blocks: [H 34W119Se8Fe2O420] 54. *J. Am. Chem. Soc.* [Article] **2010**, *132*, 11410-11411. DOI: 10.1021/ja104043s
(32) Tagirov, B. R.; Diakonov, I. I.; Devina, O. A.; Zotov, A. V. Standard ferric-ferrous potential and stability of FeCl2+ to 90°C. Thermodynamic properties of Fe((aq))3+ and ferric-chloride species. *Chem. Geol.* [Article] **2000**, *162*, 193-219. DOI: 10.1016/S0009-2541(99)00150-3
(33) Berto, S.; Crea, F.; Daniele, P. G.; Gianguzza, A.; Pettignano, A.; Sammartano, S. Advances in the investigation of dioxouranium(VI) complexes of interest for natural fluids. *Coord. Chem. Rev.* [Review] **2012**, *256*, 63-81. DOI: 10.1016/j.ccr.2011.08.015
(34) Berto, S.; Crea, F.; Daniele, P. G.; De Stefano, C.; Prenesti, E.; Sammartano, S. Dioxouranium(VI) - carboxylate complexes. Interaction with dicarboxylic acids in aqueous solution: Speciation and structure. *Annali di Chimica* [Article] **2006**, *96*, 399-420. DOI: 10.1002/adic.200690042
(35) Brown, P. L.; Ekberg, C. Actinide Metals. In *Hydrolysis of Metal Ions*2016; pp 325-432. https://doi.org/10.1002/9783527656189.ch9 DOI: https://doi.org/10.1002/9783527656189.ch9 (accessed 2025/07/03).
(36) Brown, P. L.; Ekberg, C. First Transition Series Metals. In *Hydrolysis of Metal Ions*2016; pp 499-716. https://doi.org/10.1002/9783527656189.ch11 DOI: https://doi.org/10.1002/9783527656189.ch11 (accessed 2025/07/03).
